# Effect of Dendrimer Generation and Aglyconic Linkers on the Binding Properties of Mannosylated Dendrimers Prepared by a Combined Convergent and Onion Peel Approach

**DOI:** 10.3390/molecules23081890

**Published:** 2018-07-28

**Authors:** Celia Sehad, Tze Chieh Shiao, Lamyaa M. Sallam, Abdelkrim Azzouz, René Roy

**Affiliations:** 1 Department of Chemistry, University of Québec a Montréal, P.O. Box 8888, Succ. Centre-Ville, Montréal, QC H3C 3P8, Canada; celia_0709@hotmail.fr (C.S.); chichi_shiao@hotmail.com (T.C.S.); lsallam@yahoo.com (L.M.S.); azzouz.a@uqam.ca (A.A.); 2 Glycovax Pharma Inc., 424 Guy, Suite 202, Montreal, QC H3J 1S6, Canada

**Keywords:** dendrimers, mannose, click chemistry, *E. coli*, UTIs, FimH, DLS

## Abstract

An efficient study of carbohydrate-protein interactions was achieved using multivalent glycodendrimer library. Different dendrimers with varied peripheral sugar densities and linkers provided an arsenal of potential novel therapeutic agents that could be useful for better specific action and greater binding affinities against their cognate protein receptors. Highly effective click chemistry represents the basic method used for the synthesis of mannosylated dendrimers. To this end, we used propargylated scaffolds of varying sugar densities ranging from 2 to 18 for the attachment of azido mannopyranoside derivatives using copper catalyzed click cycloaddition. Mannopyranosides with short and pegylated aglycones were used to evaluate their effects on the kinetics of binding. The mannosylated dendrons were built using varied scaffolds toward the accelerated and combined “onion peel” strategy These carbohydrates have been designed to fight *E. coli* urinary infections, by inhibiting the formation of bacterial biofilms, thus neutralizing the adhesion of FimH type 1 lectin present at the tip of their fimbriae against the natural multiantennary oligomannosides of uroplakin 1a receptors expressed on uroepithelial tissues. Preliminary DLS studies of the mannosylated dendrimers to cross- link the leguminous lectin Con A used as a model showed their high potency as candidates to fight the *E. coli* adhesion and biofilm formation.

## 1. Introduction

Modern medicine is exploring several alternative strategies to overcome the expending antibiotic resistance problems. One of these goals is to find new ways to fight bacterial infections. Of particular interest are the recurrent urinary tract infections (UTIs). Half of women are affected by UTIs at least once in their lifetime. Hence, UTIs represent an important public health issue because of their frequent occurrence. UTIs are mostly caused by uropathogenic *Escherichia coli* strains (UPEC) that infest urinary epithelium through their type-1 pili (FimH). At the tips of their pili, *E. coli* possesses a carbohydrate binding domain called FimH that adhere to mannosylated glycoproteins receptors on the urinary epithelial cells [1,2,3]. In addition, through their quorum sensing molecules, *E. coli* can infest hosts cell and forms resilient biofilms. Once formed, bacteria become mature and readily acquire drugs resistance.

Besides, as the first defense mechanism against this infectious agent, the innate immune system exploits the structures of mannosides [4,5]. On the other hand, fimbriated *E. coli* receptor-binding site (FimH) consists of a specific mannose-binding pocket, with a tyrosine gate (Tyr48, Tyr137) [6,7,8] that also recognizes and binds to the mannosides residues of uroplakin-1a, present at the surface of urothelial cells as a premise for bacterial infections causing cystitis and pyelonephritis. Ever since, considerable efforts have been devoted toward the development of new synthetic antiadhesive antagonists that could act as potent competitive inhibitors [9,10,11,12,13]. Since then, synthetic mono-mannopyranosides carrying various aglycones have been reported and their binding to FimH have been fully characterized. Amongst these, heptyl α-d-mannopyranoside (HM) as emerged as one of the strongest monovalent FimH binders described so far [3]. The studies supported that the hydrophobic HM’s aglycone strongly interacted via van der Waals contacts and aromatic stacking with the tyrosine gates residues Tyr148 and Tyr137 [2,8,13].

However, monomeric carbohydrate residues interacting with proteins often occur with low-binding affinities [14,15,16]. Furthermore, multivalent carbohydrate-protein interactions have unequivocally displayed several advantages over monomeric ones, a process commonly used by nature to control a wide variety of cellular processes [17,18,19]. Hence, several glycodendrimers have been designed to address the issue of low affinity carbohydrate-protein interactions [5,20,21,22,23,24,25,26,27]. Mannosylated glycodendrimers were found to be excellent ligands against the leguminous lectin from *Canavalia ensiformis* (Concanavalin A) and to be excellent ligands toward the fimbriated *E. coli* K12 with nanomolar affinities [21,26,28,29]. Likewise, the glycoside cluster effect helps to enhance avidity, selectivity and affinity of a multivalent glycoside toward a protein that possess multiple carbohydrate recognition domains (CRDs). This phenomenon was characterized by strong stabilization of carbohydrate-protein interactions through macroscopic cross-linking effects [17,19]. Therefore, mannosylated glycodendrimers constitute excellent lead candidates for the treatment of *E. coli* infectious agents by the inhibition of bacterial adhesion and biofilm formation on cell surfaces [5,12,30].

To these aim, a new variety of mannosylated dendrimers of different sugar densities and linker’s properties have been developed to prevent infections spreading, by inhibiting *E. coli* adhesion and biofilm formations. Thus, we report herein a new class of glycodendrimers [5,20,21,22,25,28,31,32] that are built on aromatics, cyclotriphosphazene and pentaerythritol cores using high yielding click chemistry to anchor mannosylated azides onto the above alkyne-functionalized scaffolds. This synthetic strategy provides easy access to biodegradability and biocompatibility to the glycodendrimers. In addition, by using different scaffolds at each layer rather than the identical ones commonly used, we are providing another application of the so called “onion peel” approach. Hence, pegylated tetraethylene glycol spacers have been introduced as aglycones, because of the easy availability, water-solubility, biocompatibility and the variable chain length accessible that should enable good interactions with the multiple FimH binding pili present at the surface of *E. coli*. The relative affinities of the pegylated mannodendrimers were studied using diffraction light scattering measurements (DLS) in the presence of ConA, toward a better understanding of the multivalent binding interactions and the effect of the linkers. Although ConA is a leguminous lectin, it also binds natural multiantennary mannopyranosides that can also be advantageously replaced by synthetic monomeric derivatives harboring hydrophobic aglycones. Hence, it constitutes an appropriate model to evaluate the relative propensity of mannosylated dendrimers toward *E. coli* FimH.

## 2. Results and Discussion

### 2.1. Scaffold Syntheses

**Scheme 1:**
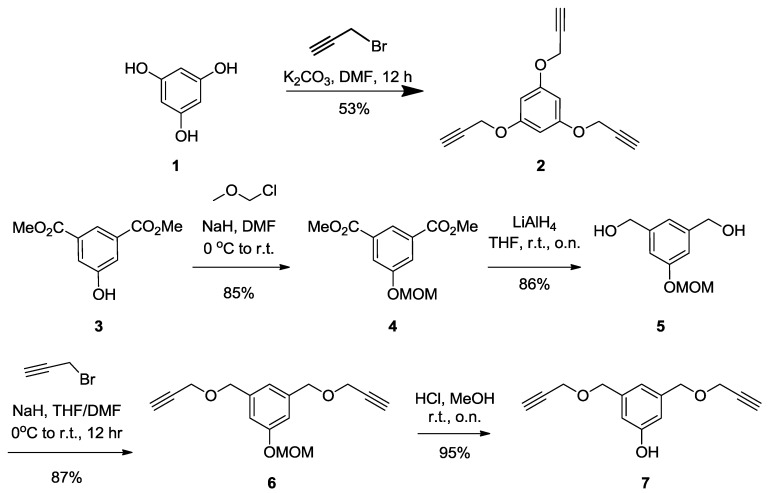
Synthesis of *tris*-propagylated **2** and bis-propagylated **7** aromatic scaffolds.

First, the syntheses of different propargylated aromatic scaffolds with valencies of 2 (**7**) and 3 (**2**) were achieved in moderate to good yields. Scaffolds **2** and **7** were obtained through propargylation (propargyl bromide, K_2_CO_3_, DMF) of polyhydroxylated aromatic cores **1** and **5**, respectively. The trimeric phloroglucinol **1** was commercially available and the dimeric compound **5** was synthesized after protection of the hydroxyl group of 5-hydroxy dimethyl isophthalate **3** with a MOM-protecting group (MOMCl, NaH, DMF, 85%), followed by reduction of the intermediate esters of **4** to afford **5** using LiAlH_4_ in 86% yield (Scheme 1).

**Scheme 2:**
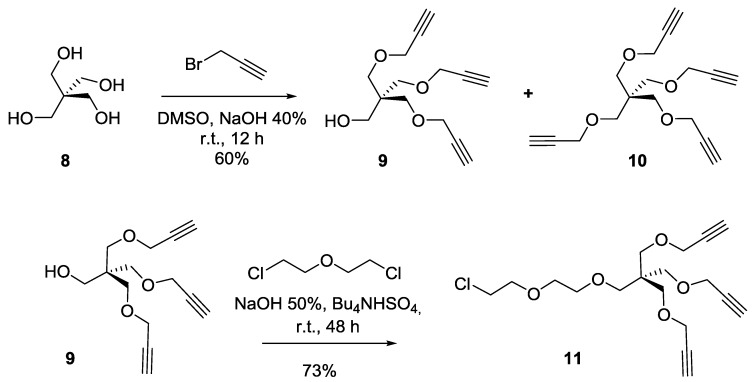
Synthesis of a tripropagylated dendron precursor **11** under PTC conditions.

Pentaerythritol **8** was next chosen as template for the synthesis of the desired *tris*-propargylated core **9** (propargyl bromide, DMSO/NaOH) (Scheme 2). It was accompanied by smaller quantities of the fully tetra-propargylated scaffold **10** which was readily separated by flash chromatography. The reaction mixture gave both **9** and **10** in a 2:1 ratio, respectively. An extended dendron precursor **11** was then obtained from **9** using bis(2-chloroethyl) ether under phase-transfer catalyzed conditions (PTC) (tetrabutylammonium hydrogensulfate (TBAHS), NaOH, r.t.) in 73% yield (Scheme 2).

**Scheme 3:**
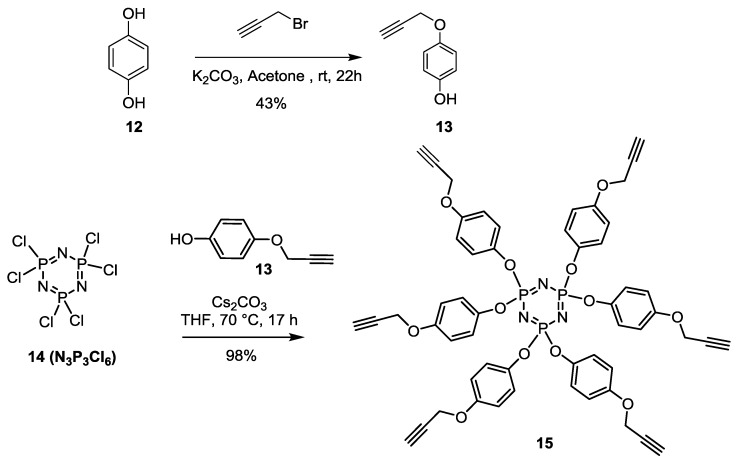
Synthesis of a hexa-propargylated cyclotriphosphazene core **15**.

For our next scaffold, harboring six propargylated functionalities, we choose the previously described hexachlorocyclotriphosphazene core **14** (N_3_P_3_Cl_6_) [28,33,34,35]. The choice for this dense hexavalent core was based on previous observations by the Majoral’s group who showed that it was biologically favorable over several other scaffolds and that it can readily form dendrimers of very high generation [36,37,38,39,40]. Toward this goal, diol **12** was monopropargylated (propargyl bromide, K_2_CO_3_, acetone, r.t., 12 h) to afford **13** (43%) (Scheme 3) which was treated with **14** under basic conditions (Cs_2_CO_3_, THF, 70 °C, 17 h) to provide **15** in essentially quantitative yield (98%). The structural integrity of the fully substituted **15** was compared to its known ^1^H- and ^31^P-NMR [28].

### 2.2. Sugar Precursors

**Scheme 4:**
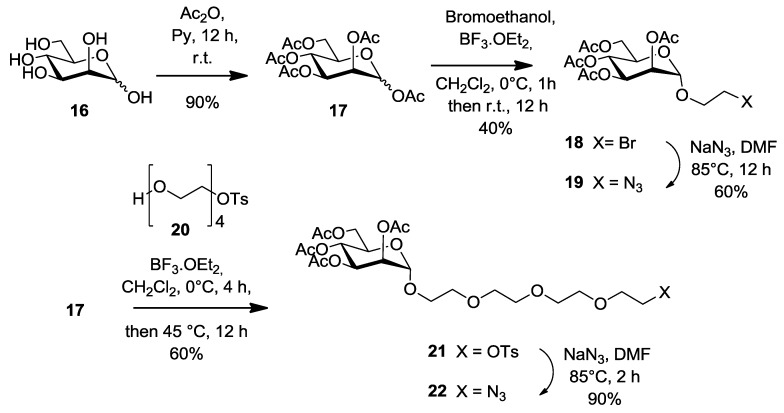
Synthesis of azido mannoside precursors having a short (**19**) and a long spacer (**22**).

It is well known that the aglyconic moiety of a given carbohydrate can affect the binding affinity and selectivity with lectins. This effect is achieved by either providing better accessibility of the sugar itself into the deep binding pocket of the lectin (linker effect) or by increasing the binding interactions network between neighboring amino acids (pharmacophore effect). Because of the high number of pili at the surface of *E. coli*, we figured that the linker effect would be a dominant aspect. For comparison purposes, we also built our mannoside residues with a short linker. Thus, known peracetylated mannose **17** was chosen as a starting point after treatment of commercial d-mannose **16** with acetic anhydride in pyridine (Scheme 4). Lewis acid treatment of **17** with bromoethanol in DCM (BF_3_-Et_2_O, DCM, 40%) provided known 2-bromoethyl α-d-mannopyranoside **18**. Nucleophilic substitution of the bromide by an azide group (NaN_3_, DMF, 85 °C, 12 h) afforded 2-azidoethyl α-d-mannopyranoside **19** in 60% yield. Similarly, using monotosylated tetraethylene glycol as the aglycone (**20**), analogous glycosidation of **17** gave tosylate **21** (60%), followed by displacement with azide to give extended mannoside **22** in 90% yield.

### 2.3. Glycodendron Syntheses

**Scheme 5:**
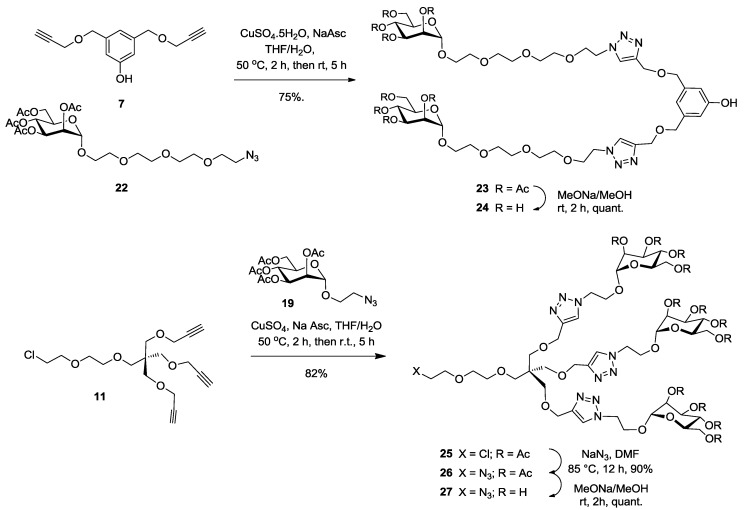
Synthesis of dimeric (**24**) and trimeric (**27**) mannodendrons using click chemistry.

Having the di-(**7**) and tri-propargylated (**11**) scaffolds in hands, together with the two family of short (**19**) and long (**22**) azido mannosides, we were set for the convergent syntheses of our mannosylated dendrons using the efficacious [1,3]-dipolar copper-catalyzed azide-alkyne cycloaddition (CuAAC) (click chemistry) (Scheme 5). We first combined bis-propargylated scaffold **7** with elongated azido mannoside **22** under typical CuAAC conditions to obtain dimer **23** in 75% yield. Alternatively, the tri-mannosylated dendron was accomplished by treating chloride **11** with the short mannoside **19** under the above click conditions to provide dendron **25** (82%) having an orthogonal chloride functionality at the focal point. Displacement of the chloride by an azide as above gave **26** in 90% yield. Final sugar deprotection using the Zemplén transesterification procedure (NaOMe, MeOH) gave the necessary precursors **24** and **27** in quantitative yields. Completion of the reactions were evidenced by proton NMR spectroscopy, wherein complete disappearance of the propargylic CH signal at δ 2.40 ppm and the appearance of the newly formed singlet of the triazole moiety at δ 7.65 ppm.

### 2.4. Glycodendrimer Syntheses

**Scheme 6:**
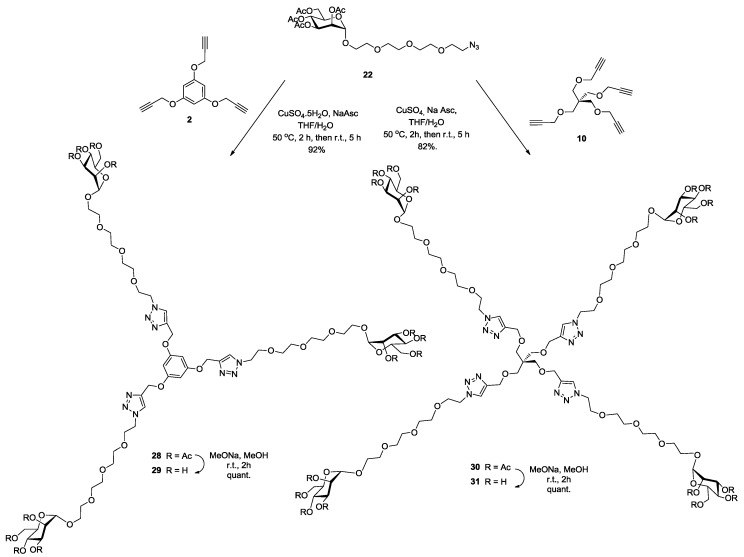
Synthesis of tri- (**29**) and tetra-valent (**31**) pegylated mannosylated clusters using click chemistry on trimer (**2**) and tetramer (**10**) scaffolds, respectively.

**Scheme 7:**
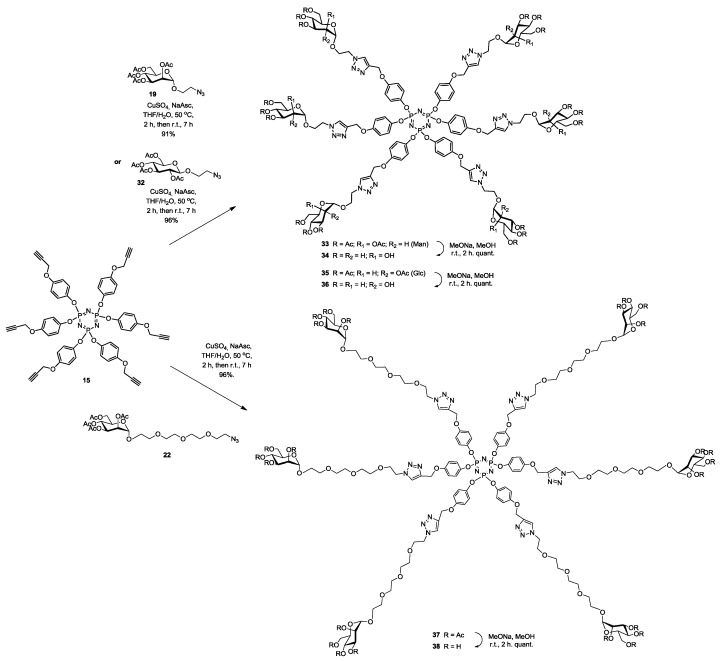
Synthesis of hexavalent phosphorus glycodendrimers **34**, **36** and **38** using click chemistry on hexapropagylated core **15** and azido sugars **19**, **32** and **22** respectively.

Multivalent binding interactions of glycodendrimers constitute a well-established strategy to enhanced binding efficacies toward their cognate lectins. We chose to prepare several dendrimers harboring from 2 to 18 mannopyranoside moieties to be evaluated as inhibitors against *E. coli* biofilm formation by using the highly efficient click chemistry. Azide-ending mannoside **22** was anchored to bis-propargylated aromatic scaffold **7** (Scheme 5), tris-propargylated aromatic scaffold **2**, tetrapropargylated scaffold **10** (Scheme 6) and hexa-propargylated phosphorus core **15** (Scheme 7) using CuAAC click reaction to afford **23**, **28**, **30** and **37** in good yields (75%, 92%, 82% and 96%, respectively). Completion of the reaction was evidenced by proton and phosphorus NMR spectroscopy, wherein complete disappearance of the propargylic CH signal and the appearance of the newly formed characteristic singlet of the triazole moieties were observed. Additionally, mass spectral analysis showed the presence of the required molecular ion peak (experimental section). All glycodendrimers were fully deprotected using Zemplén conditions providing the desired glyco-ligands **24**, **29**, **31** and **38** quantitatively.

Hexameric mannosylated clusters possessing a short (**19**) and the long pegylated (**22**) azido mannosides were anchored onto hexapropargylated phosphorus core **15** using CuAAC click reaction to afford **34** and **38** in good yields of 91% and 96% respectively (Scheme 7). In order to compare the role played by the sugar moiety in the subsequent bioassays, the known short 2-azidoehyl β-d-glucopyranoside **32** [41] was also coupled to the above scaffold **15** under the same reaction conditions to provide **34** also in an excellent 96% yield. Completion of the reactions was again readily evidenced through their respective proton and phosphorus NMR spectroscopy, wherein complete disappearance of the propargylic CH signal and the appearance of the newly formed characteristic singlet of the triazole moieties are showed. Additionally, mass spectral analysis showed the presence of the required molecular ion peak (experimental section). All clusters were fully deprotected using Zemplén reaction (NaOMe, MeOH) to provide the desired glycosylated hexamers **34**, **36** and **38** quantitatively.

**Scheme 8:**
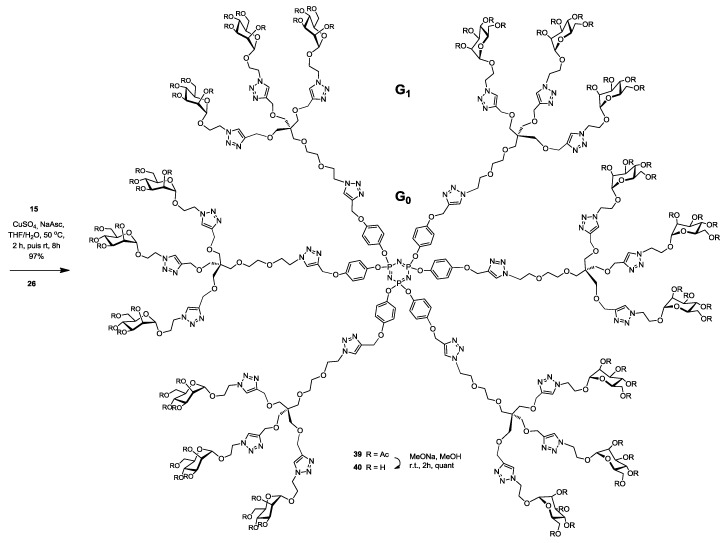
Synthesis of phosphorus glycodendrimer **40** having 18 α-d-mannopyranoside residues using click chemistry and built around a cyclotriphosphazene core and a pentaerythritol scaffold at the next generation level.

A G1 glycodendrimer **39** possessing 18 mannoside moieties was synthesized in good yield of 97%, by anchoring the azide-ending tri-valent mannosylated dendron **26** to the hexa-propargylated phosphorus core **15** using CuAAC click reaction. Completion of the reaction was evidenced by proton and phosphorus NMR spectroscopy, wherein complete disappearance of the propargylic CH signal δ 2.52 ppm and the appearance of the newly formed characteristic singlet of the triazole moieties δ 7.82 ppm, also the presence of one singlet phosphorus pick at δ 9.54 ppm fully support the homogeneity of the fully substituted cyclophosphazene core. Additionally, mass spectral analysis showed the presence of the required molecular ion peak (experimental section). Compound **39** was fully deprotected by using Zemplén reaction providing the desired G1 glycodendrimer **40** quantitatively (Scheme 8).

### 2.5. Dynamic Light Scattering (DLS) Studies

**Figure 1:**
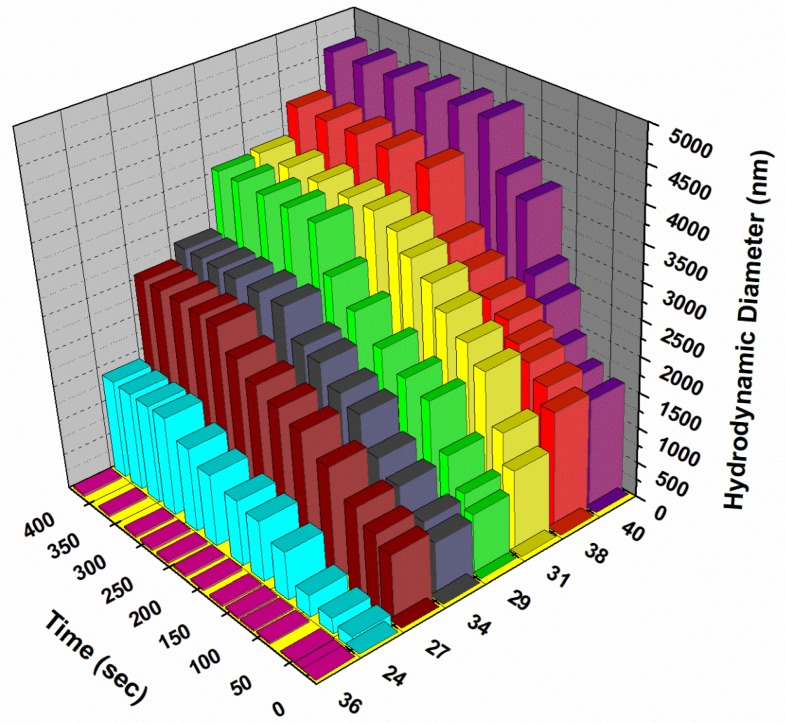
3D DLS plots of the cross-linking kinetics of ConA in the presence of mannosylated dendrimers **24**, **27**, **29**, **31**, **34**, **38** and **40** and the glucosylated dendrimer **36** taken as negative control.

The relative ability of the above glycodendrimers to react with a homotetrameric leguminous lectin such as Concanavalin A (ConA) from *Canavalia ensiformis* (jack bean) taken as a model and rapidly forming stable cross-linked lattices was monitored using dynamic light scattering (DLS). It was anticipated that the multivalent sugars at the peripheries of the dendrimers would facilitate the multidirectional interactions with its associated tetrameric protein as seen before with several other mannosylated structures [32,42,43,44,45,46,47]. Indeed, these multivalent glycodendrimer–protein complexes were shown to rapidly form large aggregates as a function of time. As seen in Figure 1, the complex resulting from the mannosylated dendrimers **24**, **27**, **29**, **31**, **38**, **40** and ConA resulted, within 1~3 min to nanometer sizes clusters having sizes ranging from 1400 to 4600 nm. When using the β-d-glucopyranoside dendrimer **36**, taken as negative control, no appreciable cross-linked lattice was observed. Clearly, the more highly dense mannodendrimer **40** (18 Man) reacted faster but also formed larger size aggregates than their hexameric cluster counterparts **34** and **38**. Interestingly, tetrameric mannoside **31** formed larger aggregates faster than trimer **29** but both smaller clusters plateaued at the same level of ~3000 nm size. Alternatively, tetramer **31** and hexamer **38** formed cross-linked lattices almost equally rapidly but the denser structure **38** formed larger aggregates at the end of the process. As anticipated, hexamer **34**, harboring short mannopyranoside aglycone, formed aggregates of smaller size (2530 nm) when compared with hexamer **38** containing the tetraethyleneglycol linker (4090 nm) (see Supplementary Materials). In addition, **38** reached its plateau faster (204 s) in comparison to **34** (230 s), indicating the higher adaptability of the longer spacer to form stable cross-linked lattices.

## 3. Materials and Methods

All reactions in organic medium were performed in standard oven dried glassware under an inert atmosphere of nitrogen using freshly distilled solvents stored over molecular sieves. Solvents were deoxygenated when necessary by bubbling nitrogen through the solution. All reagents were used as supplied without prior purification and obtained from Sigma-Aldrich Chemical Co. (Toronto, ON, Canada) Reactions were monitored by analytical thin-layer chromatography (TLC) using silica gel 60 F254 pre-coated plates (Merck, Darmstadt, Germany) and compounds were visualized by 254 nm light and/or by dipping into a mixture of sulfuric acid and methanol in water or into a mixture of KMin.O_4_ and K_2_CO_3_ in water followed by gentle warming with a heat-gun. Purifications were performed by flash column chromatography using silica gel from Canadian Life Science (60 Å, 40–63 μm) (Peterborough, ON, Canada) with the indicated eluent. ^1^H-NMR, ^13^C-NMR and ^31^P-NMR spectra were recorded at 300 and/or 600 MHz, 75 and/or 150 MHz and 122 and/or 243 MHz, respectively, on a Bruker spectrometer (300 MHz and 600 MHz) (Milton, ON, Canada) and Varian spectrometer (600 MHz) (Milton, ON, Canada). All NMR spectra were measured at 25 °C in indicated deuterated solvents. Proton and carbon chemical shifts (δ) are reported in ppm and coupling constants (J) are reported in Hertz (Hz). The resonance multiplicity in the ^1^H-NMR spectra are described as “s” (singlet), “d” (doublet), “t” (triplet) and “m” (multiplet) and broad resonances are indicated by “broad.” Residual protic solvent of CDCl_3_ (^1^H, 7.27 ppm; ^13^C, 77.0 ppm (central resonance of the triplet)), D_2_O (^1^H, 4.80 ppm and 30.9 ppm for CH_3_ of acetone for ^13^C spectra), MeOD (^1^H, 3.30 ppm and ^13^C, 49.0 ppm), 85% H_3_PO_4_ was used as an external reference for ^31^P-NMR. Two-dimensional homonuclear correlation ^1^H-^1^H COSY and ^1^H-^13^C HSQC experiments were used to confirm NMR peak assignments. Letters are used to NMR assignment. Accurate mass measurements (HRMS) were performed on a LC-MSD-TOF instrument from Agilent Technologies (Santa Clara, CA, USA) in positive electrospray mode. Either protonated molecular ions [M + nH]^n+^ or adducts [M + nX]^n+^ (X = Na, K, NH_4_) were used for empirical formula confirmation. Light Scattering-Multiangle Laser Light Scattering (LS-MALLS) detection with performances verified with polystyrene 100 kDa and 2000 kDa were used to determine the number-average molecular weight (Min.) and polydispersity index (M_W_/Min.). Calculations were performed with Zimm Plot (model). MALDI-TOF MS data were acquired on a Bruker Microflex LRF system (Bruker Daltonics, Billerica, MA, USA) equipped with a Compass 3.1 software platform. Acquisitions were performed in positive ion mode. Reflector mode was utilized for samples below 5 kDa and linear mode for samples above 5 kDa. Samples were dissolved in water (1 mg/mL) and mixed with 10 volumes of α-cyano-4-hydroxycinnamic acid matrix prepared at 10 mg/mL with 50% ACN/H_2_O 0.1% TFA. A volume of 2 μL was deposited on the target plate and dried. Representative acquisition parameters were: ion source 1, 19.5 kV; ion source 2, 18.05 kV; lens, 7.0 V; pulse ion extraction, 240 ns; detector, 1.905 kV. Approximately 200 laser shot/spectra were obtained with a 60 Hz N_2_-cartridge-laser set at 337 nm and the laser intensity adjusted according to the signal intensity. Sugar monomers were synthesized following the typical procedures found in literatures with a slight modification as describe bellow.

### Dynamic Light Scattering

Particle size distribution (DLS) was measured in water with the help of Zetasizer Nano S90 from Malvern Instruments. Crosslinking studies were carried out in 1 mol/L phosphate buffered saline (PBS) for the plant lectin Concavalin A (Sigma-Aldrich).

*General procedure for the Cu(I) azide-alkyne cycloaddition reaction (I) (CuAAc)* [28]. Solutions of alkyne (1 equiv.) and azide (1.5 equiv. per alkyne site) were prepared in minimum amount of THF. The resulting solutions were treated with an aqueous solution of CuSO_4_ (0.3 equiv. per alkyne site) and sodium ascorbate solution in THF (0.35 equiv. per alkyne site). Biphasic mixture was then stirred at 50 °C for 2 h then left at room temperature until completion of the reaction as judged by the complete conversion of the limiting regents (alkyne). After completion of the reaction, EtOAc was added, then Na_2_SO_4_ was added to quench the reaction by regenerating the CuSO_4_·5H_2_O. The mixture was allowed to stir for 3 min then filtered before the solvent was removed under reduced pressure and the resulting viscous oil was subjected to silica gel column chromatography using the appropriate eluent system (0–10% MeOH in CH_2_Cl_2_) to afford the triazole products.

*General procedure for the azide substitution (II)* [28]. To a solution of the appropriate bromo/tosyl derivatives (1 equiv.) in DMF was added NaN_3_ (1.5 equiv. per bromide). The reaction mixtures were stirred at 80 °C until completion of reaction as judged by TLC. The excess solvent was next removed under reduced pressure with heating at 60 °C until dryness. The residues were dissolved in EtOAc and washed successively with water and brine. The organic layer was separated and dried on Na_2_SO_4_. The residue was subjected to silica gel flash chromatography to afford the desired azido derivative.

*General Procedure for De-O-acetylation (III) (Zemplén reaction)* [28]. An acetylated glycocluster (0.1 mmol) was dissolved in dry MeOH (3 mL) and a solution of sodium methoxide (1 M in MeOH, 0.5 equiv.) was added. The reaction mixture was stirred at room temperature until the starting material disappeared. The solution was neutralized by the addition of a cationic ion-exchange resin (H^+^), filtered and washed with MeOH and then the solvent was removed in vacuo. The residue was lyophilized to yield quantitatively the fully deprotected glycoclusters.

*Synthesis of***2**. *Benzene-1,3,5-triol* (**1**) [48] (2 g, 15.86 mmol) was dissolved in DMF (10 mL) followed by the addition of K_2_CO_3_ (3 g, 14.87 mmol). The mixture was reflux for 30 min, propargyl bromide (8.5 mL, 79.3 mmol, 5 equiv.) was added dropwise, then stirred over night at room temperature. The residue was dissolved in dichloromethane (DCM) and washed with water and brine. The organic layer was dried over Na_2_SO_4_, filtered, then concentered under vacuum. The crude product was purified by silica gel flash chromatography using 0–20% EtOAc in Hexane as eluents to afford compound **2** as a white powder (2 g, 53%). *R*_f_ = 0.28 (EtOAc/Hexane, 1:4). ^1^H-NMR (300 MHz, CDCl_3_) δ 6.27 (s, 3H, k), 4.65 (d, *J* = 2.4 Hz, 6H, b), 2.53 (t, *J* = 2.4 Hz, 3H, c).

*Synthesis of compound***9**. To a solution of pentaerythritol **8** (2 g, 14.7 mmol) in dimethylsulfoxide (DMSO, 15 mL) were added an aqueous solution of NaOH (40 wt%, 10 mL). The solution was kept under magnetic stirring at room temperature for 30 min Propargyl bromide (97%, 12 mL, 135.18 mmol) was then added and the solution was kept at room temperature for an additional 24 h. Ethyl ether was added to the reaction mixture then washed with water. The organic layer was dried over Na_2_SO_4_. The crude product was purified by silica gel flash chromatography using 0–20% EtOAc in Hexane as eluents to afford compound **9** as a colorless oil (2.2 g, 60%). *R*_f_ = 0.25 (EtOAc/Hexane, 1:4). Compound **10** was obtained as a white powder (1.2 g, 28%). *R*_f_ = 0.35 (EtOAc/Hexane, 1:4). Tetrapropargylpentaerythritol **10** [49]. ^1^H-NMR (300 MHz, CDCl_3_) δ 4.08 (d, *J* = 2.3 Hz, 8H, OC*H*_2_CCH), 3.48 (s, 8H, C*H*_2_OCH_2_CCH), 2.42 (d, *J* = 2.2 Hz, 4H, OCH_2_CC*H*).

*Tripropargylpentaerythritol***9** [49]. ^1^H-NMR (300 MHz, CDCl_3_) δ 4.13 (d, *J* = 2.4 Hz, 6H, OC*H*_2_CCH), 3.69 (s, 2H, C*H*_2_OH), 3.56 (s, 6H, CH_2_OC*H*_2_CCH), 2.42 (t, *J* = 2.4 Hz, 3H, CC*H*).

*Synthesis of compound***11**. A solution of pentaerythritol propargyl ether (**9**) (2 g, 7.9 mmol, 1 equiv.), Bu_3_NHSO_4_ (5 g, 14.7 mmol, 1.8 equiv.), Bis(2-chloroethyl) ether (22 mL, 187.61 mmol) in NaOH 50% (30 mL), was stirred at room temperature for 24 h. DCM was added to the reaction mixture and the organic layer was washed successfully with water and brine, then dried over anhydrous sodium sulfate. The crude product was purified by silica gel flash chromatography using 0–20% EtOAc in Hexane as eluents to afford compound **11** as a colorless oil (2.1 g, 73%). *R*_f_ = 0.25 (EtOAc/Hexane, 1:4). ^1^H-NMR (300 MHz, CDCl_3_) δ 4.11 (d, *J* = 2.4 Hz, 6H, *O*C*H*_2_CCH), 3.76 (dd, *J* = 8.8, 3.2 Hz, 2H, C*H*_2_*-Cl*), 3.67–3.56 (m, 6H, OC*H*_2_*-*C*H*_2_), 3.52 (s, 6H, C*H*_2_*O*C*H*_2_CC*H*), 3.46 (s, 2H, C*H*_2_*O*CH_2_), 2.40 (t, *J* = 2.4 Hz, 3H, CC*H*).

*Monopropagyloxyphenol* (**13**) [50]. Hydroquinone (10.28 g, 93.37 mmol) was dissolved in acetone (150 mL) followed by the addition of K_2_CO_3_ (15.42 g, 111.58 mmol). The mixture was reflux for 30 min, propargyl bromide (10.28 mL, 93.50 mmol, 0.98 equiv.) was added dropwise, then stirred over night at room temperature. Residue was dissolved in DCM (100 mL) and washed with water (3 × 50 mL) and brine (3 × 50 mL). The organic layer was dried over Na_2_SO_4_, filtered and concentered under vacuum. The crude product was purified by silica gel flash chromatography using 0–30% EtOAc in Hexane as eluents to afford the mono substituted propargyl hydroquinone **13** as a brownish powder (3.11 g, 20.96 mmol, 43%). *R*_f_ = 0.35 (EtOAc/Hexane, 3:7). ^1^H-NMR (300 MHz, CDCl_3_) δ 6.85 (d, *J* = 9.1 Hz, 2H, aromatic), 6.78 (d, *J* = 9.1 Hz, 2H, aromatic), 4.66 (d, *J* = 1.4 Hz, 2H, C*H*_2_), 2.53 (t, *J* = 2.4 Hz, 1H, *≡*C*H*).

*Synthesis of***15** [50,51,52,53,54,55]. Cs_2_CO_3_ (3.4 g, 10.4 mmol) is added into a reaction mixture of 4-(prop-2-yn-1-yloxy)phenol (**13**) (6.7 mmol) and N_3_P_3_Cl_6_ (**14**) (0.6 mmol) in THF (20 mL). The mixture was heated at 40 °C for 17 h, then filtered on Celite and concentrated. The pure **15** was isolated as a white crystal yielding 98% after crystallization in EtOH. ^1^H-NMR (300 MHz, CDCl_3_) δ 6.87 (d, *J* = 9.65 Hz, 6H, OC_6_*H*_4_O), δ 6.80 (d, *J* = 9.65 Hz, 6H, OC_6_*H*_4_O), 4.65 (d, *J* = 2.4 Hz, 6H, OC*H*_2_), 2.52 (t, *J* = 2.4 Hz, 3H, C*H*). ^31^P-NMR (122 MHz, CDCl_3_) δ 9.82 (s).

*1,2,3,4,6-Penta-O-acetyl-α/β-d-mannopyranose* (**17**). 1,2,3,4,6-penta-*O*-acetyl-α/β-d-mannopyranose was prepared according to the procedure reported [52] with a slight modification. d-Mannose **16** (3 g, 16.6 mmol), pyridine (40 mL) and acetic anhydride (32 mL, 333 mmol) were stirred at room temperature. After stirring for 12 h, the reaction mixture was diluted with ice-water and extracted with DCM. The combined organic layer was washed with 1 M aqueous HCl, saturated sodium bicarbonate solution (NaHCO_3_), H_2_O and Brine. The organic layer was dried over Na_2_SO_4_ and the solvent was removed under reduced pressure. The product **17** was obtained as colorless syrup (6 g, 15.4 mmol, 90%) which was a mixture of α and β anomers with a ratio of 3:1. This product was used in the next synthetic step without any further purification. ^1^H-NMR (300 MHz, CDCl_3_) δ 6.09 (d, *J* = 1.8 Hz, 3H, *H*-1a), 5.86 (d, *J* = 1.1 Hz, 1H, *H*-1b), 5.49 (dd, *J* = 3.3, 1.1 Hz, 1H), 5.38–5.33 (m, 6H), 5.32 (d, *J* = 2.8 Hz, 1H), 5.26 (t, *J* = 2.0 Hz, 3H), 5.13 (dd, *J* = 10.0, 3.3 Hz, 1H), 4.30 (ddd, *J* = 12.4, 7.5, 5.1 Hz, 5H), 4.18–4.02 (m, 8H), 3.80 (ddd, *J* = 9.8, 5.3, 2.4 Hz, 1H), 2.22 (s, 3H), 2.18 (s, 9H), 2.17 (s, 9H), 2.10 (s, 3H), 2.09 (s, 12H), 2.05 (s, 12H), 2.01 (s, 12H).

*2-Bromoethyl 2,3,4,6-tetra-O-acetyl-α-d-manno-pyranoside* (**18**) [27,51]. Compound **17** (5.37 g, 13.8 mmol) and 2-bromoethanol (0.98 mL, 13.8 mmol) were dissolved in DCM (50 mL). Then, boron trifluoride etherate (5.8 mL, 47.2 mmol) was added to the solution and stirred under a nitrogen atmosphere for 3 h and monitored by TLC (EtOAc/Hexane, 1:1). After addition of DCM (100 mL), the reaction mixture was neutralized by adding saturated sodium bicarbonate solution (100 mL) and the resulting solution was washed with water (2 × 200 mL). The combined organic layers were dried over Na_2_SO_4_, filtered and concentrated to dryness under reduced pressure. The resulting oil was then purified using silica gel chromatography (EtOAc/Hexane, 1:1). The relevant fractions were collected, combined and concentrated to dryness under reduced pressure to yield **18** as a colorless powder (2.2 g, 40%). ^1^H-NMR (300 MHz, CDCl_3_) δ 5.39–5.18 (m, 4H, H-2, 3 and 4), 4.85 (d, *J* = 1.6 Hz, 1H, *H*-1), 4.27 (dd, *J* = 12.2, 5.3 Hz, 1H, *H*-6), 4.16–3.98 (m, 2H, *H*-6, *H*-5), 3.85 (ddd, *J* = 10.6, 6.6, 4.0 Hz, 1H, j), 3.72–3.60 (m, 1H, j′), 3.54–3.34 (m, 2H, C*H*_2_Br), 2.12 (s, 3H, COC*H*_3_), 2.08 (s, 3H, COC*H*_3_), 2.03 (s, 3H, COC*H*_3_), 1.97 (s, 3H, COC*H*_3_).

*2-Azidoethyl 2,3,4,6-tetra-O-acetyl-α-d-manno-pyranoside* (**19**) [27,51]. Compound **18** (1.40 g, 3.0 mmol) and sodium azide (1.00 g, 15.4 mmol) were dissolved in anhydrous DMF (30 mL) and stirred at 80 °C for 5 h. The reaction mixture was filtered and concentrated to dryness under reduced pressure and further processed as given in general procedure (*II*) to afford compound **19** (0.75 g, 60%) as white powder. ^1^H-NMR (300 MHz, CDCl_3_) δ 5.48–5.11 (m, 3H, *H*-2, 3 and 4), 4.87 (d, *J* = 1.6 Hz, 1H, *H*-1), 4.29 (dd, *J* = 12.3, 5.3 Hz, 1H, *H*-6), 4.20–3.99 (m, 2H, *H*-6′ and 5), 3.87 (m, 1H, j), 3.76–3.60 (m, 1H, j′), 3.56–3.36 (m, 2H, C*H*_2_N_3_), 2.16 (s, 3H, COC*H*_3_), 2.11 (s, 3H, COC*H*_3_), 2.06–2.03 (s, 3H, COC*H*_3_), 2.00 (s, 3H, COC*H*_3_).

*Monotosylated tetraethylene glycol* (**20**) [53]. To a solution of tetraethylene glycol (26.3 g, 135.4 mmol, 10 equiv.) in THF (60 mL) was added a solution of sodium hydroxide (1.79 g, 44.7 mmol, 3.3 equiv.) dissolved in water (5 mL). The mixture was cooled to 0 °C and toluensulfonyl chloride (2.57 g, 16.54 mmol, 1 equiv.) in THF (5 mL) was added dropwise. The reaction was stirred at 0 °C for 2 h. The solution was poured into water and the aqueous layer was extracted with dichloromethane. The organic layers were washed with water, dried over Na_2_SO_4_, filtered and concentrated under reduced pressure to yield **20** as a colorless oil (4.69 g, 97%) yield based on the *p*-toluenesulfomyl chloride. *R*_f_ = 0.25 (in 100% EtOAc). ^1^H-NMR (300 MHz, CDCl_3_) δ 7.79 (d, *J* = 8.0 Hz, 2H, b), 7.33 (d, *J* = 8.0 Hz, 2H, d), 4.16 (t, *J* = 4.7 Hz, 2H, a), 3.75–3.55 (m, 14H, e), 2.44 (s, 3H, c).

*2-(2-{2-[2-(2-Tosyloxy-ethoxy)-ethoxy]-ethoxy}-ethyl) 2,3,4,6-tetra-O-acetyl-α-d-mannopyranoside* (**21**) [53]. Into a solution of pentaacetate mannose **17** (1.68 g, 4.31 mmol, 1 equiv.) in anhydrous CH_2_Cl_2_ (20 mL) was added boron trifluoride etherate (1.23 mL, 9.93 mmol, 2.3 equiv.) at room temperature under nitrogen atmosphere. The solution was stirred for 4 h before compound **20** (3.76 g, 10.79 mmol, 2.5 equiv.) was added. Glycosylation was completed after stirring at 40 °C overnight. The crude product was washed with NaHCO_3_ sat, dried over Na_2_SO_4_ and concentrated under reduced pressure. Further purification was processed on silica gel flash chromatography using 0–20% EtOAc in Hexane as eluents to afford compound **21** as a colorless oil (1.8 g, 60%). *R*_f_ = 0.35 (EtOAc/Hexane, 1:4). ^1^H-NMR (300 MHz, CDCl_3_) δ 7.80 (d, *J* = 8.3 Hz, 2H, aromatic), 7.34 (d, *J* = 8.0 Hz, 2H, aromatic), 5.38–5.25 (m, 3H, *H*-3, 4 and 2), 4.87 (d, *J* = 1.6 Hz, 1H, *H*-1), 4.36–4.19 (m, 2H, *H*-6), 4.18–4.01 (m, 3H, c and *H*-5), 3.85–3.75 (m, 1H, j’), 3.73–3.56 (m, 13H, j, i, d, g, f, e), 2.45 (s, 3H, C*H*_3_), 2.16 (d, 3H, COC*H*_3_), 2.09 (s, 3H, COC*H*_3_), 2.03 (s, 3H, COC*H*_3_), 1.99 (s, 3H, COC*H*_3_).

*2-(2-{2-[2-(2-Azido-ethoxy)-ethoxy]-ethoxy}-ethyl) 2,3,4,6-tetra-O-acetyl-α-d-mannopyranoside* (**22**) [53]. To a solution of **21** (520 mg, 0.766 mmol, 1 equiv.) in dry DMF (15 mL) under a nitrogen atmosphere were added sodium azide (996 mg, 15.3 mmol, 20 equiv.). After stirring at 80 °C for 2 h, the solution was diluted in EtOAc and further processed as given in general procedure (*II*) to afford compound **22** as a colorless oil (380 mg, 90%), *R*_f_ = 0.4 (EtOAc/Hexane, 1:4). ^1^H-NMR (300 MHz, CDCl_3_) δ 5.43–5.24 (m, 3H, *H*-3, 4 and 2), 4.87 (d, *J* = 1.6 Hz, 1H, *H*-1), 4.27 (td, *J* = 12.6, 5.0 Hz, 1H, *H*-6), 4.14–4.03 (m, 2H, *H*-6′ and 5), 3.86–3.76 (m, 1H, j′), 3.71–3.64 (m, 13H, j, i, d, g, e, h, f), 3.39 (t, *J* = 5.1 Hz, 2H, *CH*_2_*-N*_3_), 2.15 (s, 3H, *C*O*C*H_3_), 2.10 (s, 3H, *C*O*C*H_3_), 2.04 (s, 3H, *C*O*C*H_3_), 1.99 (s, 3H, *C*O*C*H_3_).

*Synthesis of***23**. Into a solution of bispropargylated core (**7**) (30 mg, 0.13 mmol, 1.0 equiv.) in a mixture of THF/H_2_O (4 mL, 3:1, *v*/*v*), was added 2-(2-{2-[2-(2-Azido-ethoxy)-ethoxy]-ethoxy}-ethyl) 2,3,4,6-tetra-*O*-acetyl-α-d-mannopyranoside (**22**) (220 mg, 0.39 mmol, 3 equiv.), Na-ascorbate (18 mg, 0.09 mmol, 0.7 equiv.) and CuSO_4_·5H_2_O (19 mg, 0.08 mmol, 0.6 equiv.). The mixture was stirred at 50 °C for 2 h, then at room temperature for 5 h. Upon completion of the reaction, EtOAc was added to the reaction mixture and further processed as given in general procedure (*I*) to afford compound **23** (136 mg, 75%) as a white powder; *R*_f_ = 0.35 (with 5% MeOH in CH_2_Cl_2_ as eluents). ^1^H-NMR (600 MHz, CDCl_3_) δ 7.74 (s, 2H, *H*-triazole), 6.77 (s, 1H, *H**_P_***-aromatic), 6.74 (s, 2H, *H**_O_***-aromatic), 5.30 (dd, *J* = 10.0, 3.4 Hz, 2H, *H*-3), 5.25 (d, *J* = 9.9 Hz, 2H, *H*-4), 5.23–5.20 (m, 2H, *H*-2), 4.82 (s, 2H, *H*-1), 4.61 (s, 4H, b), 4.52–4.49 (m, 4H, c), 4.47 (s, 4H, m), 4.24 (dd, *J* = 12.2, 4.8 Hz, 2H, *H*-6), 4.05 (dd, *J* = 12.3, 4.1 Hz, 2H, *H*-6′), 4.03–3.99 (m, 2H, *H*-5), 3.83 (t, *J* = 4.9 Hz, 4H, d), 3.77–3.73 (m, 2H, j), 3.61 (m, 2H, j′), 3.60 (s, 4H, i), 3.58 (s, 16H, g, f, e, h), 2.11 (s, 6H, COC*H*_3_), 2.05 (s, 6H, COC*H*_3_, 1.99 (s, 6H, COC*H*_3_), 1.94 (s, 6H, COC*H*_3_). ^13^C-NMR (150 MHz, CDCl_3_) δ 170.6 (*C*=O), 169.9 (*C*=O), 169.8 (*C*=O), 169.6 (*C*=O), 156.8 (*C*OH), 144.7 (*C*-triazole), 139.5 (*C*_m_-aromatic), 123.8 (*C*H-triazole), 118.4 (*C*_p_-aromatic), 113.9 (*C**_O_***-aromatic), 97.5 (*C*-1), 71.9 (*C_m_*), 70.5 (*C_d_*), 70.4 (*C_g_*), 70.3 (*C_f_*), 69.8 (*C_e_*), 69.4 (*C_h_*), 69.4 (*C_j_*), 69.3 (*C*-2), 68.9 (*C*-3), 68.2 (*C*-4), 67.2 (*C*-5), 65.9 (*C_i_*), 63.4 (*C_b_*), 62.2 (*C*-6), 50.1 (*C_c_*), 20.8 (*C*O*C*H_3_), 20.6 (*C*O*C*H_3_), 20.6 (*C*O*C*H_3_). ESI^+^-HRMS: [M + Na]^+^ calcd for C_56_H_86_N_6_NaO_29_, 1329.5331; found: 1329.5395.

*Synthesis of***24**. Compound **23** (50 mg, 0.036 mmol) and sodium methoxide (16 μL from 1 M solution in MeOH) in 3 mL of methanol were stirred for 4 h and the mixture was treated following the general procedure (*III*) described above. Deprotected compound **24** (38 mg, quant.) was obtained as a colorless solid. ^1^H-NMR (600 MHz, D_2_O) δ 8.04 (s, 2H), 6.89 (s, 1H), 6.82 (s, 2H), 4.84 (s, 2H), 4.70 (s, 4H), 4.66–4.59 (m, 4H), 4.57 (s, 4H), 3.98–3.94 (m, 4H), 3.93–3.80 (m, 4H), 3.80–3.72 (m, 4H), 3.67–3.60 (m, 12H), 3.60–3.55 (m, 8H), 3.55–3.52 (m, 4H). ^13^C-NMR (150 MHz, D_2_O) δ 215.4, 155.9, 143.9, 139.3, 125.5, 120.1, 114.7, 99.8, 72.7, 71.7, 70.5, 69.9, 69.7, 69.6, 69.5, 69.4, 69.4, 68.7, 66.7, 66.3, 62.4, 60.9, 49.9, 30.2. ESI^+^-HRMS: [M + Na] ^+^ calcd for C_40_H_70_N_6_NaO_21_, 993.4486; found: 993.4545.

*Synthesis of***25**. Into a solution of tripropargyl pentaerythritol core (**11**) (125 mg, 0.35 mmol, 1.0 equiv.) in a mixture of THF/H_2_O (4 mL, 3:1, *v*/*v*), was added 2-azidoethyl 2,3,4,6-tetra-*O*-acetyl-α-d-mannopyranoside (**19**) (745 mg, 1.42 mmol, 5.1 equiv.), Na-ascorbate (70 mg, 0.29 mmol, 1.1 equiv.) and CuSO_4_·5H_2_O (78 mg, 0.31 mmol, 0.9 equiv.). The mixture was stirred at 50 °C for 2 h, then at room temperature for 5 h. Upon completion of the reaction, EtOAc was added to the reaction mixture and further processed as given in general procedure (*I*) to afford compound **25** (480 mg, 82%) as a white powder; *R*_f_ = 0.3 (with 5% MeOH in CH_2_Cl_2_ as eluents). ^1^H-NMR (300 MHz, CDCl_3_) δ 7.70 (s, 3H, *H**_B_***-triazole), 5.31–5.19 (m, 9H, *H*-3, 4 and 2), 4.81 (d, *J* = 1.3 Hz, 3H, *H*-1), 4.61 (m, 6 H, i), 4.59 (s, 6H, h), 4.21 (dd, *J* = 12.3, 5.1 Hz, 3H, *H*-6), 4.13 (dt, *J* = 10.4, 4.9 Hz, 3H, j′), 4.04 (dd, *J* = 12.3, 2.3 Hz, 3H, *H*-6′), 3.91 (dt, *J* = 10.6, 5.2 Hz, 3H, j), 3.74 (m, 2H, d), 3.61 (m, 7 H, *H*-5, f and C*H*_2_*-*Cl), 3.57–3.53 (m, 2H, e), 3.49 (s, 6H, g′), 3.44 (s, 2H, g), 2.13 (s, 9H, COC*H*_3_), 2.09 (s, 9H, COC*H*_3_), 2.04 (s, 9H, COC*H*_3_), 1.99 (s, 9H, COC*H*_3_).

*Synthesis of***26**. To a solution of compound **25** (480 mg, 0.29 mmol, 1.0 equiv.) in dry DMF (2 mL) under a nitrogen atmosphere were added sodium azide (186 mg, 2.9 mmol, 10 equiv.). After stirring at 80 °C for 2 h, the solution was diluted in EtOAc and further processed as given in general procedure (*II*) to afford compound **26** as a white powder (430 mg, 90%). ^1^H-NMR (300 MHz, CDCl_3_) δ 7.65 (s, 3H, *H**_B_***-triazole), 5.28–5.09 (m, 9H, *H*-3, 4 and 2), 4.76 (d, *J* = 1.1 Hz, 3H, *H*-1), 4.6–4.57 (m, 6H, i), 4.53 (s, 6H, h), 4.15 (dd, *J* = 12.4, 5.1 Hz, 3H, *H*-6), 4.07 (dt, *J* = 10.5, 5.7 Hz, 3H, j’), 3.98 (dd, *J* = 12.3, 2.3 Hz, 3H, *H*-6′), 3.86 (dt, *J* = 10.4, 5.1 Hz, 3H, j), 3.63–3.59 (m, 2H, d), 3.56–3.53 (m, 5H, H-5, f), 3.49 (m, 2H, e), 3.44 (s, 6H, g′), 3.39 (s, 2H, g), 3.33–3.27 (m, 2H, c, C*H*_2_*-*N_3_), 2.07 (s, 9H, COC*H*_3_), 2.03 (s, 9H, COC*H*_3_), 1.98 (s, 9H, COC*H*_3_), 1.94 (s, 9H, COC*H*_3_). ^13^C-NMR (75 MHz, CDCl_3_) δ 170.6 (*C*=O), 170.0 (*C*=O), 169.6 (*C*=O), 169.1 (*C*=O), 145.6 (*C*-triazole), 123.7 (*C*H***_B_***-triazole), 97.6 (*C*-1), 77.2 (*C_d_*), 71.0 (*C_f_*), 70.3 (*C_e_*), 70.0 (*C_g_*), 69.2 (*C*-2), 69.1 (*C*-3), 68.9 (*C*-4), 66.3 (*C*-5), 65.7 (*C_j_*), 64.8 (*C_h_*), 62.2 (*C*-6), 51.8 (*C_i_*), 50.8 (*C_c_*), 49.5 (*C_q_*), 20.8 (*COC*H_3_), 20.7 (*COC*H_3_).

*Synthesis of***27**. Compound **26** (200 mg, 0.02 mmol) and sodium methoxide (16 μL from 1 M solution in MeOH) in 3 mL of methanol were stirred for 4 h and the mixture was treated following the general procedure (*III*) described above. Deprotected compound **27** (150 mg, quant.) was obtained as a white powder. ^1^H-NMR (300 MHz, MeOD) δ 8.00 (s, 3H), 4.69–4.60 (m, 9H), 4.55 (s, 6H), 4.11 (dt, *J* = 10.3, 4.9 Hz, 3H), 3.88 (dt, *J* = 5.8, 4.6 Hz, 3H), 3.81–3.71 (m, 6H), 3.65 (ddd, *J* = 18.4, 7.4, 5.7 Hz, 18H), 3.58–3.49 (m, 4H), 3.46 (s, 6H), 3.40 (dd, *J* = 9.5, 4.3 Hz, 6H), 3.21 (dd, *J* = 7.6, 5.1 Hz, 4H). ^13^C-NMR (75 MHz, MeOD) δ 146.08, 126.31, 101.33, 79.41, 74.58, 72.26, 72.06, 71.69, 71.35, 70.99, 70.1, 68.1, 66.9, 65.2, 62.4, 51.8, 51.4, 50.1, 49.9, 49.6, 49.3, 49.0, 49.0, 48.7, 48.4, 46.5, 31.3, 24.6. ESI^+^-HRMS: [M + 2H]^2+^ calcd for C_42_H_72_N_12_O_23_, 556.2418; found: 556.2470.

*Synthesis of***28**. Into a solution of trispropargylated core (**2**) (25 mg, 0.104 mmol, 1.0 equiv.) in a mixture of THF/H_2_O (4 mL, 3:1, *v*/*v*), was added 2-(2-{2-[2-(2-Azido-ethoxy)-ethoxy]-ethoxy}-ethyl) 2,3,4,6-tetra-*O*-acetyl-α-d-mannopyranoside (**22**) (281 mg, 0.5 mmol, 5 equiv.), Na-ascorbate (22 mg, 0.11 mmol, 0.5 equiv.) and CuSO_4_·5H_2_O (23 mg, 0.09 mmol, 0.9 equiv.). The mixture was stirred at 50 °C for 2 h, then at room temperature for 5 h. Upon completion of the reaction, EtOAc was added to the reaction mixture and further processed as given in general procedure (*I*) to afford compound **28** (188 mg, 92%) as a white powder; *R*_f_ = 0.32 (with 5% MeOH in CH_2_Cl_2_ as eluents). ^1^H-NMR (600 MHz, CDCl_3_) δ 7.83 (s, 3H, *H*-triazole), 6.28 (s, 3H, k) 5.32 (dd, *J* = 10.0, 2.8 Hz, 3H, *H*-3), 5.27 (t, *J* = 10.0 Hz, 3H, *H*-4), 5.25 (dd, *J* = 3.0, 1.7 Hz, 3H, *H*-2), 5.12 (s, 6H, b), 4.85 (d, *J* = 1.4 Hz, 3H, *H*-1), 4.55 (t, *J* = 4.9 Hz, 6H, c), 4.27 (dd, *J* = 12.4, 5.1 Hz, 3H, *H*-6), 4.07 (d, *J* = 12.8 Hz, 3H, *H*-6′), 4.03 (dd, *J* = 5.7, 3.5 Hz, 3H, *H*-5), 3.89 (t, *J* = 4.9 Hz, 6H, d), 3.82–3.73 (m, 3H, j), 3.65 (m, 3H, j′), 3.63 (s, 6H, i), 3.61 (s, 24H, g, e, f, h), 2.13 (s, 9H, COC*H*_3_), 2.07 (s, 9H, COC*H*_3_), 2.01 (s, 9H, COC*H*_3_), 1.96 (s, 9H, COC*H*_3_). ^13^C-NMR (150 MHz, CDCl_3_) δ 170.8 (*C*=O), 170.1 (*C*=O), 170.0 (*C*=O), 169.8 (*C*=O), 160.3 (*C*OC_b_-aromatic), 143.7 (*C*-triazole), 124.2 (*C*H-triazole), 97.8 (*C*-1), 95.1 (*C_k_*-aromatic), 70.8 (C_d_), 70.7 (*C_g_*), 70.6 (*C_f_*), 70.1 (*C_e_*), 69.7 (*C_h_*), 69.6 (*C_j_*), 69.5 (*C*-2), 69.2 (*C*-3), 68.5 (*C*-4), 67.5 (*C*-5), 66.2 (*C_i_*), 62.5 (*C_b_*), 62.1 (*C*-6), 50.4 (*C_c_*), 21.0 (*C*O*C*H_3_), 20.9 (*C*O*C*H_3_), 20.8 (*C*O*C*H_3_), 20.8 (*C*O*C*H_3_). MALDI-TOF: [M + H]^+^ calcd for C_81_H_118_N_9_O_42_: 1888.737; found, 1888.789.

*Synthesis of***29**. Compound **28** (100 mg, 0.05 mmol) and sodium methoxide (16 μL from 1 M solution in MeOH) in 3 mL of methanol were stirred for 4 h and the mixture was treated following the general procedure (*III*) described above. Deprotected compound **29** (74 mg, quant.) was obtained as a white solid. ^1^H-NMR (600 MHz, MeOD) δ 8.16 (s, 3H), 6.37 (s, 3H), 5.19 (s, 6H), 4.65–4.63 (m, 8H), 3.95–3.92 (m, 4H), 3.88–3.87 (m, 2H), 3.82 (m, 10.0, 3.1 Hz, 4H), 3.77–3.71 (m, 4H), 3.68–3.54 (m, 23H). ^13^C-NMR (150 MHz, MeOD) δ 159.9, 143.3, 125.4, 100.1, 95.5, 72.9, 70.9, 70.3, 69.9, 69.8, 69.8, 68.9, 66.9, 66.4, 61.1, 50.2, 48.5, 47.9, 47.9, 47.8, 47.8, 47.8. MALDI-TOF: [M]^+^ calcd for C_57_H_92_N_9_O_30_: 1382.592; found, 1382.801.

*Synthesis of***30**. Into a solution of tetrapropargyl pentaerythritol core (**10**) (67 mg, 0.23 mmol, 1.0 equiv.) in a mixture of THF/H_2_O (4 mL, 3:1, *v*/*v*), was added 2-(2-{2-[2-(2-Azido-ethoxy)-ethoxy]-ethoxy}-ethyl) 2,3,4,6-tetra-*O*-acetyl-α-d-mannopyranoside (**22**) (790 mg, 1.4 mmol, 6 equiv.), Na-ascorbate (65 mg, 0.32 mmol, 1.4 equiv.) and CuSO_4_·5H_2_O (70 mg, 0.28 mmol, 1.2 equiv.). The mixture was stirred at 50 °C for 2 h, then at room temperature for 5 h. Upon completion of the reaction, EtOAc was added to the reaction mixture and further processed as given in general procedure (*I*) to afford compound **30** as a white powder (484 mg, 82%). *R*_f_ = 0.27 (with 5% MeOH in CH_2_Cl_2_ as eluents). ^1^H-NMR (600 MHz, CDCl_3_) δ 7.68 (s, 4H, *H*-triazole), 5.32 (dd, *J* = 10.0, 3.4 Hz, 4H, *H*-3), 5.26 (t, *J* = 10.0 Hz, 4H, *H*-2), 5.24 (dd, *J* = 3.2, 1.6 Hz, 4H, *H*-4), 4.85 (s, 4H, *H*-1), 4.52 (s, 8H, b), 4.51 (t, *J* = 5.5 Hz, 8H, c), 4.27 (dd, *J* = 12.2, 4.9 Hz, 4H, *H*-6), 4.07 (dd, *J* = 12.2, 2.2 Hz, 4H, *H*-6′), 4.04 (ddd, *J* = 9.8, 4.8, 2.3 Hz, 4H, *H*-5), 3.87 (t, *J* = 5.3 Hz, 8H, d), 3.81–3.75 (m, 4H, j), 3.66 (dd, *J* = 5.8, 3.7 Hz, 4H, j′), 3.64–3.63 (m, 8H, i), 3.62–3.58 (m, 32H, g, f, e, h), 3.45 (s, 8H, k), 2.13 (s, 12H, COC*H*_3_), 2.08 (s, 12H, COC*H*_3_), 2.01 (s, 12H, COC*H*_3_), 1.96 (s, 12H, COC*H*_3_). ^13^C-NMR (150 MHz, CDCl_3_) δ 170.7 (*C*=O), 170.0 (*C*=O), 169.9 (*C*=O), 169.7 (*C*=O), 145.1 (*C*-triazole), 123.6 (*C*H-triazole), 97.7 (*C*-1), 70.7 (C_d_), 70.6 (*C_g_*), 70.5 (C_f_), 69.9 (*C_e_*), 69.6 (C_h_), 69.5 (*C_j_*), 69.5 (C-2), 69.2 (C_q_), 69.1 (C-3), 68.4 (C-4), 67.4 (C-5), 66.1 (C_i_), 64.9 (C_b_), 62.4 (C-6), 50.1 (C_c_), 45.3 (C_k_), 20.9 (CO*C*H_3_), 20.8 (CO*C*H_3_), 20.7 (CO*C*H_3_), 20.7 (CO*C*H_3_). ESI^+^-HRMS: [M + 2H]^2+^ calcd for C_105_H_162_N_12_O_56_, 1244.5021; found: 1244.0172.

*Synthesis of***31**. Compound **30** (100 mg, 0.038 mmol) and sodium methoxide (16 μL from 1 M solution in MeOH) in 3 mL of methanol were stirred for 4 h and the mixture was treated following the general procedure (*III*) described above. Deprotected compound **31** (70 mg, quant.) was obtained as a colorless solid. ^1^H-NMR (600 MHz, D_2_O) δ 8.01 (s, 4H), 4.87 (s, 4H), 4.60 (t, *J* = 4.9 Hz, 8H), 4.52 (s, 8H), 3.95 (d, *J* = 4.8 Hz, 12H), 3.90–3.86 (m, 4H), 3.85 (dd, *J* = 7.4, 3.7 Hz, 4H), 3.81 (dd, *J* = 9.1, 3.2 Hz, 4H), 3.75 (dd, *J* = 12.2, 5.4 Hz, 4H), 3.72–3.65 (m, 12H), 3.65–3.60 (m, 24H), 3.60–3.57 (m, 16H), 3.40 (s, 8H). ^13^C-NMR (150 MHz, D_2_O) δ 144.1, 125.3, 99.9, 72.7, 70.5, 69.9, 69.9, 69.7, 69.6, 69.5, 69.5, 68.7, 68.1, 66.7, 66.3, 63.5, 60.9, 49.9, 44.6. ESI^+^-HRMS: [M + 2H]^2+^ calcd for C_73_H_130_N_12_O_40_, 907.4248; found: 907.4258.

*Synthesis of***33**. Into a solution of hexapropargylated core (**15**) (30 mg, 0.03 mmol, 1.0 equiv.) in a mixture of THF/H_2_O (4 mL, 3:1, *v*/*v*), was added **19** (152 mg, 0.29 mmol, 9.6 equiv.), Na-ascorbate (12 mg, 0.06 mmol, 1.98 equiv.) and CuSO_4_·5H_2_O (14 mg, 0.05 mmol, 1.8 equiv.). The mixture was stirred at 50 °C for 2 h, then at room temperature for 5 h. Upon completion of the reaction, EtOAc was added to the reaction mixture and further processed as given in general procedure (*I*) to afford compound **33** as a white powder (97 mg, 91%); *R*_f_ = 0.3 (with 5% MeOH in CH_2_Cl_2_ as eluents). ^1^H-NMR (600 MHz, CDCl_3_) δ 7.83 (s, 6H, *H*-triazole), 6.85 (d, *J* = 9.0 Hz, 12H, aromatic), 6.79 (d, *J* = 9.1 Hz, 12H, aromatic), 5.31–5.19 (m, 18H, H-2, 3 and 4), 5.16–5.08 (m, 12H, Ar*-*OC*H*_2_), 4.82 (s, 6H, *H*-1), 4.64–4.54 (m, 12H, Man-OC*H*_2_), 4.21 (dd, *J* = 12.3, 5.1 Hz, 6H, *C*H*H*′*N*), 4.16–4.09 (m, 6H, *H*-6), 4.04 (dd, *J* = 12.3, 2.2 Hz, 6H, *H*-6′), 3.90 (dt, *J* = 10.4, 5.1 Hz, 6H, C*H*H′N), 3.65–3.58 (m, 6H, *H*-5), 2.13 (s, 18H, COC*H*_3_), 2.09 (s, 18H, COC*H*_3_), 1.98 (s, 18H, COC*H*_3_), 1.97 (s, 18H, COC*H*_3_). ^13^C-NMR (151 MHz, CDCl_3_) δ 170.8 (C=O), 170.2 (C=O), 170.2 (C=O), 169.9 (C=O), 155.7 (CO-aromatic), 145.1 (C-triazole), 144.5 (C_p_-aromatic), 124.5 (CH-triazole), 122.2 (C_m_-aromatic), 115.7 (C_O_-aromatic), 97.9 (C-1), 77.2 (C-2), 69.6 (C-3), 69.5 (C-4), 69.3 (C-5), 66.6 (C_j_), 66.2 (C_b_), 62.7 (C-6), 50.0 (C_i_), 21.1 (CO*C*H_3_), 21.0 (CO*C*H_3_), 20.9 (CO*C*H_3_). ^31^P-NMR (243 MHz, CDCl_3_) δ 9.84. ESI^+^-HRMS: [M + 3H]^3+^ calcd for C_150_H_183_N_21_O_72_P_3_, 1174.34752; found: 1174.6753.

*Synthesis of***34**. Compound **33** (97 mg, 0.03 mmol) and sodium methoxide (16 μL from 1 M solution in MeOH) in 3 mL of methanol were stirred for 4 h and the mixture was treated following the general procedure (*III*) described above. Deprotected compound **34** (69 mg, quant.) was obtained as a white solid. ^1^H-NMR (600 MHz, D_2_O) δ 7.80 (s, 6H), 6.56 (s, 24H), 4.84 (s, 18H), 4.65 (s, 6H), 4.36 (s, 12H), 3.87 (s, 6H), 3.74 (s, 6H), 3.71–3.50 (m, 24H), 3.05 (d, *J* = 3.2 Hz, 6H). ^13^C-NMR (151 MHz, D_2_O) δ 154.9, 143.8, 143.1, 125.1, 121.6, 115.5, 99.5, 72.8, 70.5, 69.9, 66.4, 65.2, 61.3, 60.6, 49.8. ^31^P-NMR (243 MHz, CDCl_3_) δ 10.16. ESI^+^-HRMS: [M + 3H]^3+^ calcd for C_102_H_135_N_21_O_48_P_3_, 838.26603; found: 838.5901.

*Synthesis of***35**. Into a solution of hexapropargylated core (**15**) (50 mg, 0.049 mmol, 1.0 equiv.) in a mixture of THF/H_2_O (4 mL, 3:1, *v*/*v*), was added **32** (185 mg, 0.44 mmol, 6 equiv.), Na-ascorbate (20 mg, 0.1 mmol, 0.1 equiv.) and CuSO_4_·5H_2_O (22 mg, 0.28 mmol, 0.09 equiv.). The mixture was stirred at 50 °C for 2 h, then at room temperature for 5 h. Upon completion of the reaction, EtOAc was added to the reaction mixture and further processed as given in general procedure (*I*) to afford compound **35** as a white powder (171 mg, 96%); *R*_f_ = 0.2 (with 5% MeOH in CH_2_Cl_2_ as eluents). ^1^H-NMR (600 MHz, CDCl_3_) δ 7.74 (s, 6H, *H*-triazole), 6.92–6.74 (m, 24H, aromatic), 5.16 (t, *J* = 9.5 Hz, 6H, *H*-3), 5.11 (d, *J* = 6.3 Hz, 12H, b), 5.05 (t, *J* = 9.7 Hz, 6H, *H*-4), 4.98 (dd, *J* = 9.6, 8.0 Hz, 6H, *H*-2), 4.60 (dt, *J* = 14.4, 4.0 Hz, 6H, j), 4.53–4.47 (m, 12H, j′, *H*-1), 4.24 (dt, *J* = 8.4, 4.5 Hz, 12H, i, *H*-6), 4.13 (dd, *J* = 12.3, 2.2 Hz, 6H, *H*-6′), 3.93 (ddd, *J* = 10.6, 8.8, 3.5 Hz, 6H, i′), 3.70 (ddd, *J* = 10.0, 4.6, 2.4 Hz, 6H, *H*-5), 2.07 (s, 18H, COC*H*_3_), 2.01 (s, 18H, COC*H*_3_), 1.97 (s, 18H, COC*H*_3_), 1.92 (s, 18H, COC*H*_3_). ^13^C-NMR (150 MHz, CDCl_3_) δ 169.6 (C=O), 169.1 (C=O), 168.4 (C=O), 168.4 (C=O), 154.2 (CO-aromatic), 143.6 (C-triazole), 142.7 (C**_p_**-aromatic), 123.3 (CH-triazole), 120.9 (C**_m_**-aromatic), 114.3 (C**_O_**-aromatic), 99.5 (C-1), 71.5 (C-2), 70.9 (C-3), 69.9 (C-4), 67.2 (C-5), 66.7 (C_j_), 61.2 (C_b_), 60.7 (C-6), 48.9 (*C_i_*), 19.7 (CO*C*H_3_), 19.7 (CO*C*H_3_), 19.6 (CO*C*H_3_), 19.5 (CO*C*H_3_). ^31^P-NMR (243 MHz, CDCl_3_) δ 9.72. MALDI-TOF: [M]^+^ calcd for C_150_H_179_N_21_O_72_P_3_: 3519.020; found, 3519.489.

*Synthesis of***36**. Compound **35** (100 mg, 0.03 mmol) and sodium methoxide (16 μL from 1 M solution in MeOH) in 3 mL of methanol were stirred for 4 h and the mixture was treated following the general procedure (*III*) described above. Deprotected compound **36** (78 mg, quant.) was obtained as a white powder. ^1^H-NMR (600 MHz, MeOD) δ 8.05 (s, 6H), 6.70 (dd, *J* = 40.7, 9.1 Hz, 24H), 5.02 (s, 12H), 4.51 (t, *J* = 5.0 Hz, 12H), 4.26 (d, *J* = 7.9 Hz, 6H), 4.17–4.07 (m, 6H), 4.01–3.84 (m, 6H), 3.75 (dd, *J* = 12.1, 1.9 Hz, 6H), 3.55 (dd, *J* = 12.1, 5.8 Hz, 6H), 3.31 (t, *J* = 9.0 Hz, 6H), 3.27–3.22 (m, 6H), 3.18 (d, *J* = 9.6 Hz, 6H), 3.11 (dd, *J* = 9.2, 8.0 Hz, 6H). ^13^C-NMR (150 MHz, MeOD) δ 156.3, 145.2, 144.2, 126.5, 122.7, 116.6, 103.8, 77.3, 77.1, 74.3, 70.9, 68.9, 62.4, 62.1, 51.4, 49.4, 49.0, 24.2. ^31^P-NMR (243 MHz, MeOD) δ 10.25. MALDI-TOF: [M + H]^+^ calcd for C_102_H_132_N_21_O_48_P_3_: 2512.778; found, 2512.851.

*Synthesis of***37**. Into a solution of hexapropargylated core (**15**) (25 mg, 0.025 mmol, 1.0 equiv.) in a mixture of THF/H_2_O (4 mL, 3:1, *v*/*v*), was added compound **22** (140 mg, 0.25 mmol, 10 equiv.), Na-ascorbate (10 mg, 0.05 mmol, 2.1 equiv.) and CuSO_4_·5H_2_O (10.8 mg, 0.04 mmol, 1.8 equiv.). The mixture was stirred at 50 °C for 2 h, then at room temperature for 5 h. Upon completion of the reaction, EtOAc was added to the reaction mixture and further processed as given in general procedure (*I*) to afford compound **37** as a white powder (101 mg, 96%); *R*_f_ = 0.22 (with 4% MeOH in CH_2_Cl_2_ as eluents). ^1^H-NMR (600 MHz, CDCl_3_) δ 7.85 (s, 6H, *H*-triazole), 6.81 (dd, *J* = 29.7, 9.0 Hz, 24H, aromatic), 5.33 (dd, *J* = 10.0, 3.4 Hz, 6H, *H*-3), 5.28 (t, *J* = 10.0 Hz, 6H, *H*-2), 5.25 (dd, *J* = 3.2, 1.6 Hz, 6H, *H*-4), 5.13 (d, *J* = 7.6 Hz, 12H, b), 4.86 (s, 6H, *H*-1), 4.54 (t, *J* = 5.1 Hz, 12H, c), 4.28 (dd, *J* = 12.2, 4.9 Hz, 6H, *H*-6), 4.08 (dd, *J* = 12.3, 2.2 Hz, 6H, *H*-6′), 4.05 (ddd, *J* = 9.7, 4.8, 2.3 Hz, 6H, *H*-5), 3.88 (t, *J* = 5.2 Hz, 12H, d), 3.81–3.76 (m, 6H, j), 3.67–3.65 (m, 6H, j′), 3.63 (d, *J* = 3.7 Hz, 12H, i), 3.61 (d, *J* = 7.6 Hz, 48H, g, f, e, h), 2.14 (s, 18H, COC*H*_3_), 2.09 (s, 18H, COC*H*_3_), 2.02 (s, 18H, COC*H*_3_), 1.98 (s, 18H, COC*H*_3_). ^13^C-NMR (150 MHz, CDCl_3_) δ 170.7 (C=O), 170.0 (C=O), 169.9 (C=O), 169.7 (C=O), 155.3 (CO-aromatic), 144.6 (C-triazole), 143.6 (C**_p_**-aromatic), 124.2 (CH-triazole), 121.8 (C**_m_**-aromatic), 115.3 (C**_O_**-aromatic), 97.7 (C-1), 70.7 (C_d_), 70.6 (C_g_), 70.5 (C_f_), 69.9 (C_e_), 69.6 (C_h_), 69.5 (C_j_), 69.4 (C-2), 69.1 (C-3), 68.4 (C-4), 67.4 (C-5), 66.1 (C_i_), 62.4 (C_b_), 62.3 (C-6), 50.2 (C_c_), 20.9 (CO*C*H_3_), 20.8 (CO*C*H_3_), 20.7 (CO*C*H_3_), 20.7 (CO*C*H_3_). ^31^P-NMR (243 MHz, CDCl_3_) δ 9.68. ESI^+^-HRMS: [M + 2H]^2+^ calcd for C_186_H_254_N_21_O_90_P_3_, 2158.2604; found: 2158.2588.

*Synthesis of***38**. Compound **37** (100 mg, 0.023 mmol) and sodium methoxide (16 μL from 1 M solution in MeOH) in 3 mL of methanol were stirred for 4 h and the mixture was treated following the general procedure (*III*) described above. Deprotected compound **38** (77 mg, quant.) was obtained as a white solid. ^1^H-NMR (300 MHz, D_2_O) δ 7.96 (s, 6H, *H*-triazole), 6.71 (s, 24H, aromatic), 4.99 (s, 12H, b), 4.82 (s, 6H, *H*-1), 4.49 (s, 12H, c), 3.93–3.75 (m, 36H, *H*-2,3,4,5,6 and 6′), 3.74–3.38 (m, 84H, d, e, f, g, h, i, j). ^31^P-NMR (122 MHz, D_2_O) δ 10.11. ESI^+^-HRMS: [M + 2H]^2+^ calcd for C_138_H_206_N_21_O_66_P_3_, 1653.6321; found: 1653.6342.

*Synthesis of***39**. Into a solution of hexapropargylated core (**15**) (26 mg, 0.026 mmol, 1.0 equiv.) in a mixture of THF/H_2_O (4 mL, 3:1, *v*/*v*), was added (**26**) (400 mg, 0.24 mmol, 9.6 equiv.), Na-ascorbate (11 mg, 0.06 mmol, 2.1 equiv.) and CuSO_4_·5H_2_O (12 mg, 0.05 mmol, 1.8 equiv.). The mixture was stirred at 50 °C for 2 h, then at room temperature for 5 h. Upon completion of the reaction, EtOAc was added to the reaction mixture and further processed as given in general procedure (*I*) to afford compound **39** as a white powder (272 mg, 97%); *R*_f_ = 0.2 (with 5% MeOH in CH_2_Cl_2_ as eluents). ^1^H-NMR (600 MHz, CDCl_3_) δ 7.82 (s, 6H, H_A_-triazole), 7.69 (s, 18H, H_B_-triazole), 6.82 (dd, *J* = 32.8, 8.8 Hz, 24H, aromatic), 5.22 (t, *J* = 10.0 Hz, 18H, *H*-3), 5.19 (d, *J* = 3.2 Hz, 12H, *H*-4), 5.17 (s, 12H, *H*-2), 5.09 (s, 12H, b), 4.79 (s, 18H, *H*-1), 4.59 (dd, *J* = 10.5, 5.7 Hz, 36H, i), 4.54 (s, 36H, h), 4.49 (t, *J* = 5.0 Hz, 12H, c), 4.19 (dd, *J* = 12.3, 5.0 Hz, 18H, *H*-6), 4.14–4.07 (m, 18H, j′), 4.02 (dd, *J* = 12.3, 1.8 Hz, 18H, *H*-6′), 3.89 (m, 18H, j), 3.86 (t, *J* = 5.2 Hz, 12H, d), 3.62–3.59 (m, 18H, *H*-5), 3.55–3.48 (m, 24H, e, f), 3.46 (s, 36H, g′), 3.40 (s, 36H, g), 2.11 (s, 54H, COC*H*_3_), 2.06 (s, 54H, COC*H*_3_), 2.01 (s, 54H, COC*H*_3_), 1.96 (s, 54H, COC*H*_3_). ^13^C-NMR (150 MHz, CDCl_3_) δ 170.6 (C=O), 169.9 (C=O), 169.6 (C=O), 169.1 (C=O), 155.4 (CO-aromatic), 145.5 (C_B_-triazole), 144.6 (C_A_-triazole), 143.5 (C**_p_**-aromatic), 124.2 (C_A_H-triazole), 123.7 (C_B_H-triazole), 121.8 (C**_m_**-aromatic), 115.3 (C**_O_**-aromatic), 97.5 (C-1), 71.0 (C_f_), 70.3 (C_e_), 69.7 (C_g_), 69.4 (C_d_), 69.2 (C-2), 69.1 (C-3), 68.9 (C-4), 68.8 (C-5), 66.3 (C_j_), 65.7 (C_b_), 64.8 (C_h_), 62.2 (C-6), 50.3 (C_i_), 49.5 (C_c_), 45.3 (C_q_), 20.8 (C*OC*H_3_), 20.7 (C*OC*H_3_), 20.7 (CO*C*H_3_), 20.7 (CO*C*H_3_). ^31^P-NMR (243 MHz, CDCl_3_) δ 9.54. MALDI-TOF: [M + H]^+^ calcd for C_450_H_607_N_75_O_222_P_3_: 10705.772; found, 10706.833.

*Synthesis of***40**. Compound **39** (200 mg, 0.02 mmol) and sodium methoxide (16 μL from 1 M solution in MeOH) in 3 mL of methanol were stirred for 4 h and the mixture was treated following the general procedure (*III*) described above. Deprotected compound **40** (150 mg, quant.) was obtained as a white solid. ^1^H-NMR (600 MHz, D_2_O) δ 8.05 (s, 1H), 7.92 (s, 3H), 6.74 (dd, *J* = 42.4, 7.8 Hz, 4H), 5.09 (s, 2H), 4.75 (s, 3H), 4.61–4.51 (m, 6H), 4.45 (s, 6H), 4.02 (s, 3H), 3.83 (s, 6H), 3.71 (d, *J* = 10.8 Hz, 3H), 3.63 (ddd, *J* = 25.4, 15.8, 7.5 Hz, 8H), 3.36 (dd, *J* = 75.3, 44.3 Hz, 12H), 3.11–3.02 (m, 3H). ^13^C-NMR (150 MHz, D_2_O) δ 215.7, 154.9, 144.3, 143.8, 142.9, 125.3, 125.1, 121.7, 115.8, 99.5, 72.8, 70.5, 69.9, 69.7, 68.9, 68.7, 68.3, 66.3, 65.4, 63.6, 61.4, 60.6, 50.1, 49.9, 44.8, 30.2, 29.7, 29.6, 29.4, 29.3, 29.2. ^31^P-NMR (243 MHz, D_2_O) δ 10.06. MALDI-TOF: [M + H]^+^ calcd for C_306_H_463_N_75_O_150_P_3_: 7681.011; found, 7681.383.

## 4. Conclusions

The synthesis of a new library of glycodendrimers possessing pegylated sugars in the aglycones with different valences from 2 to 18, has been achieved in satisfying yields provided by the highly efficient click chemistry. The above set of assay is relevant in demonstrating the relative binding abilities of these glycodendrimers for their respective lectins. In DLS measurements, the use of kinetics of mannodendrimers-protein aggregation in presence of the mannose-specific lectin ConA gave useful preliminary results. We noticed that, highly substituted glycodendrimers demonstrated a higher capacity to crosslink ConA by forming insoluble complexes within a short time frame. Hence, the results obtained herein suggest that dendrimers harboring pegylated mannosides provided gradual and functional binding interactions with a model mannose-binding protein such as ConA. Perhaps, more importantly, is the fact that the synthetic strategy that we recently coined “onion peel strategy” offers advantages over classical ones [32,47,54,55,56,57]. Indeed, by choosing the cyclotriphosphazene core having six functional groups available (an A_6_ core) together with an AB_3_ pentaerythritol scaffold at the next generation, provided a dendrimeric architecture from which, a total of 18 surface groups can be achieved by a convergent synthesis in a short synthetic sequence leading to a G1 generation. Ongoing activities, aimed at measuring the inhibition of biofilm formation from uropathogenic *E. coli*, are underway.

## Supplementary Materials

Supplementary materials are available online.
