# Supplementary material for: Effect of Dendrimer Generation and Aglyconic Linkers on the Binding Properties of Mannosylated Dendrimers Prepared by a Combined Convergent and Onion Peel Approach
Source: Molecules. 2018 Jul 28;23(8):1890. doi: 10.3390/molecules23081890 (PMC6222628; doi:10.3390/molecules23081890)
Supplement: Supplementary file 1 [file molecules-23-01890-s001.pdf]

Effect of Dendrimer Generation and Aglyconic Linkers on the Binding Properties of Mannosylated Dendrimers Prepared by a Combined Convergent and Onion Peel Approach

Celia Sehad[1], Tze Chieh Shiao[1], Lamyaa M. Sallam,[1] Abdelkrim Azzouz[1] and René Roy[1,2,*]

[1] Department of Chemistry, University of Québec a Montréal, P.O. Box 8888, Succ. Centre-Ville, Montréal, Québec H3C 3P8, Canada, E-mail : roy.rene@uqam.ca; Tel.: +1 514-987-3000, ext. 2546

[2] Glycovax Pharma Inc., 424 Guy, Suite 202, Montreal, Quebec, Canada, H3J 1S6

* Correspondence: rroy@glycovax.com;

Supplementary material

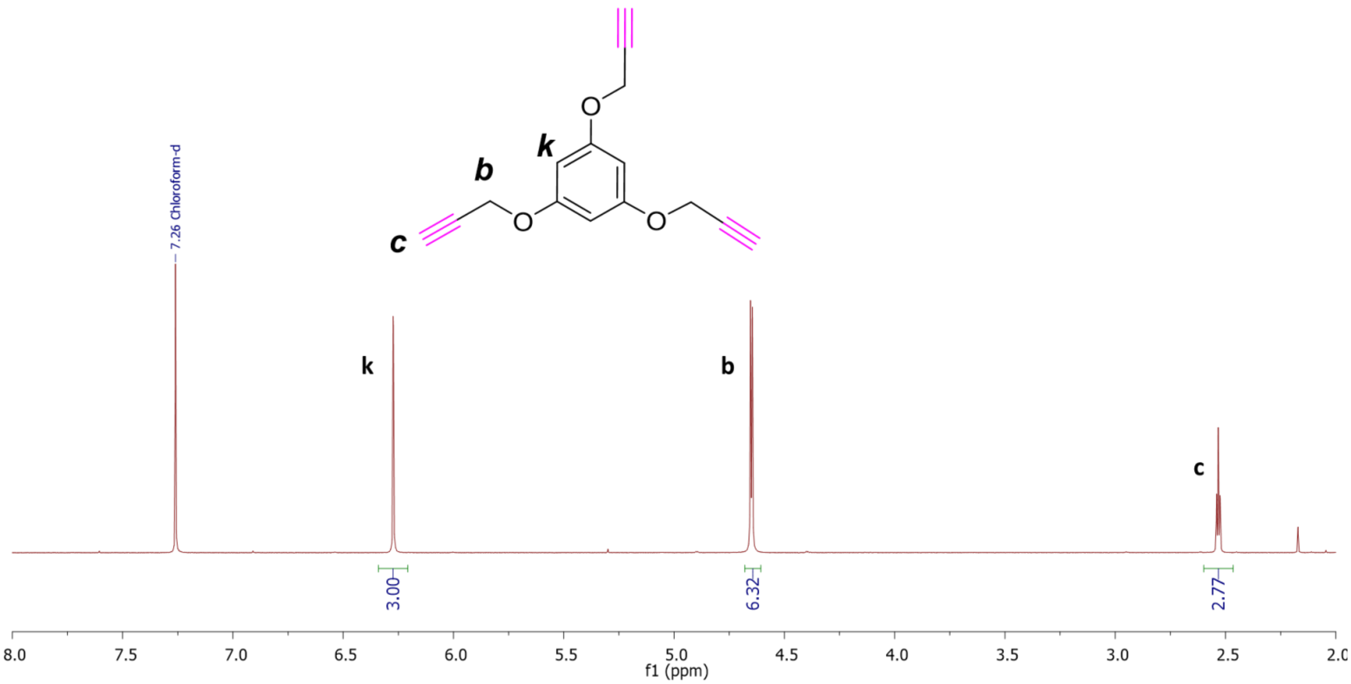

^{1}H- NMR spectrum of compound 2.

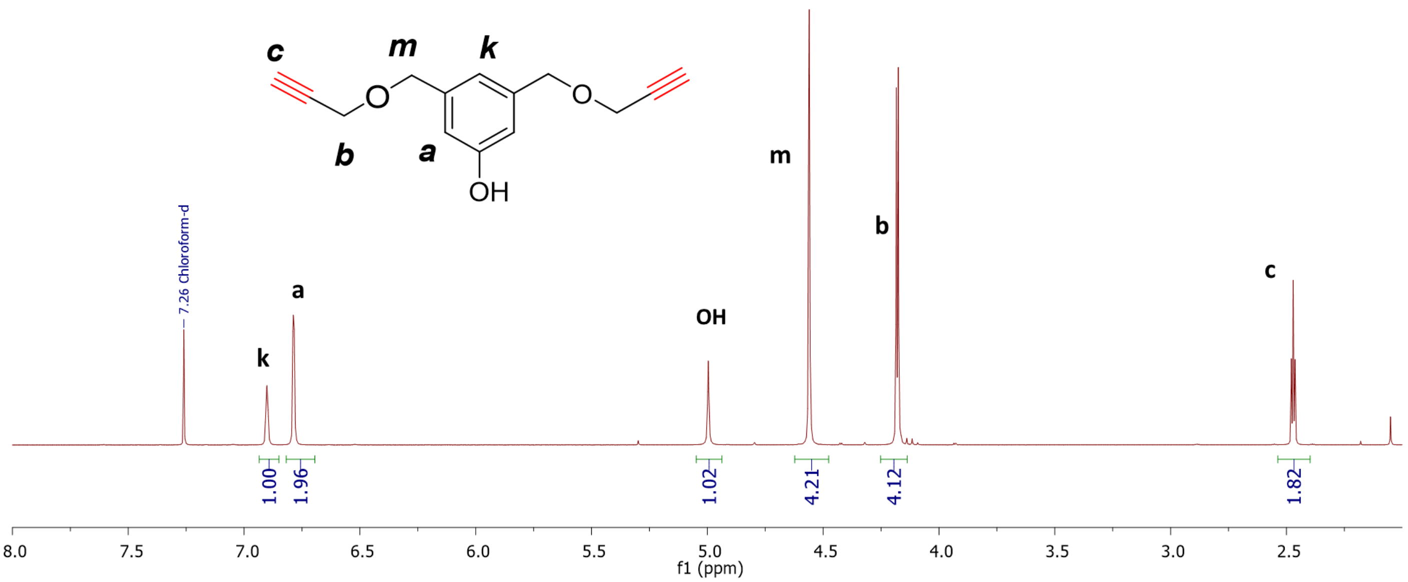

^{1}H- NMR spectrum of compound 7.

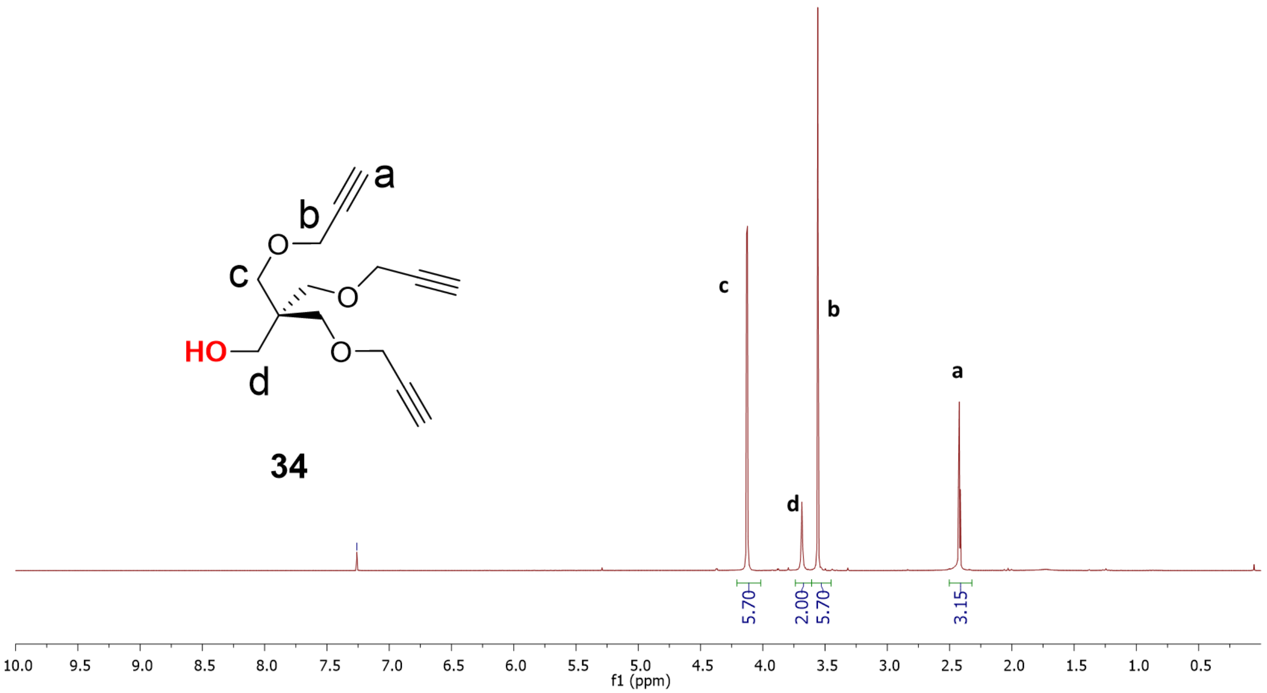

^{1}H- NMR spectrum of compound 9.

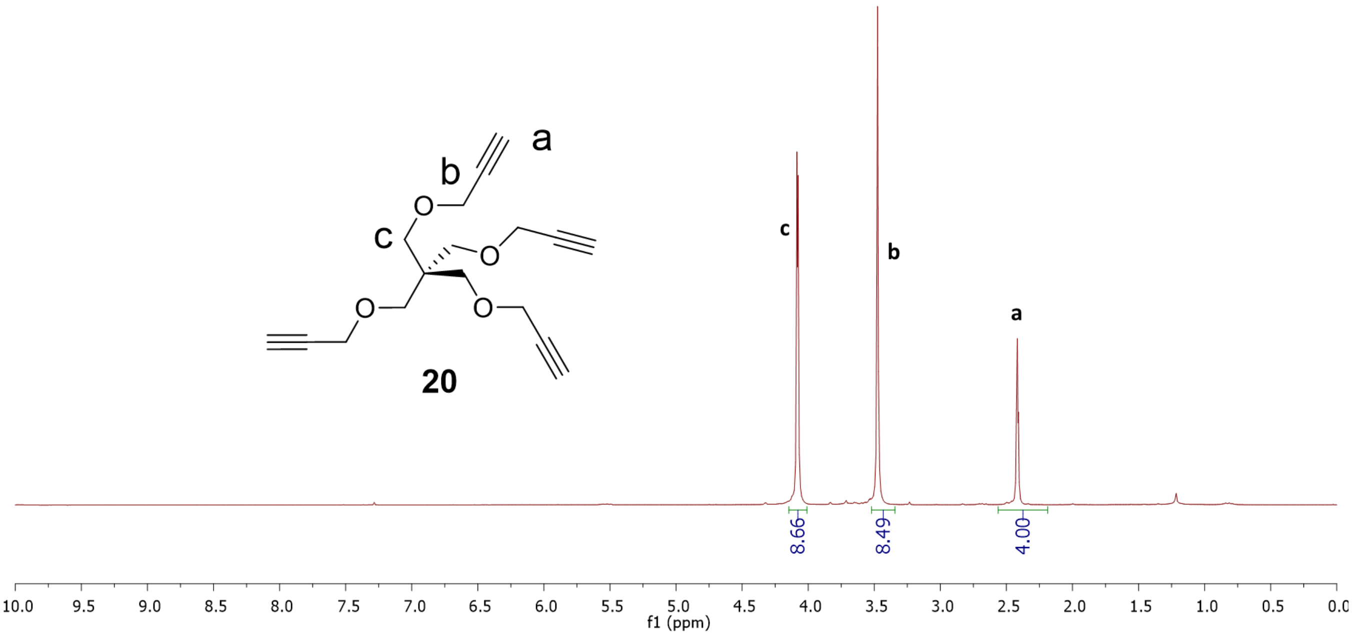

^{1}H- NMR spectrum of compound 10.

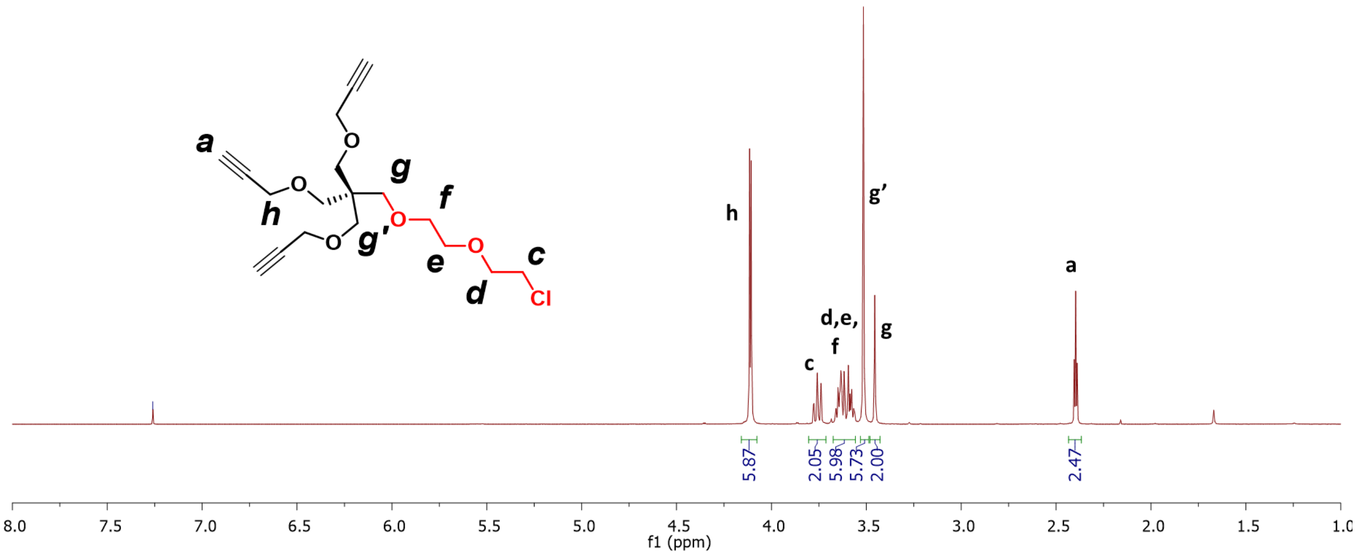

^{1}H- NMR spectrum of compound 11.

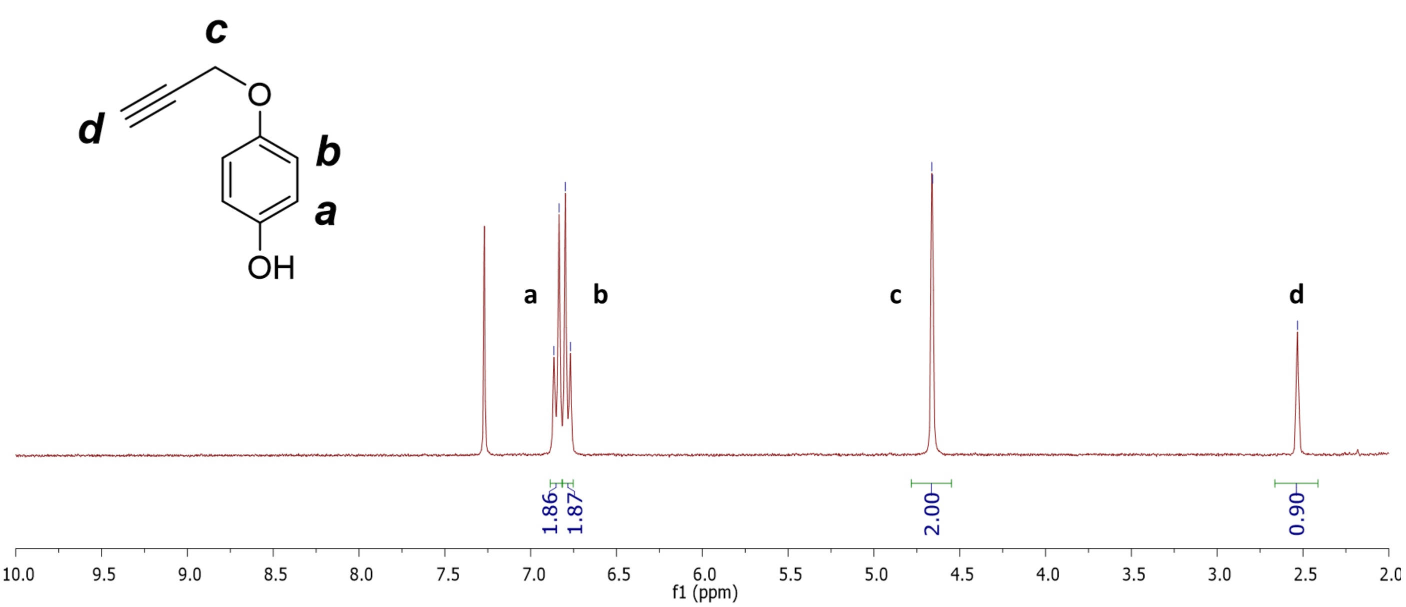

^{1}H- NMR spectrum of compound 13.

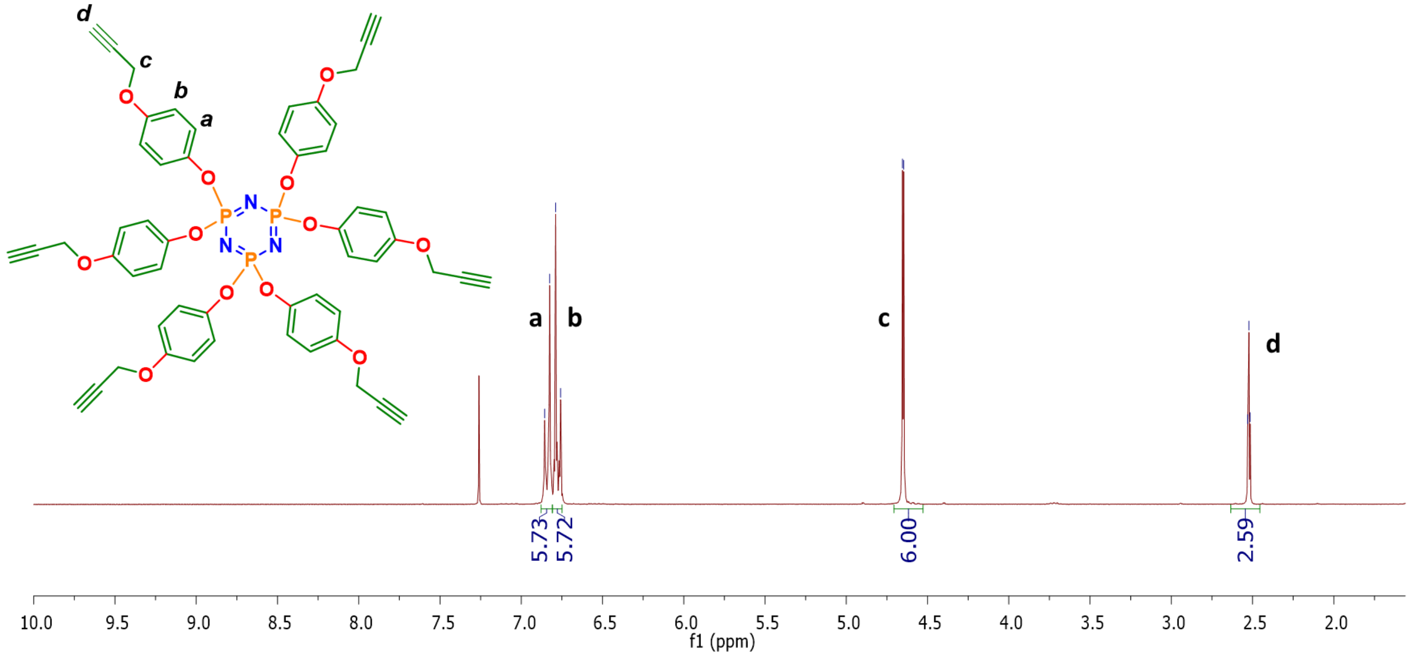

^{1}H- NMR spectrum of compound 15.

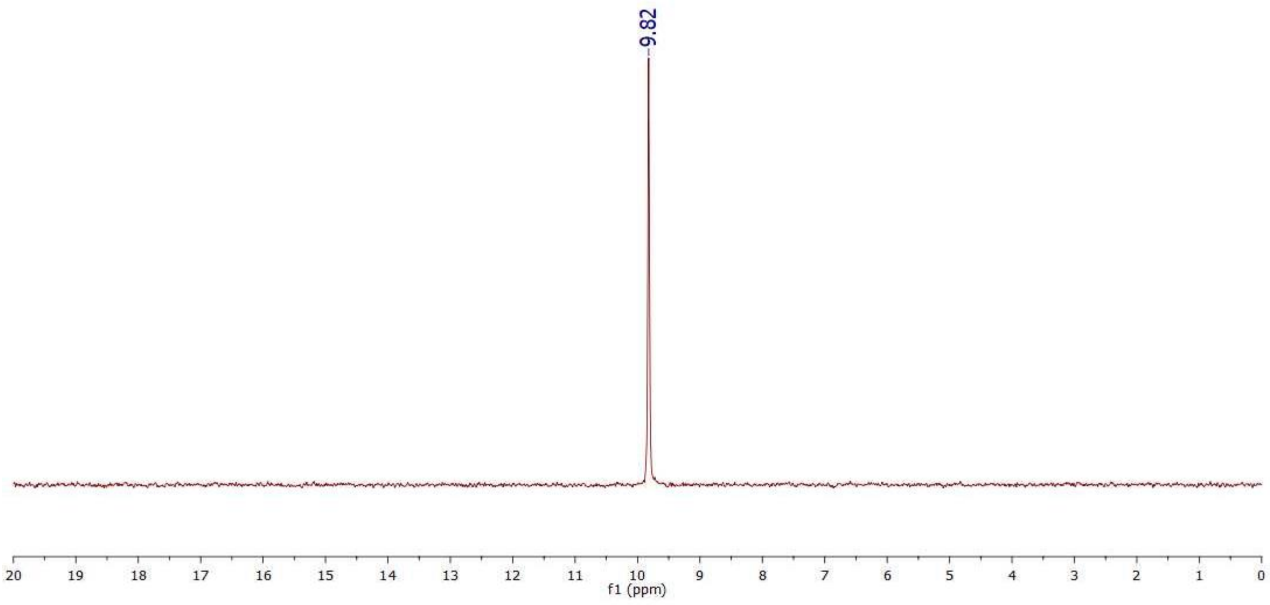

^{31}P- NMR spectrum of compound 15.

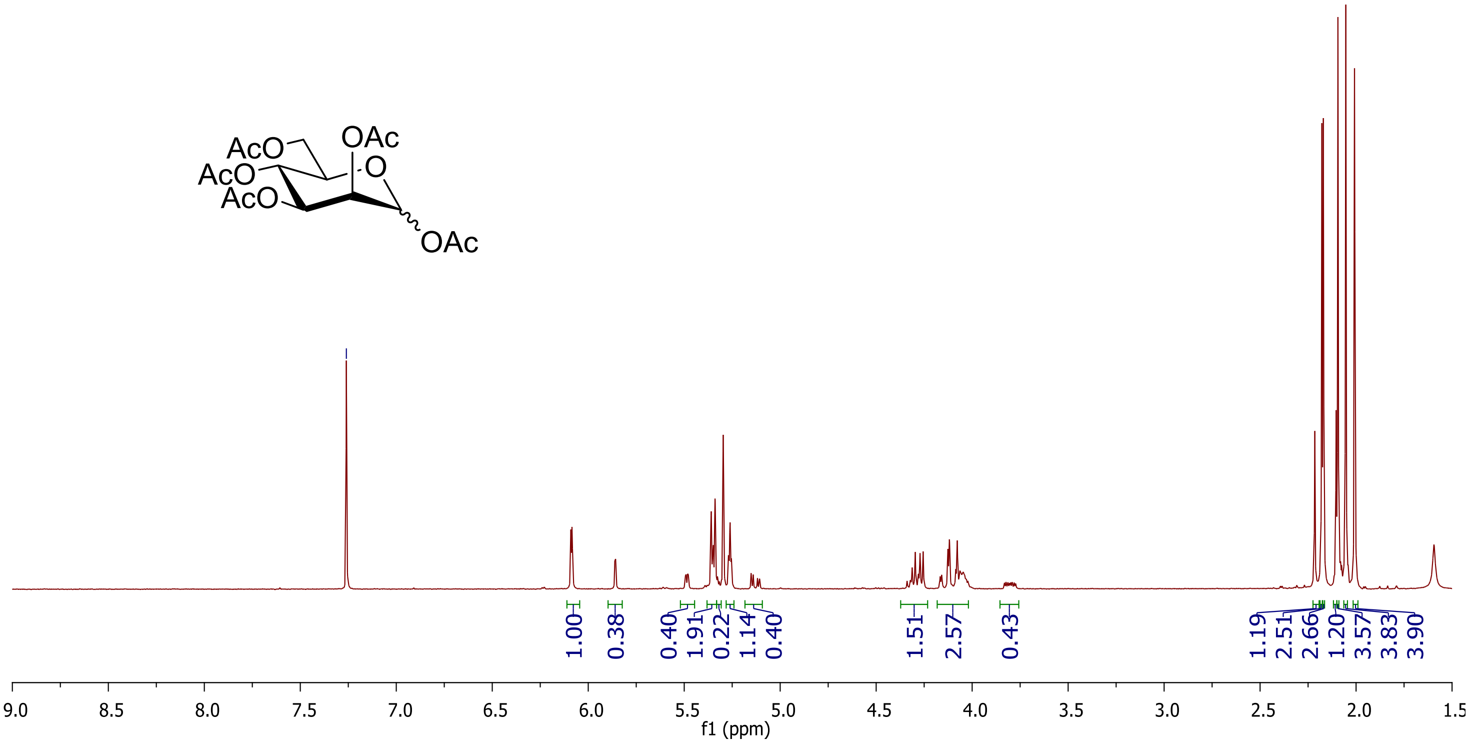

^{1}H-NMR spectrum of compound 17.

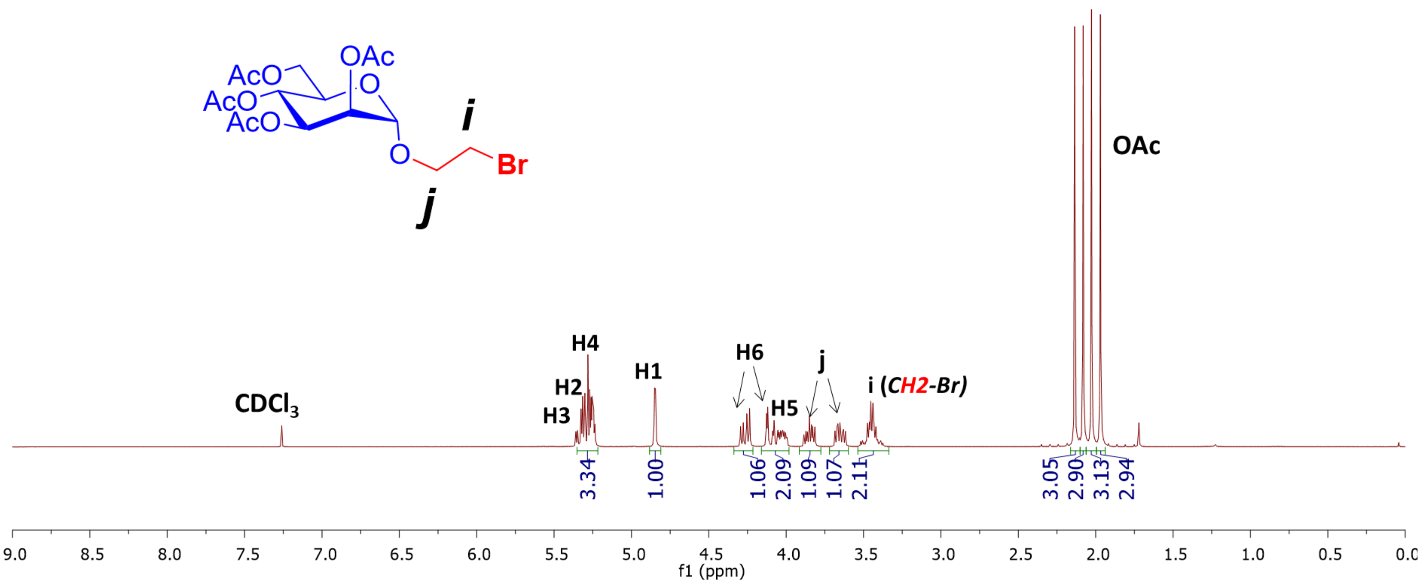

^{1}H-NMR spectrum of compound 18.

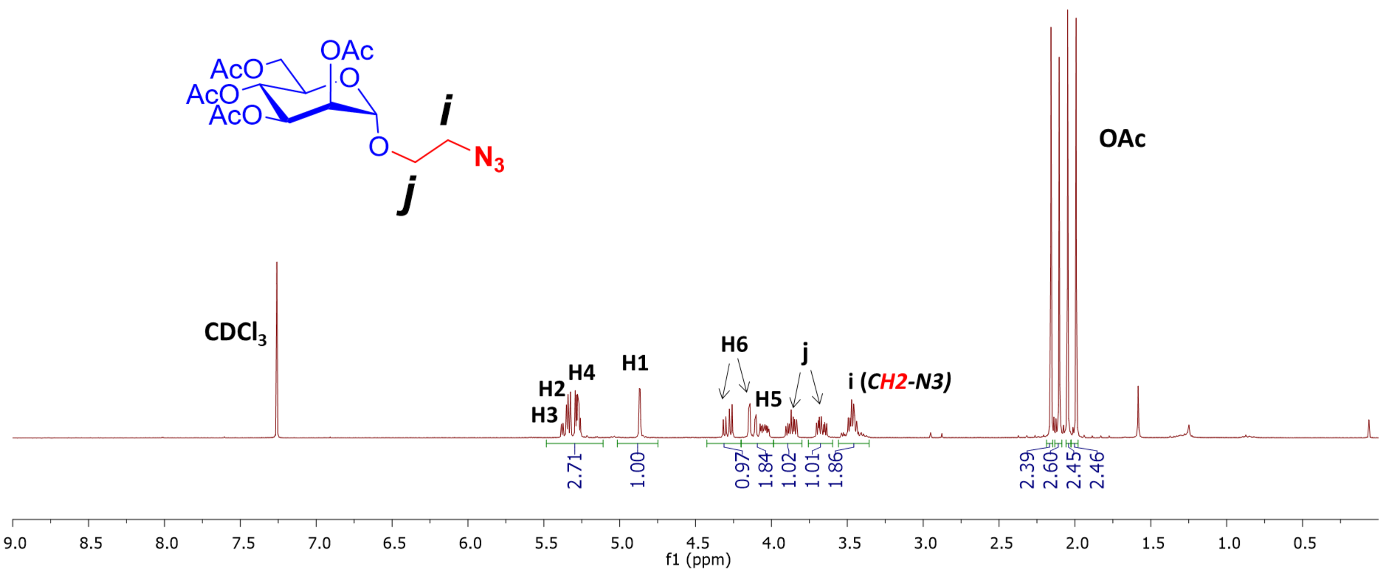

^{1}H-NMR spectrum of compound 19.

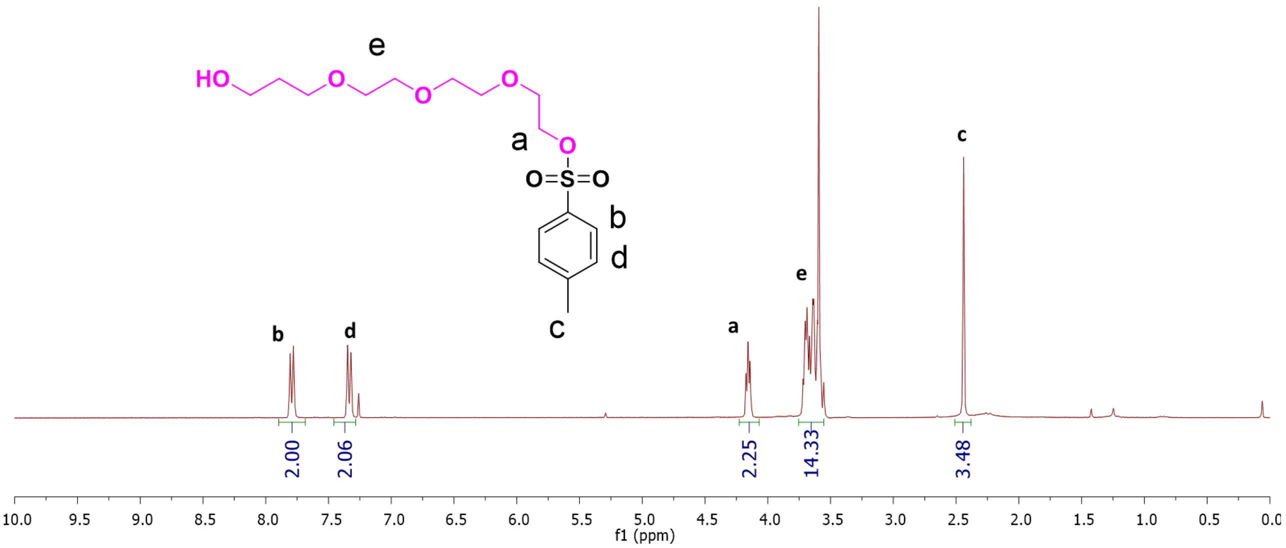

^{1}H-NMR spectrum of compound 20.

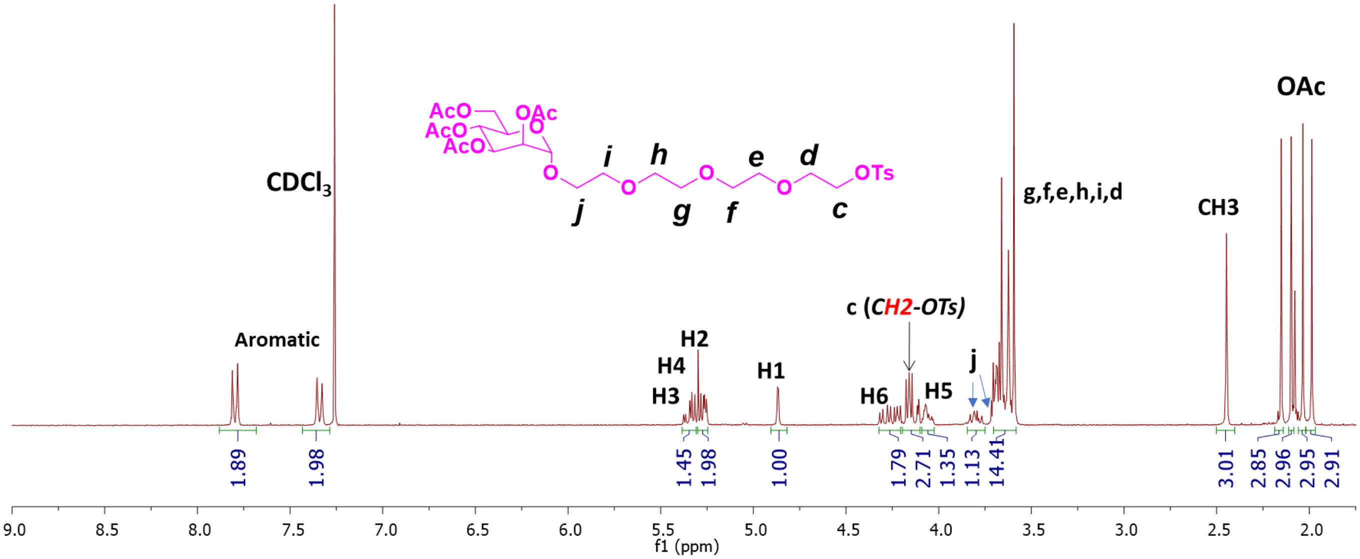

^{1}H-NMR spectrum of compound 21.

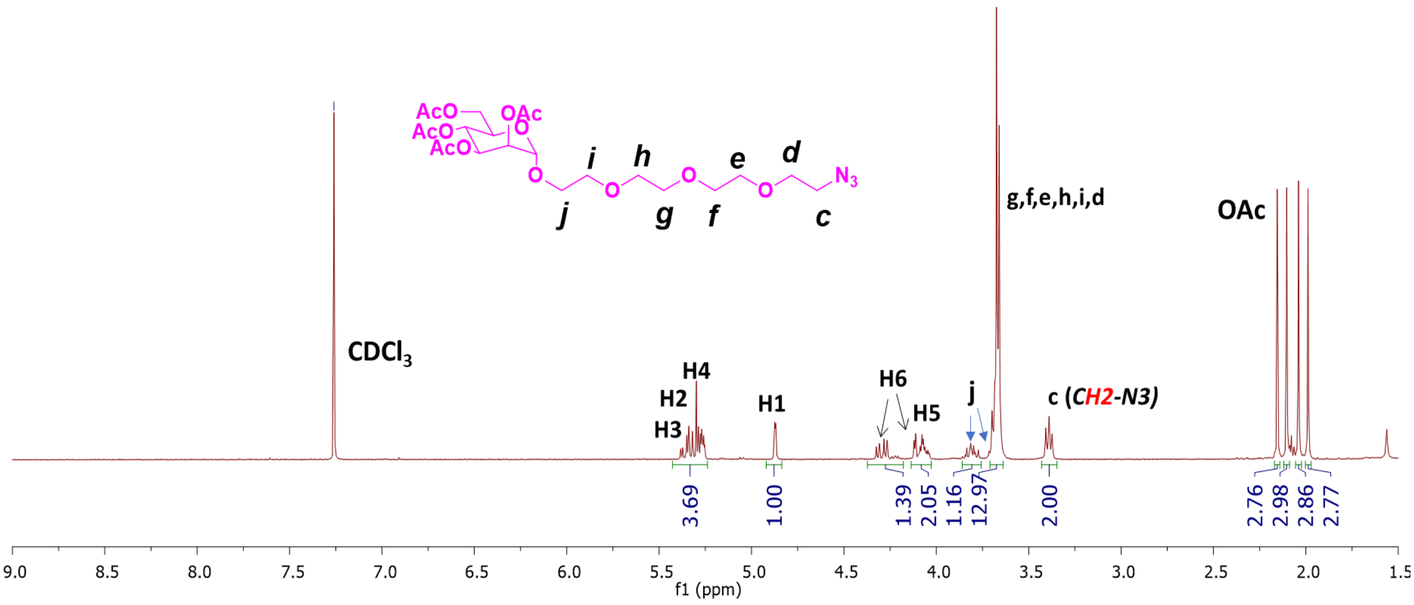

^{1}H-NMR spectrum of compound 22.

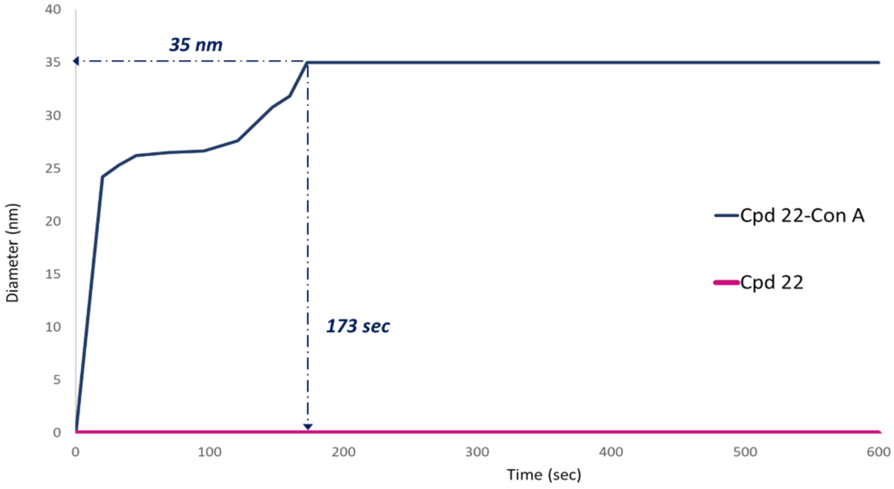

Fig 1. Synthesis DLS of Cpd **22** (deprotected monomer) Crosslinked with Con A.

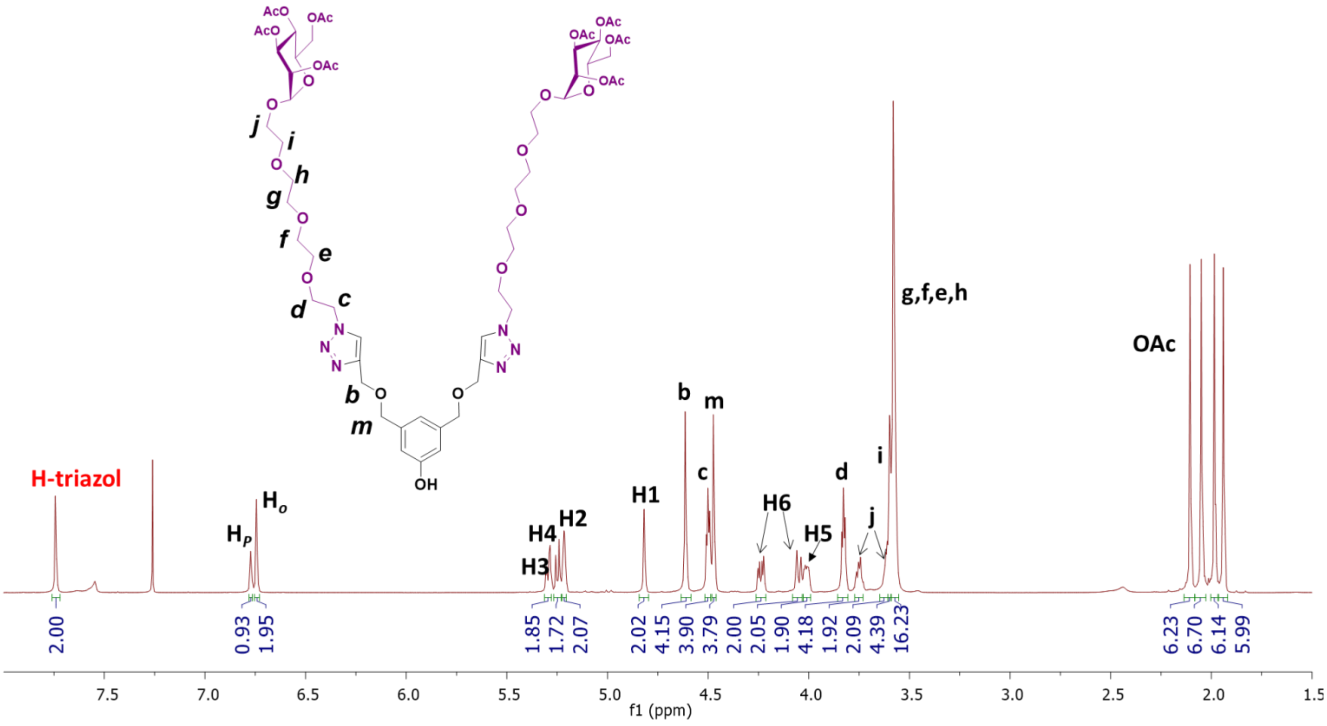

^{1}H- NMR spectrum of compound 23.

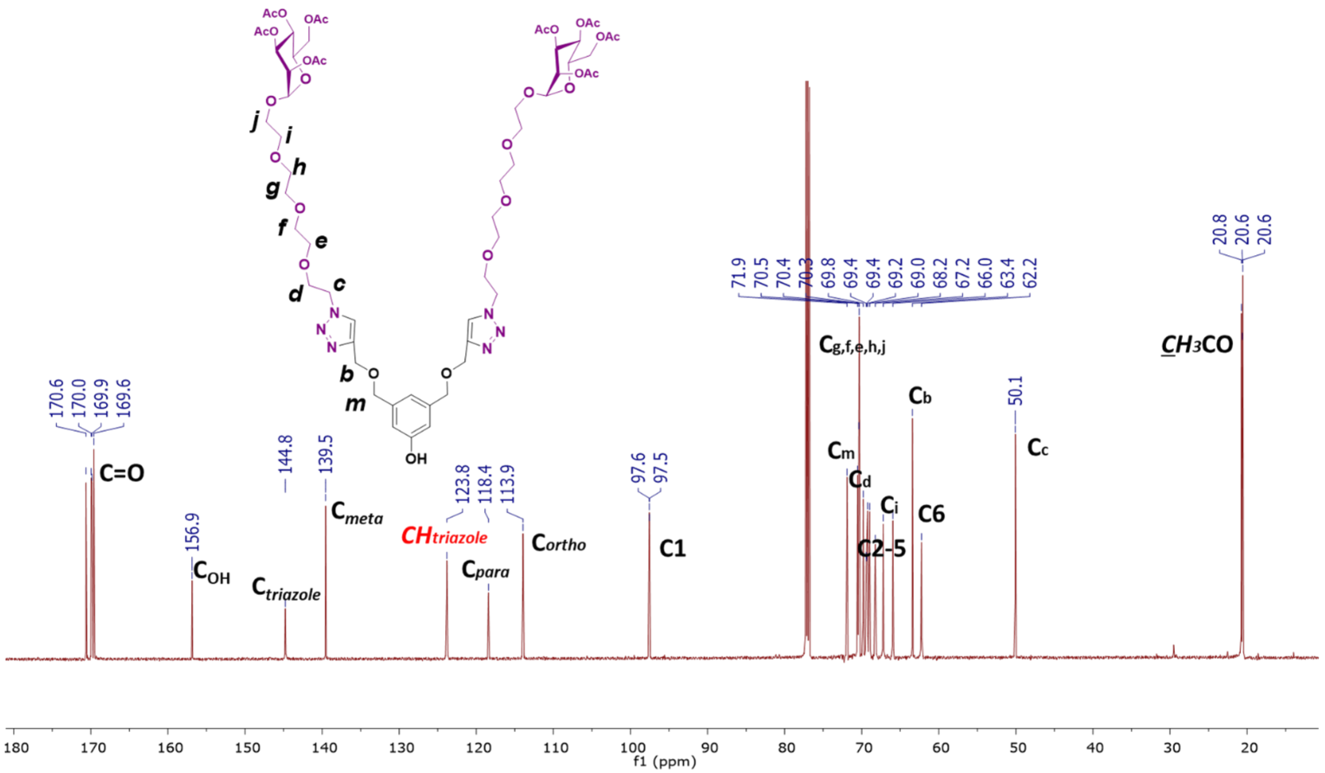

^{13}C- NMR spectrum of compound 23.

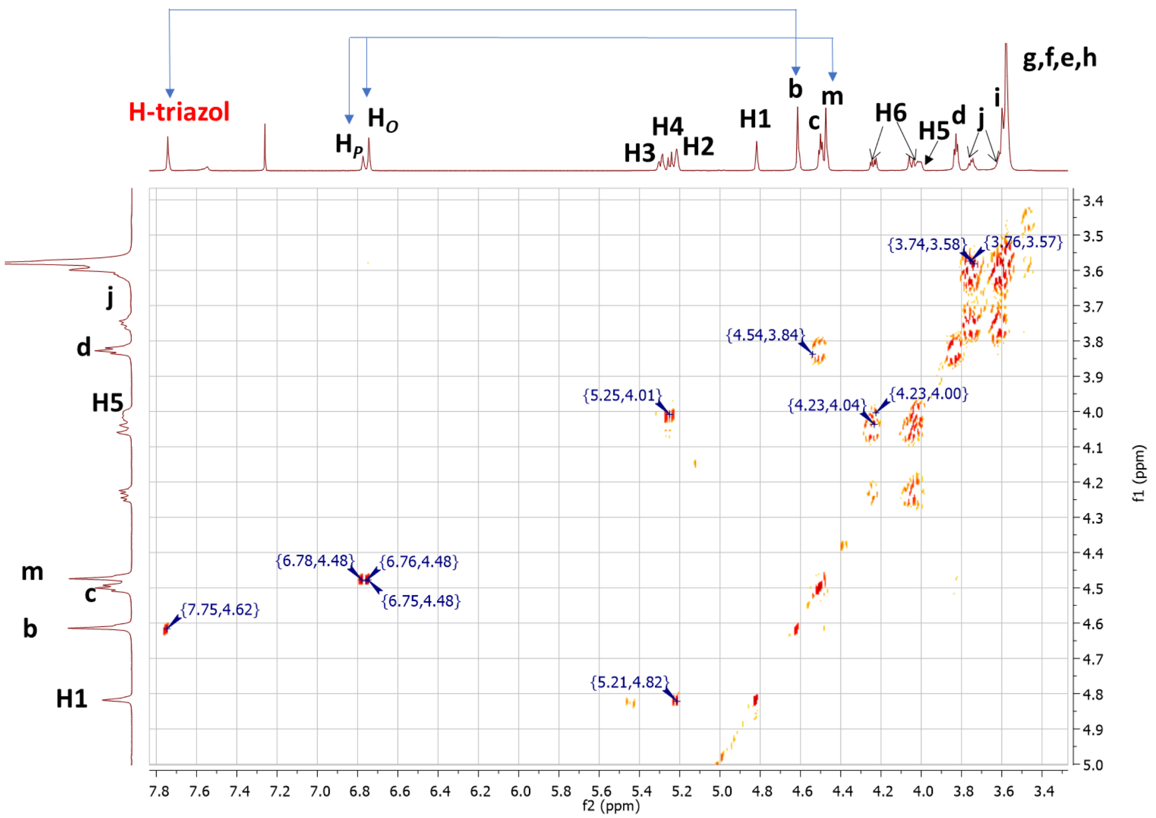

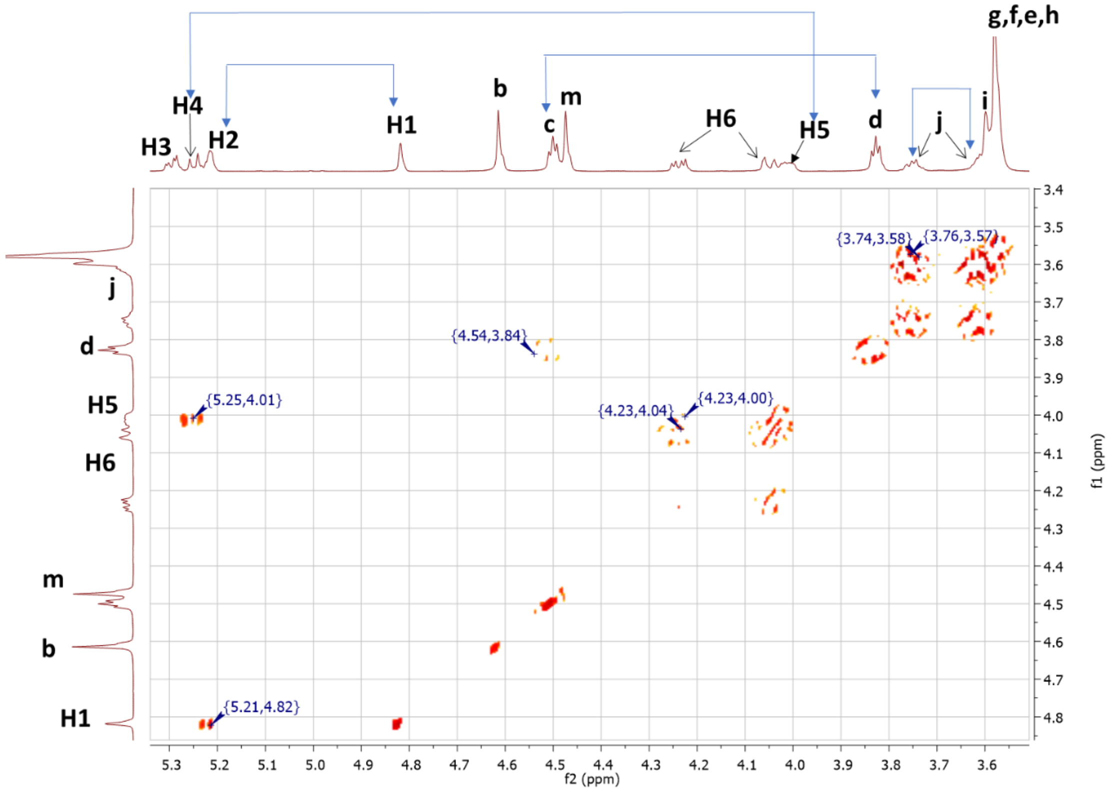

2D NMR – COSY: ^1H-^1H correlation spectrum of compound 23.

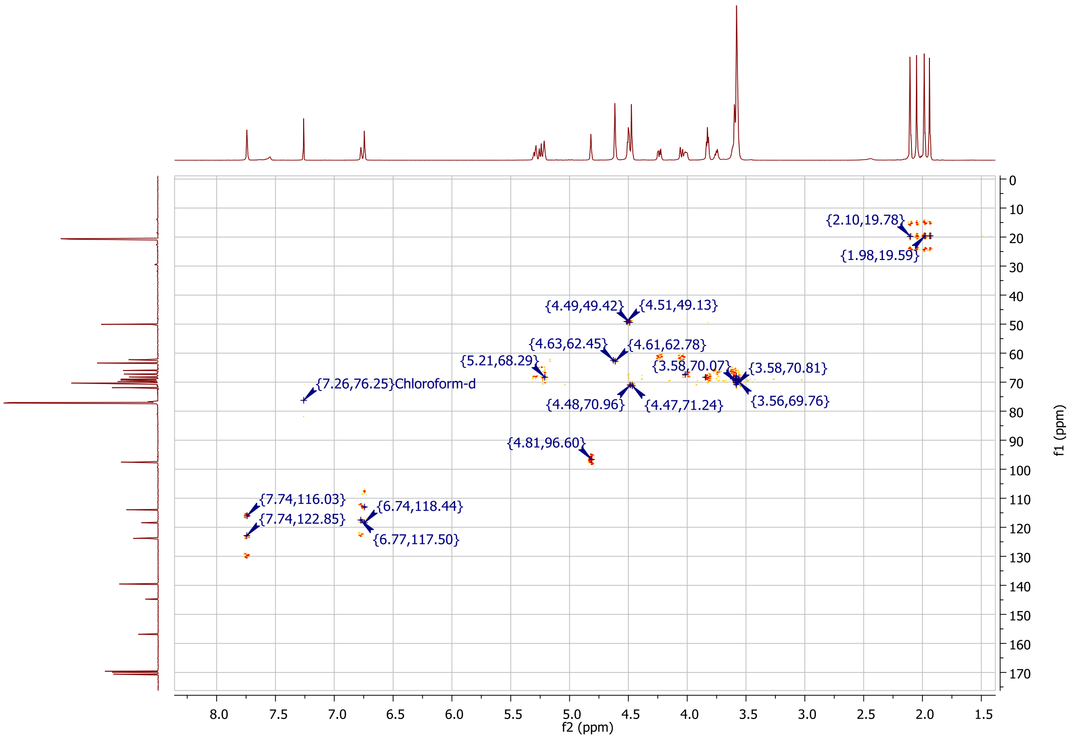

2D NMR – HSQC: ^{1}H-^{13}C correlation spectrum of compound 23.

Compound Table

Compound Label	RT	Mass	Abund	Formula	Tgt Mass	Diff (ppm)
Cpd 1: C56 H86 N6 O29	0.1	1306.5502	304616	C56 H86 N6 O29	1306.5439	4.83

Compound Label	RT	Algorithm	Mass
Cpd 1: C56 H86 N6 O29	0.1	Find By Formula	1306.5502

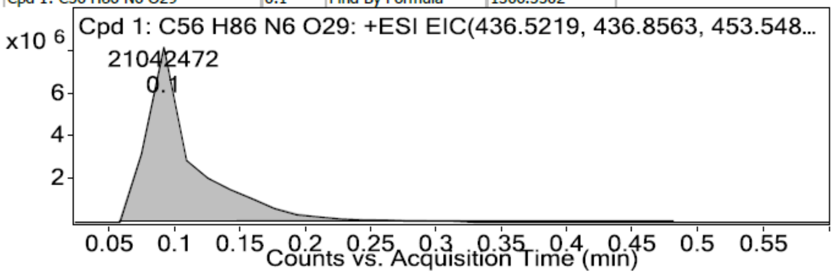

MS Spectrum

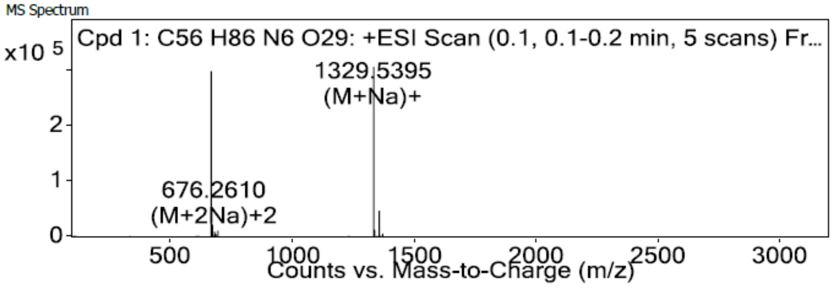

HRMS of compound 23.

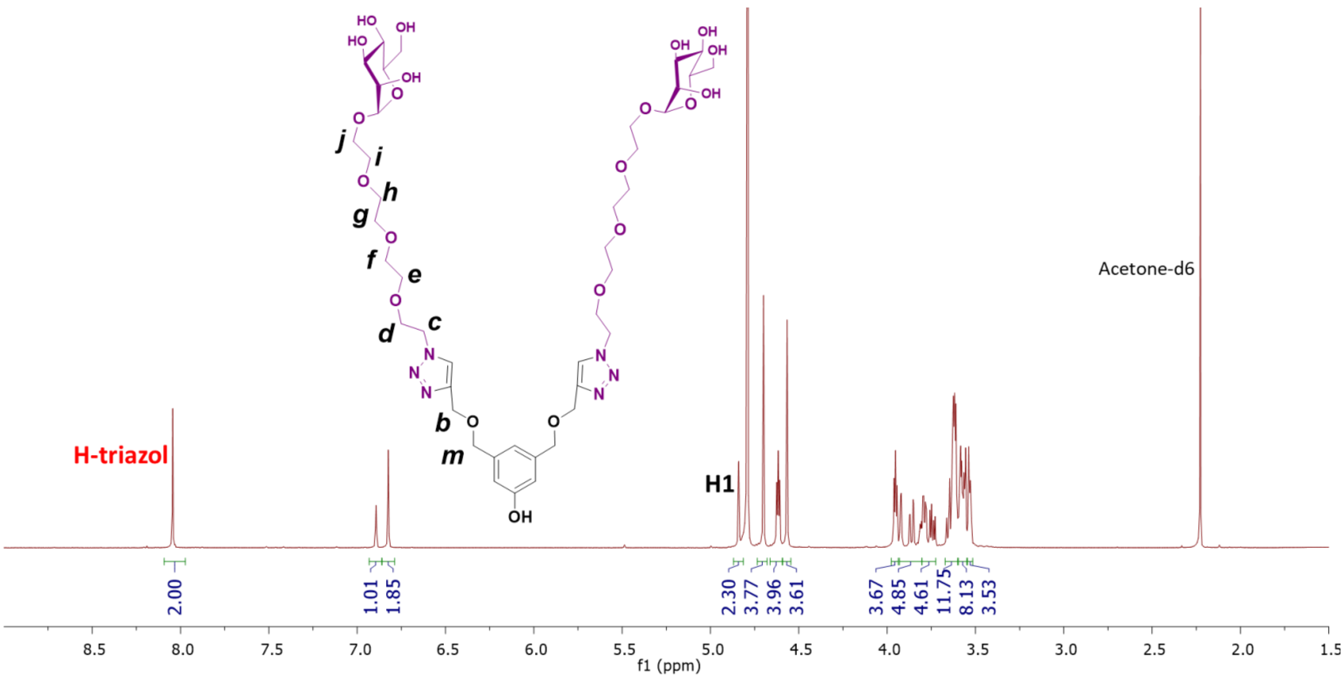

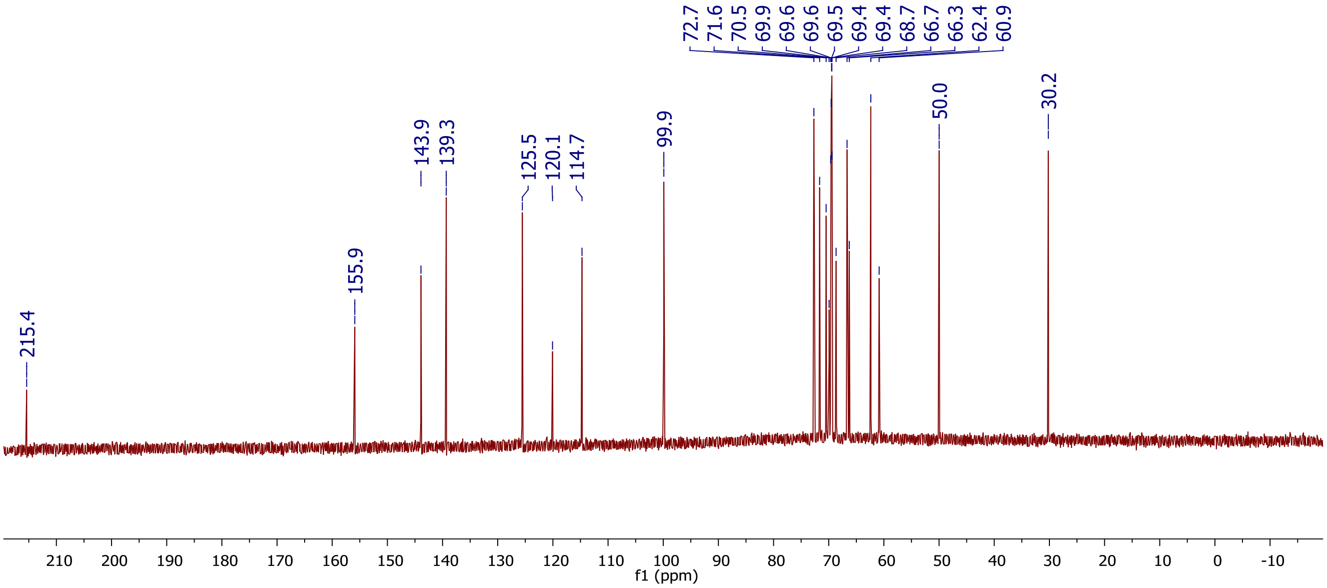

^{1}H- and ^{13}C- NMR spectrum of compound 24.

Compound Table

Compound Label	RT	Mass	Abund	Formula	Tgt Mass	Diff (ppm)
Cpd 1: C40 H70 N6 O21	0.1	970.465	5215	C40 H70 N6 O21	970.4594	5.74

Compound Label	RT	Algorithm	Mass
Cpd 1: C40 H70 N6 O21	0.1	Find By Formula	970.465

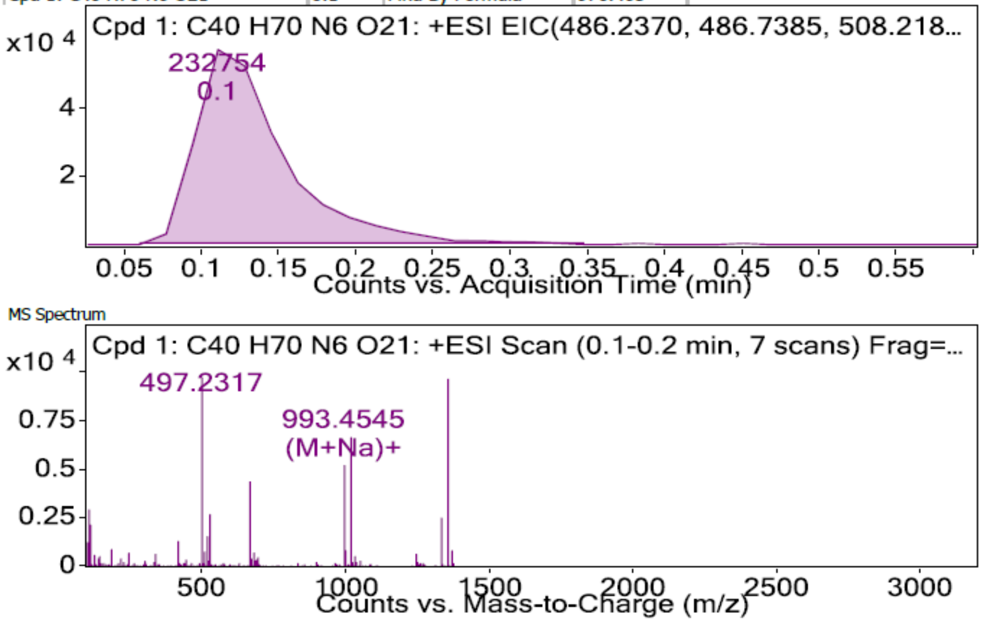

HRMS of compound 24.

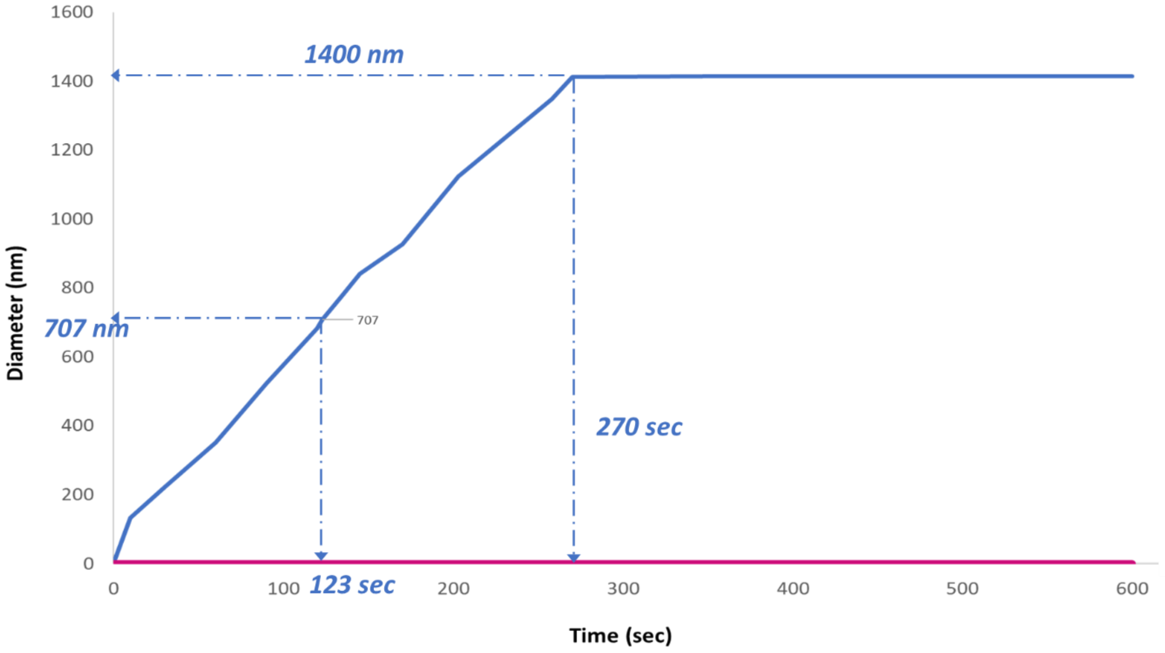

Fig 2. Synthesis DLS of Cpd **24** (Bis) Crosslinked with Con A.

			Size (d.nm):	% Number:	St Dev (d.n...
Z-Average (d.nm):	2979	Peak 1:	3253	100.0	940.0
PdI:	0.279	Peak 2:	0.000	0.0	0.000
Intercept:	0.861	Peak 3:	0.000	0.0	0.000
Result quality :	Refer to quality report				

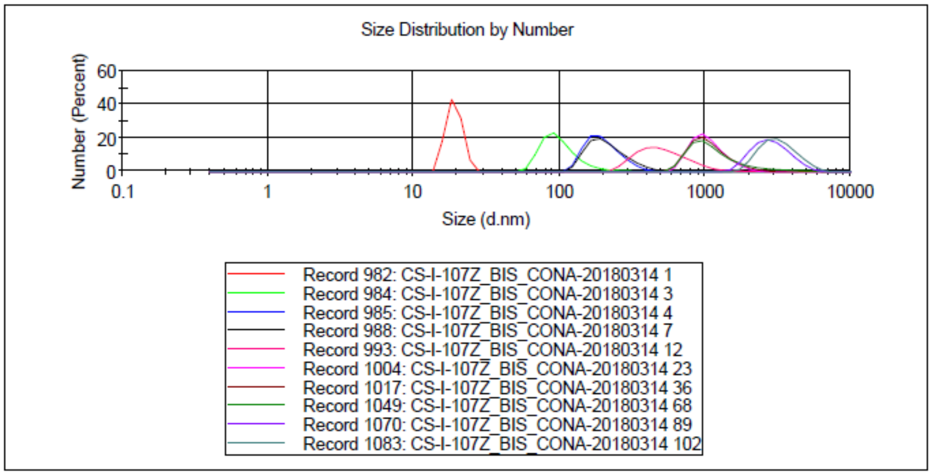

DLS-Size distribution of compound 24.

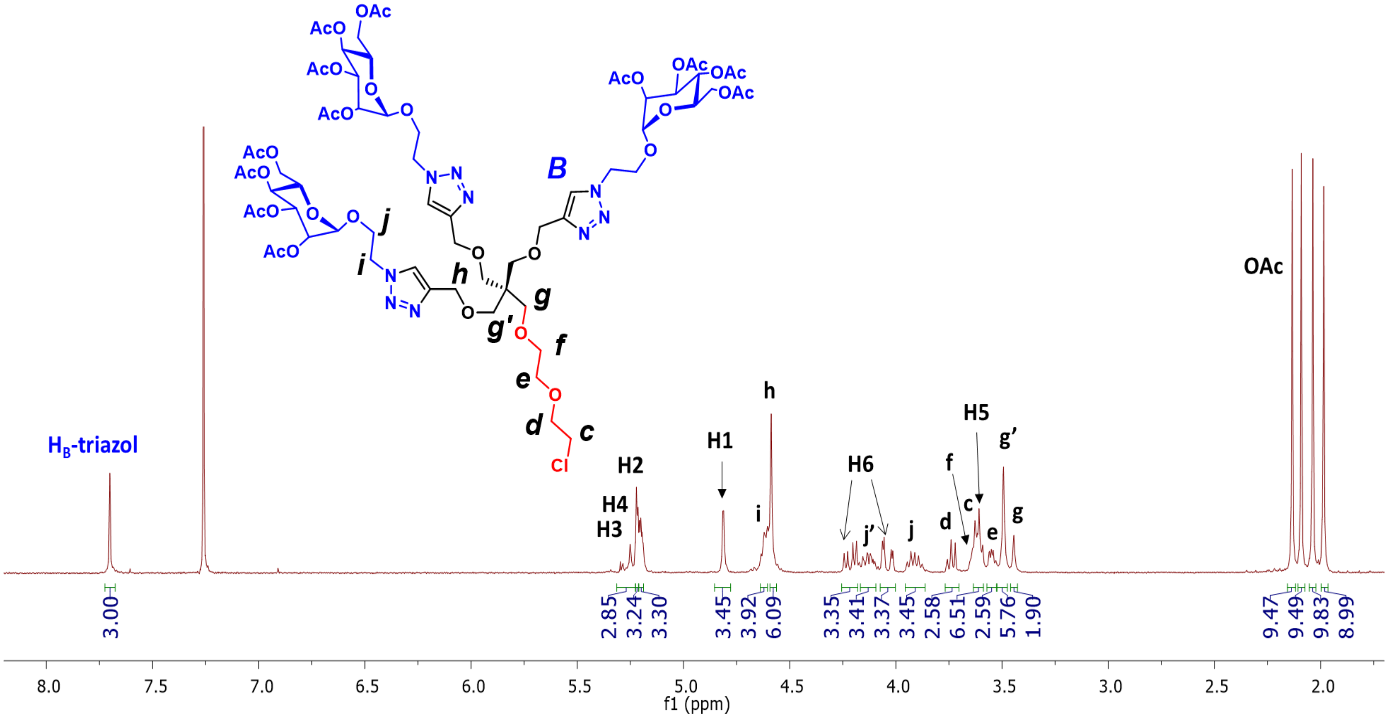

^{1}H- NMR spectrum of compound 25.

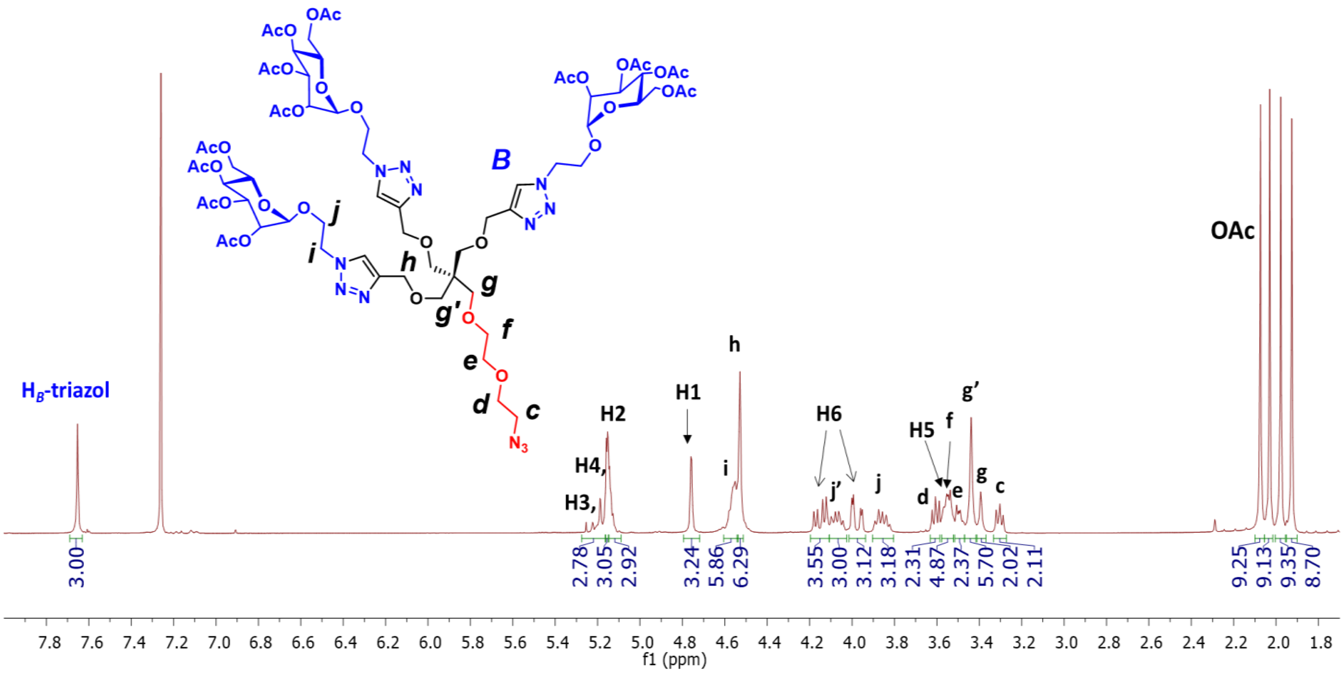

^{1}H- NMR spectrum of compound 26.

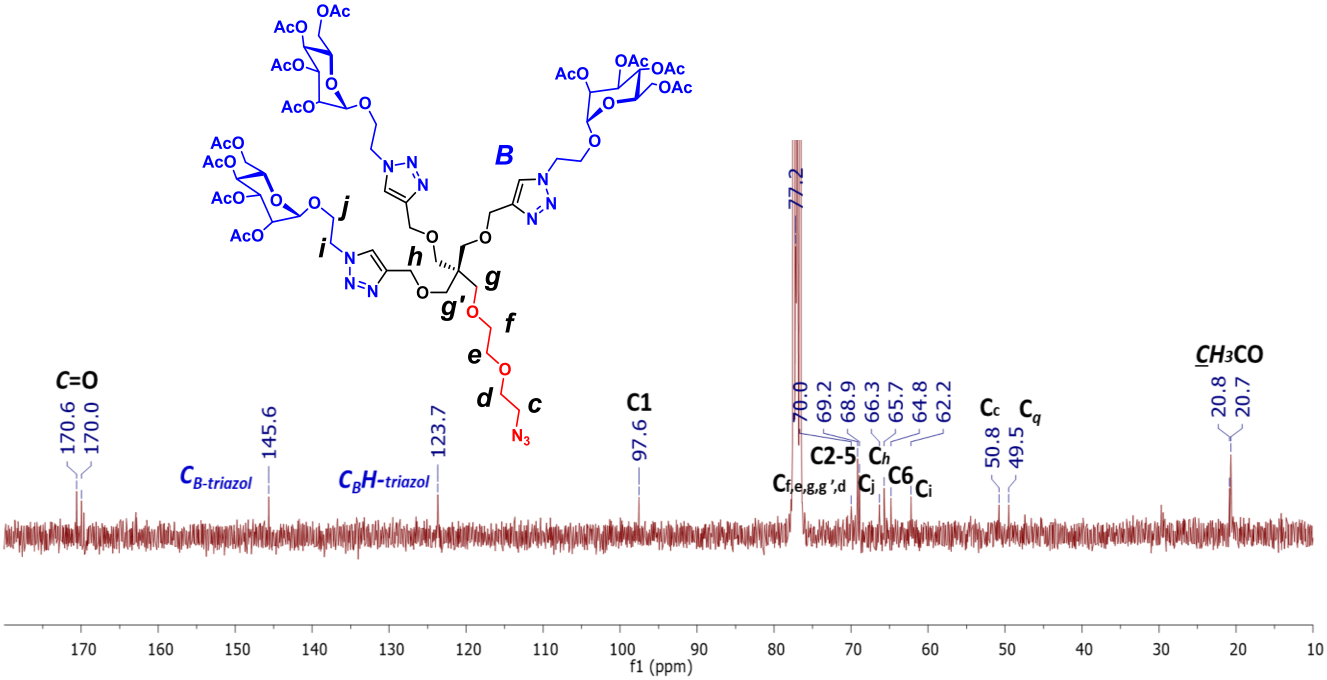

^{13}C- NMR spectrum of compound 26.

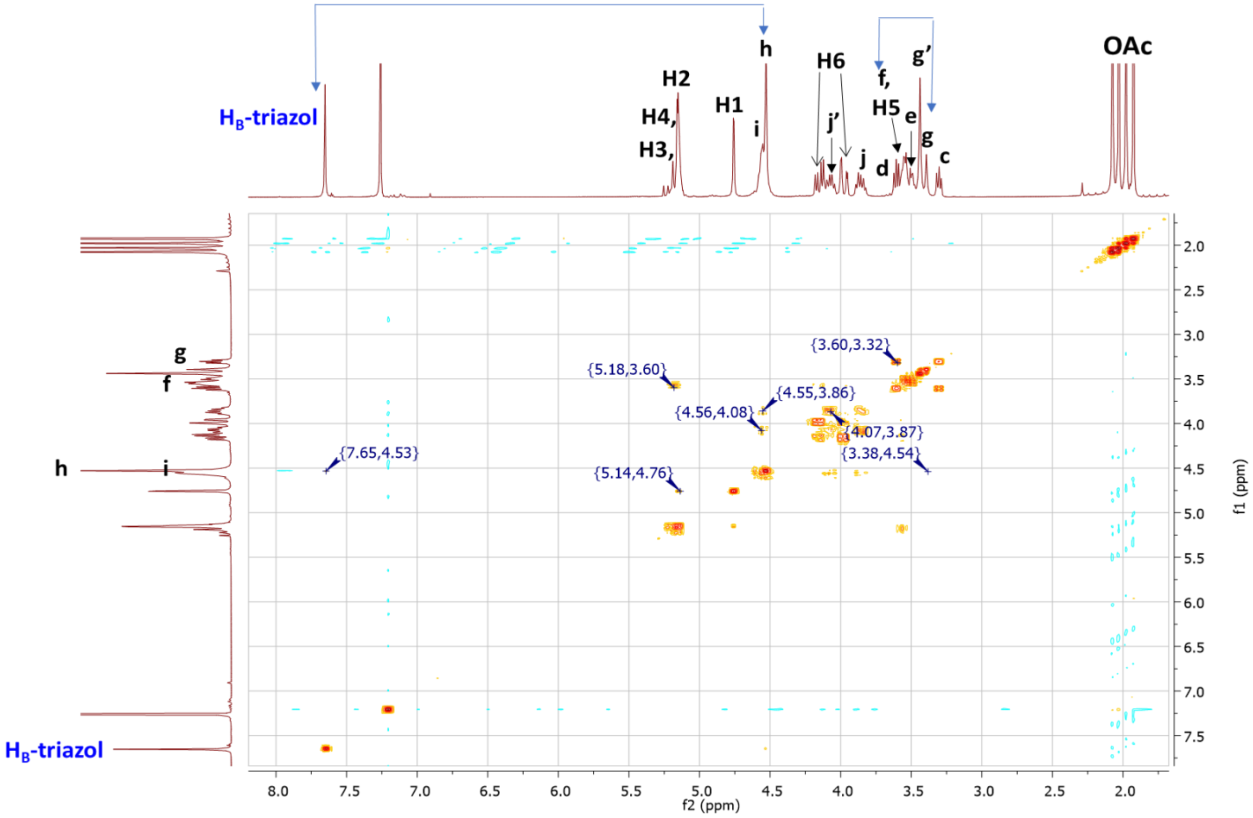

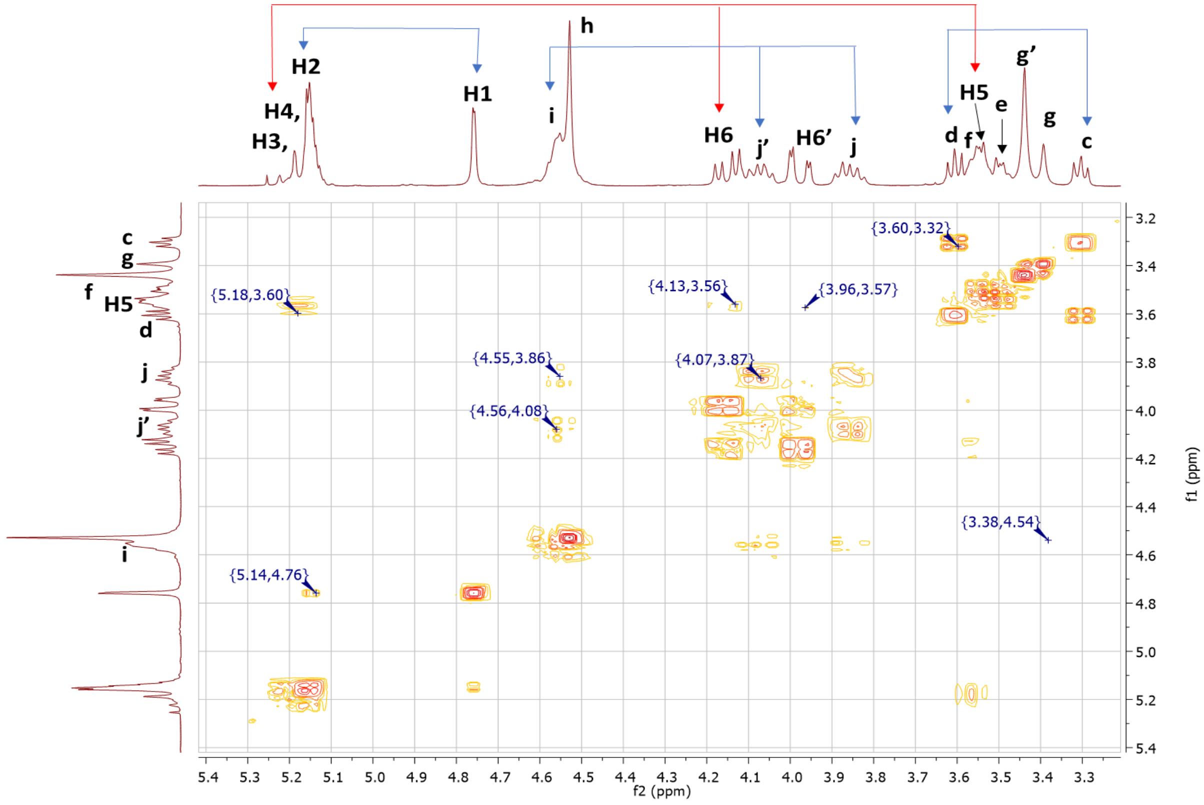

2D NMR – COSY: ^{1}H-^{1}H correlation spectrum of compound 26.

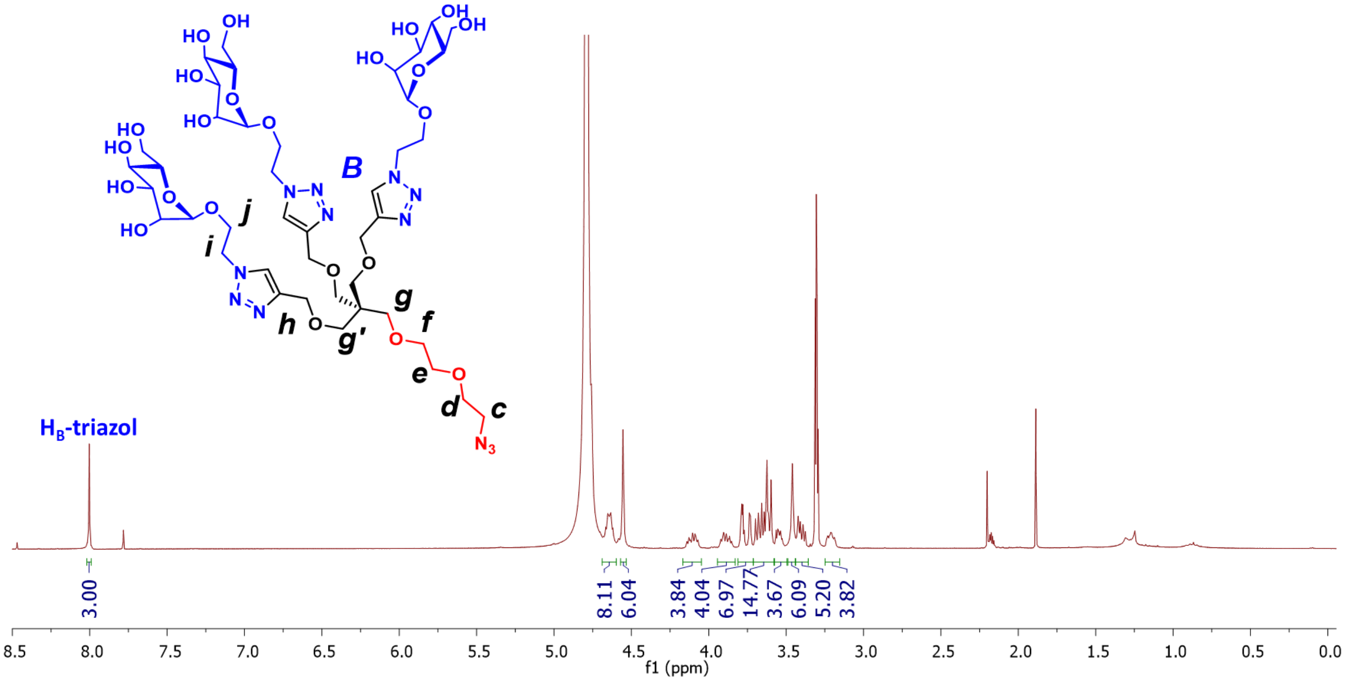

^{1}H- NMR spectrum of compound 27.

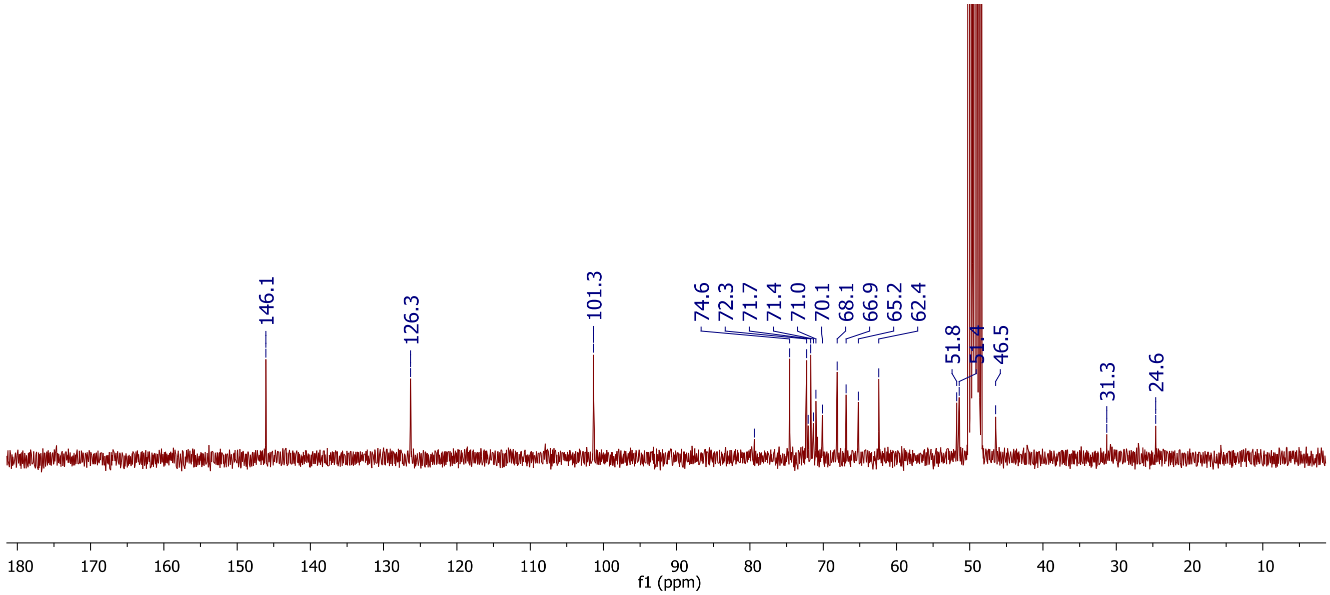

^{13}C- NMR spectrum of compound 27.

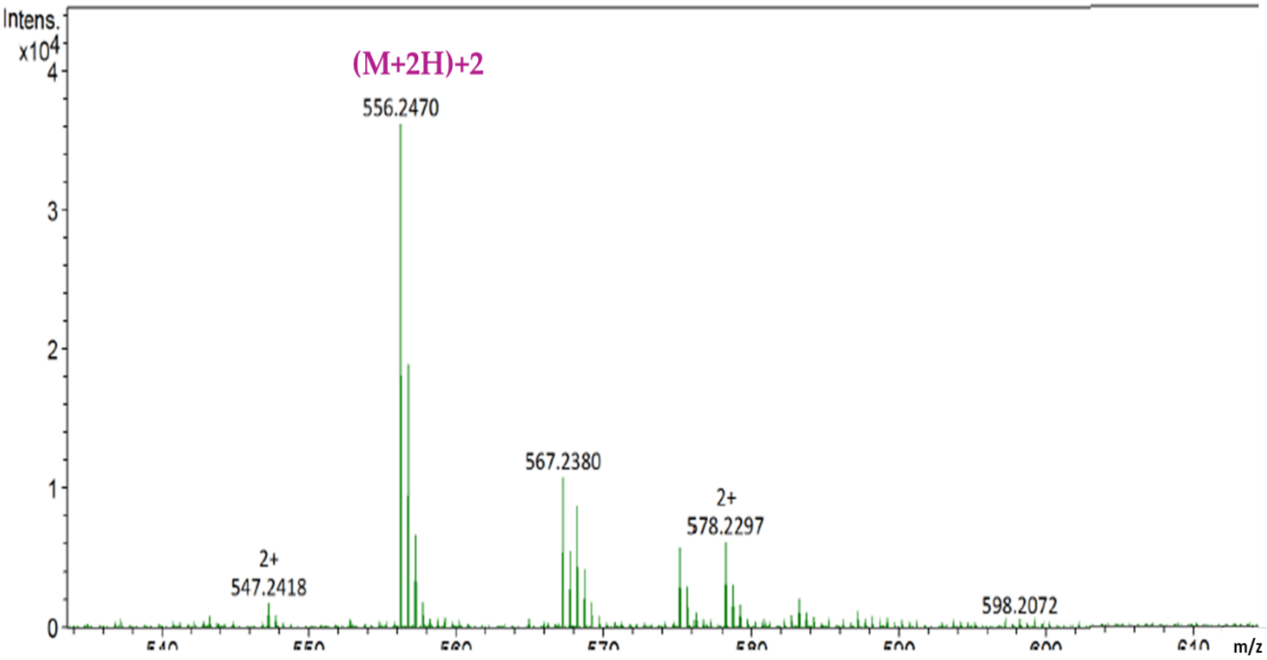

HRMS of compound 27.

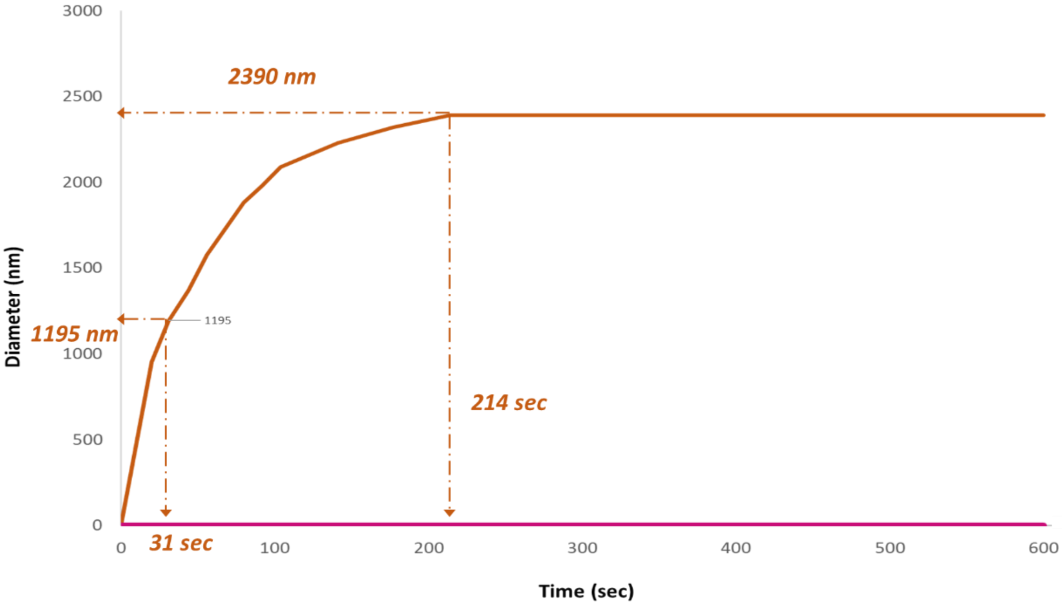

Fig 3. Synthesis DLS of Cpd **27** (Trivalent dendron) Crosslinked with Con A.

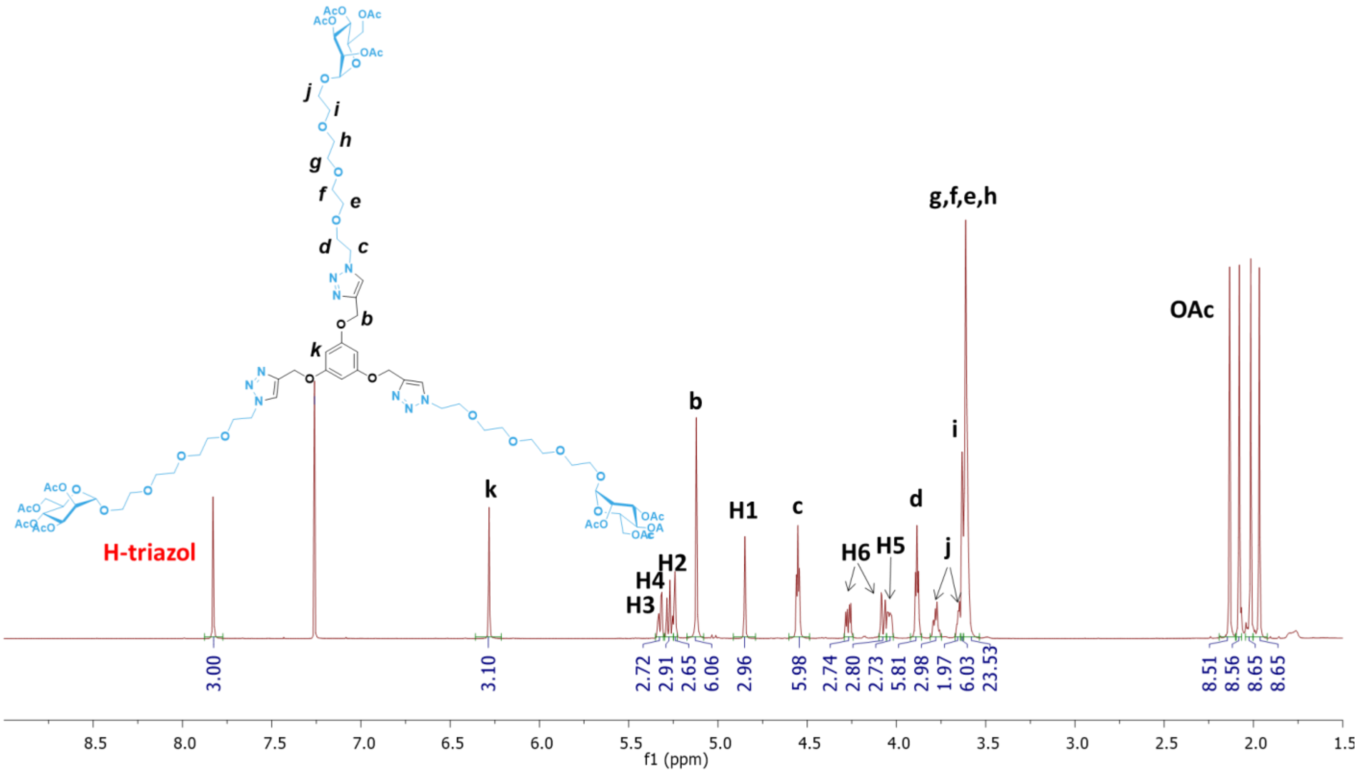

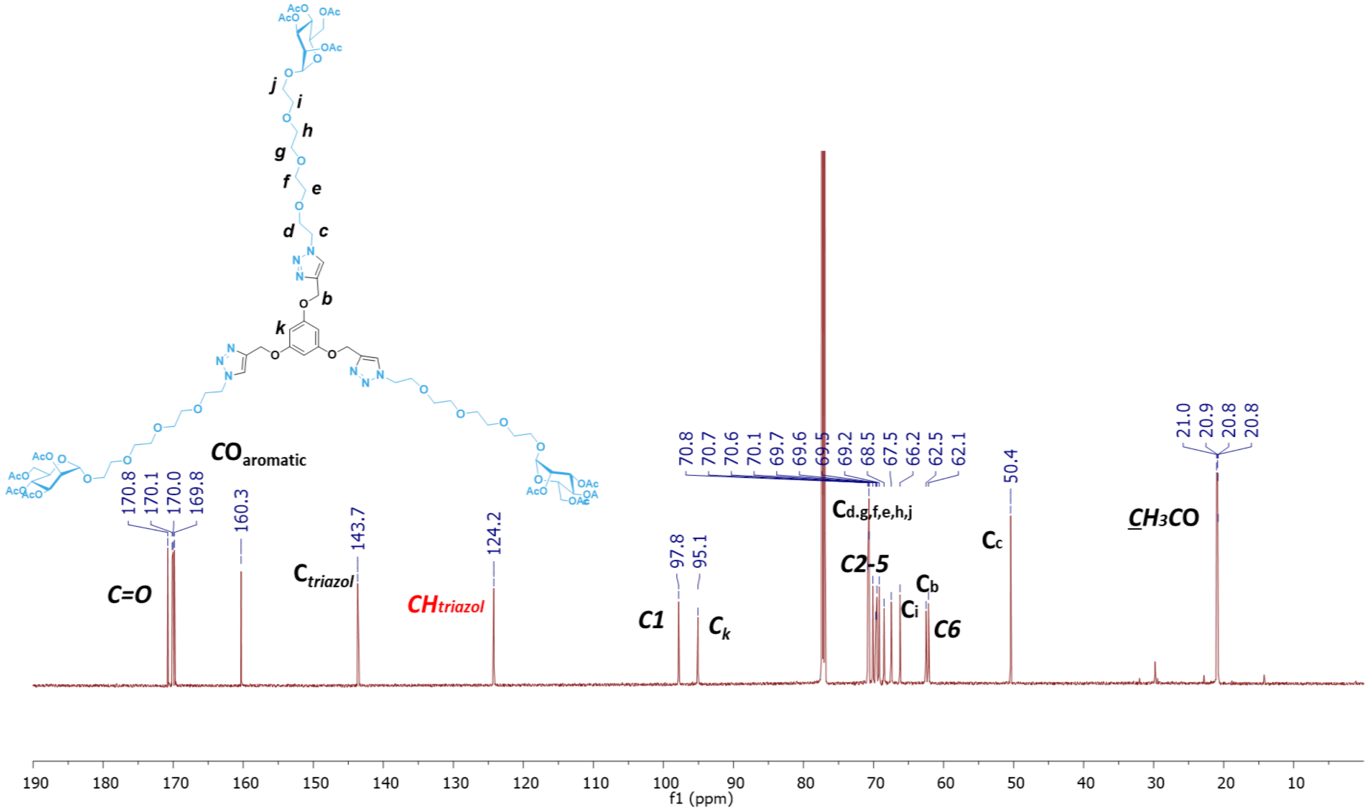

^{1}H- and ^{13}C- NMR spectrum of compound 28.

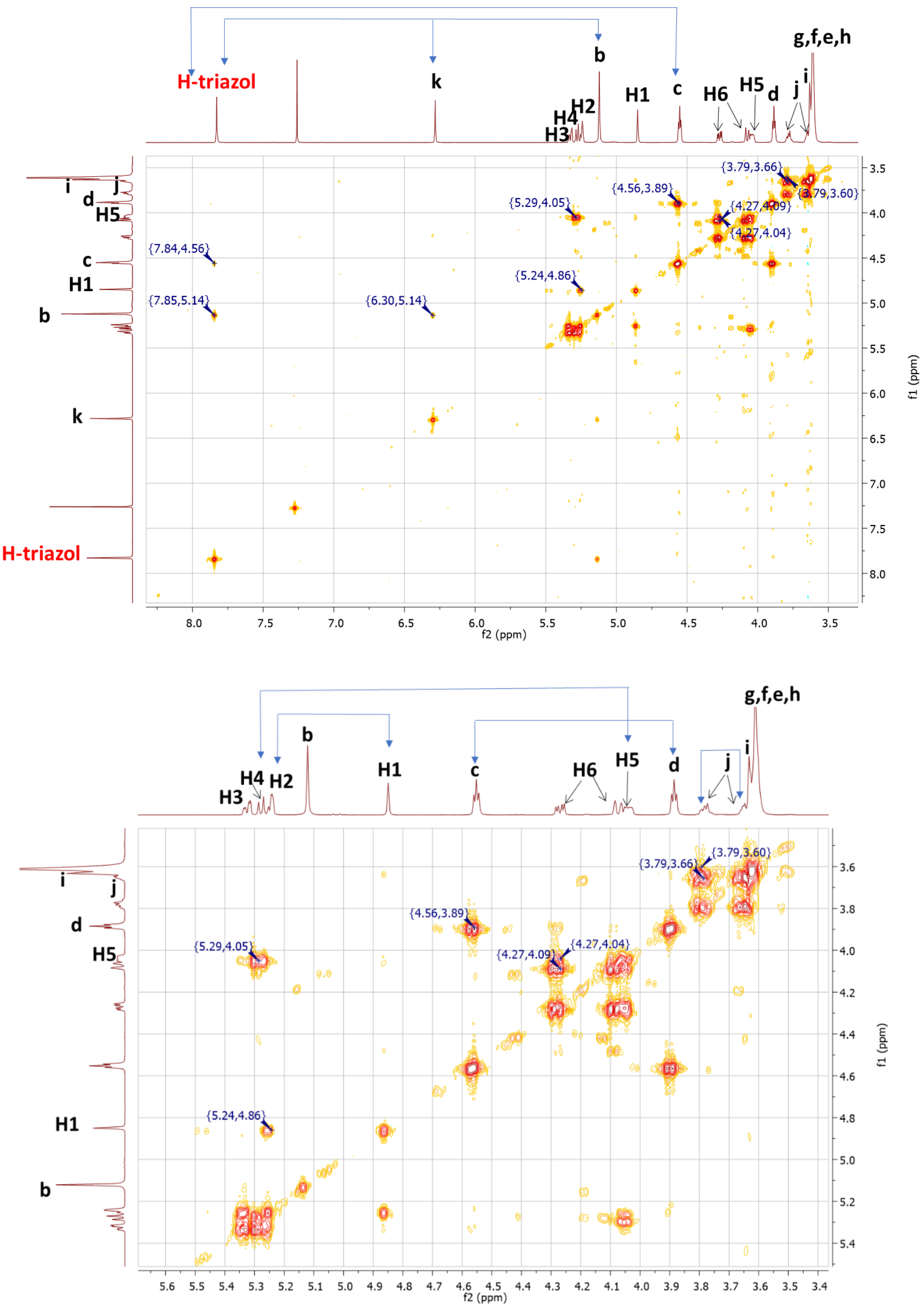

2D NMR – COSY: ^{1}H-^{1}H correlation spectrum of compound 28.

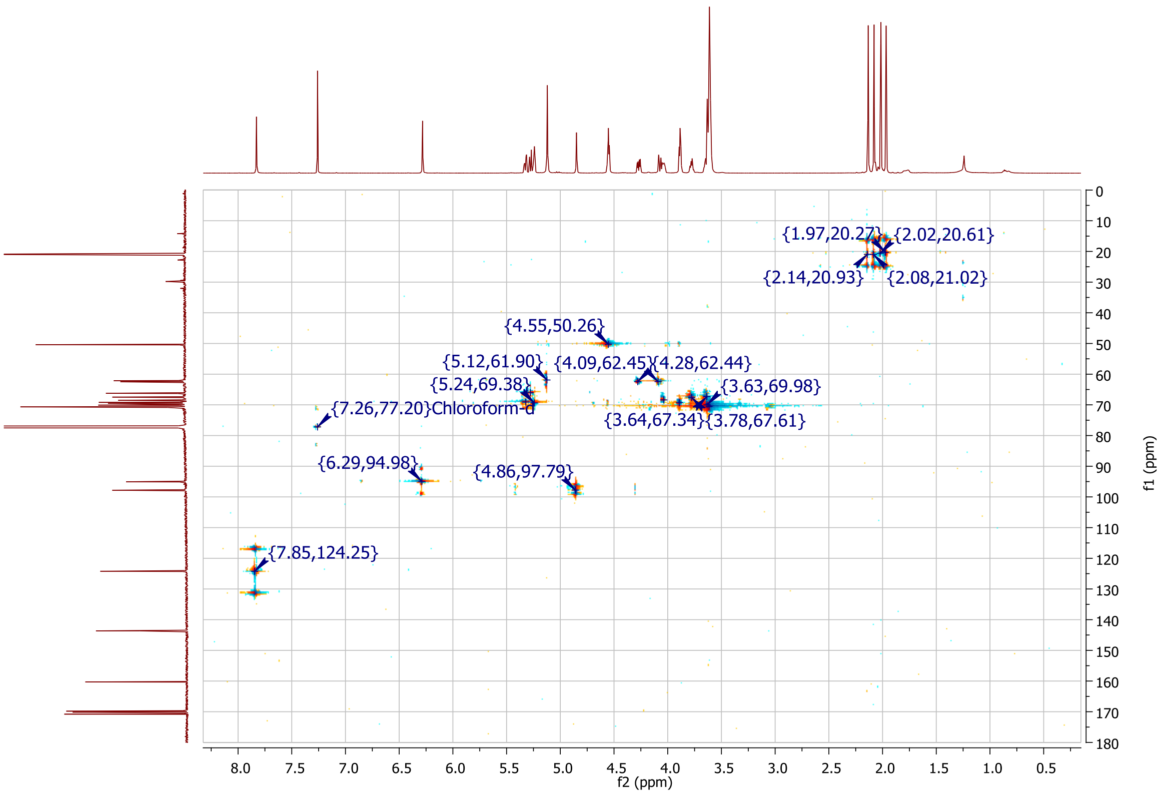

2D NMR – HSQC: ^{1}H-^{13}C correlation spectrum of compound 28.

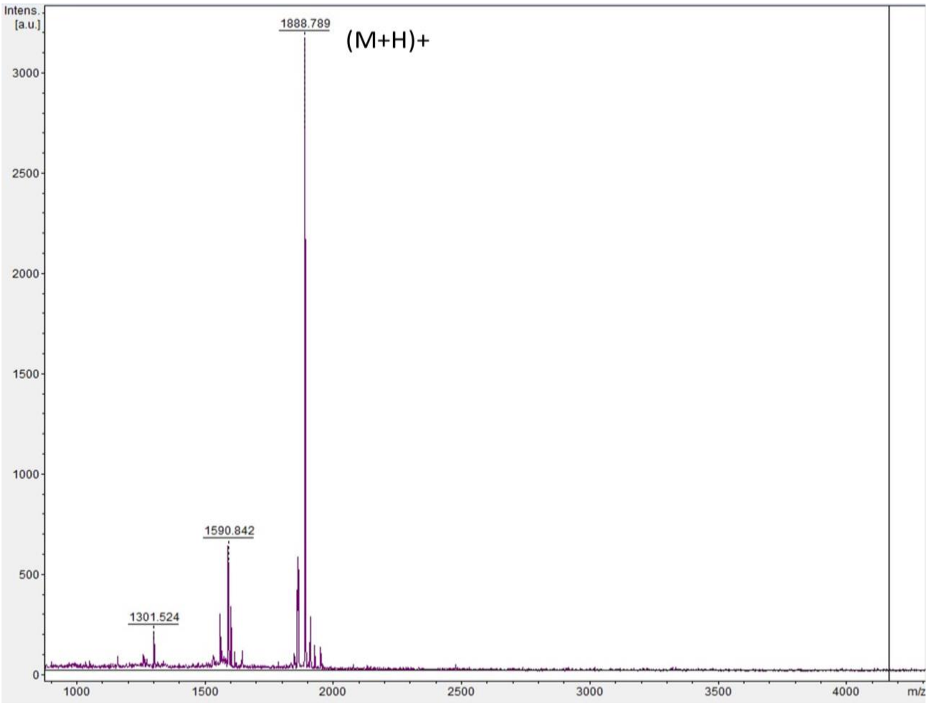

HRMS of compound 28.

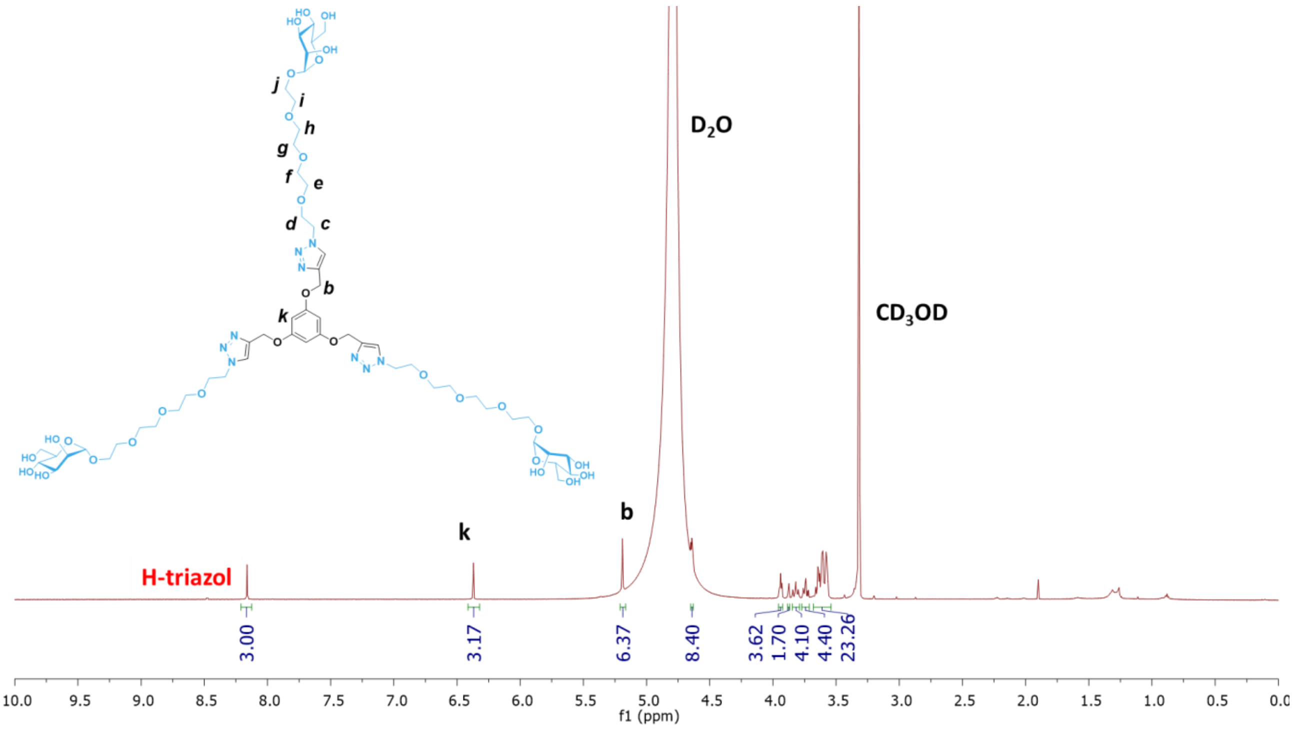

^{1}H- NMR spectrum of compound 29.

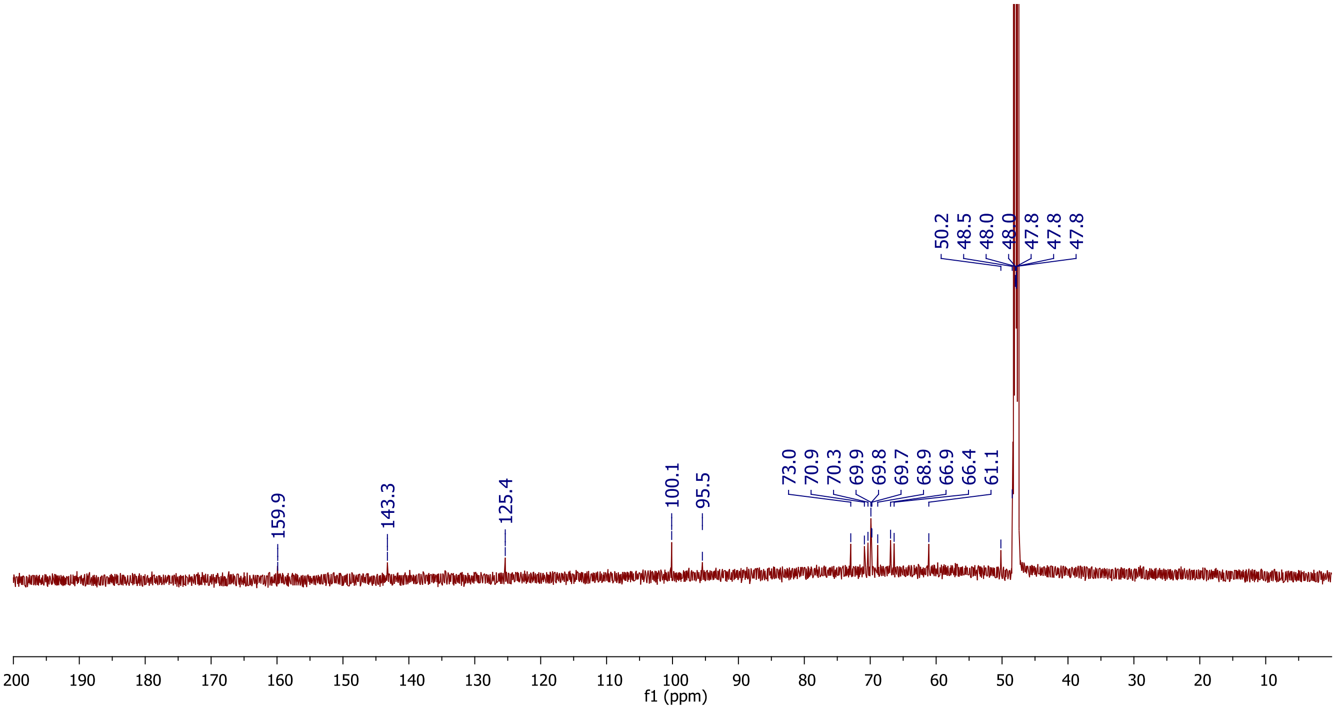

^{13}C- NMR spectrum of compound 29.

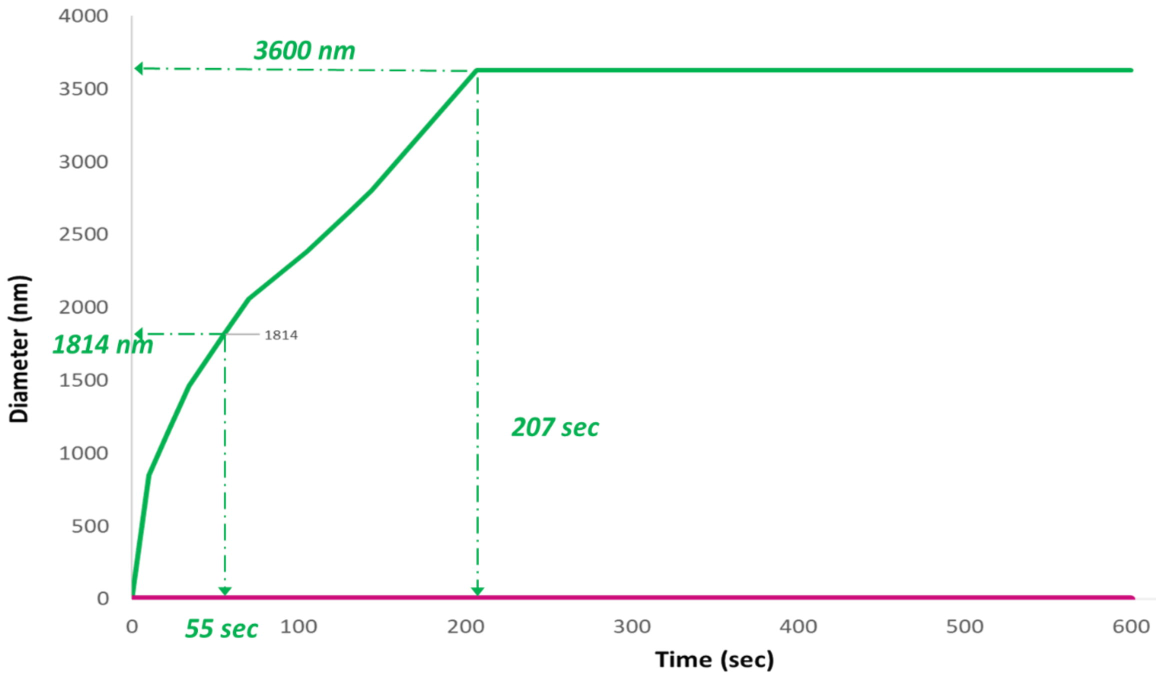

Fig 4. Synthesis DLS of Cpd **29** (Tis) Crosslinked with Con A.

			Size (d.nm):	% Number:	St Dev (d.n...
Z-Average (d.nm):	8048	Peak 1:	167.9	100.0	21.81
PdI:	0.457	Peak 2:	0.000	0.0	0.000
Intercept:	0.831	Peak 3:	0.000	0.0	0.000
Result quality :	Refer to quality report				

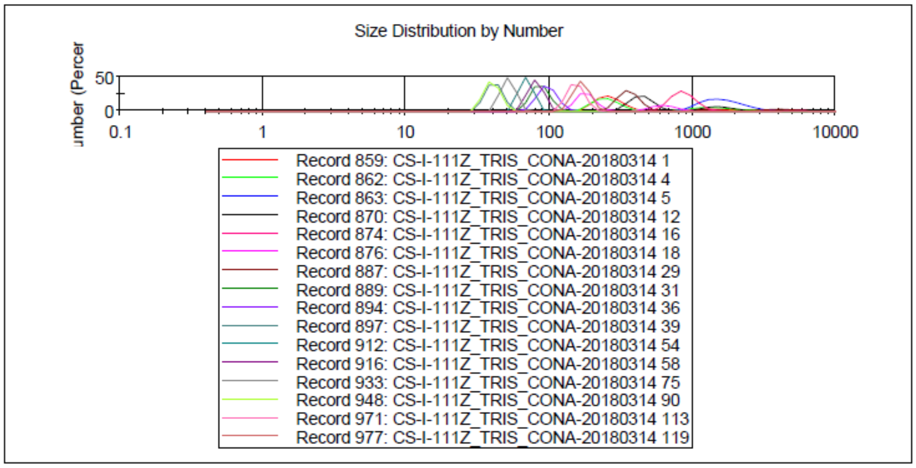

DLS-Size distribution of compound 29.

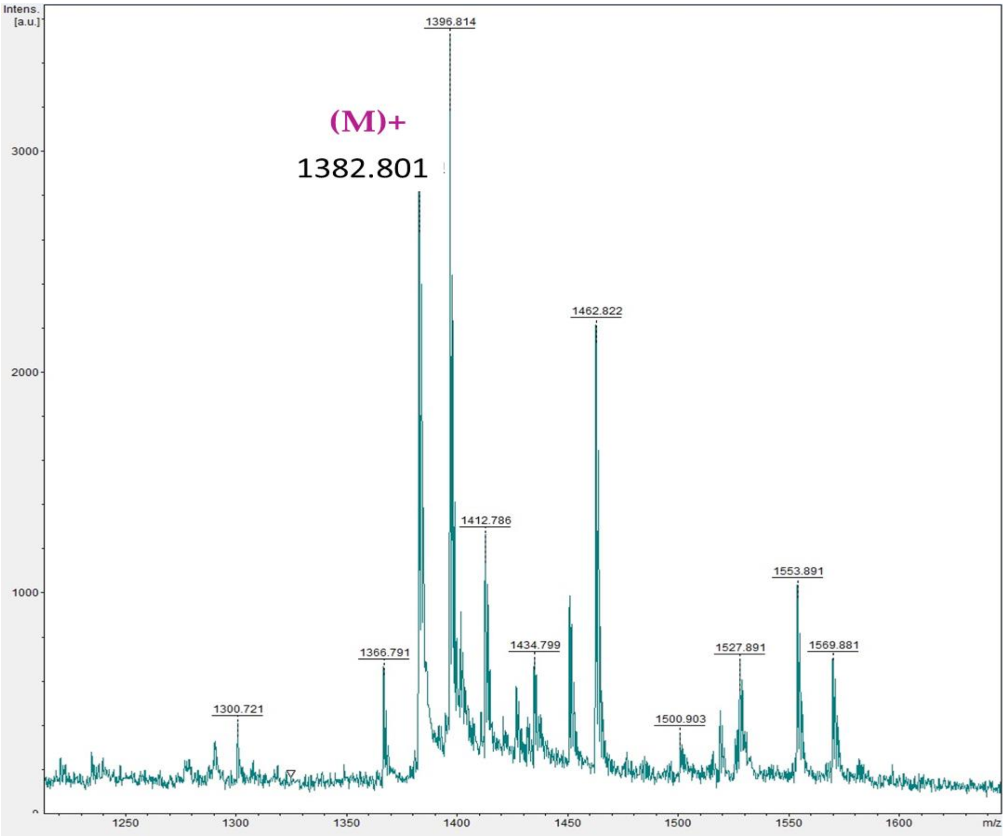

HRMS of compound 29.

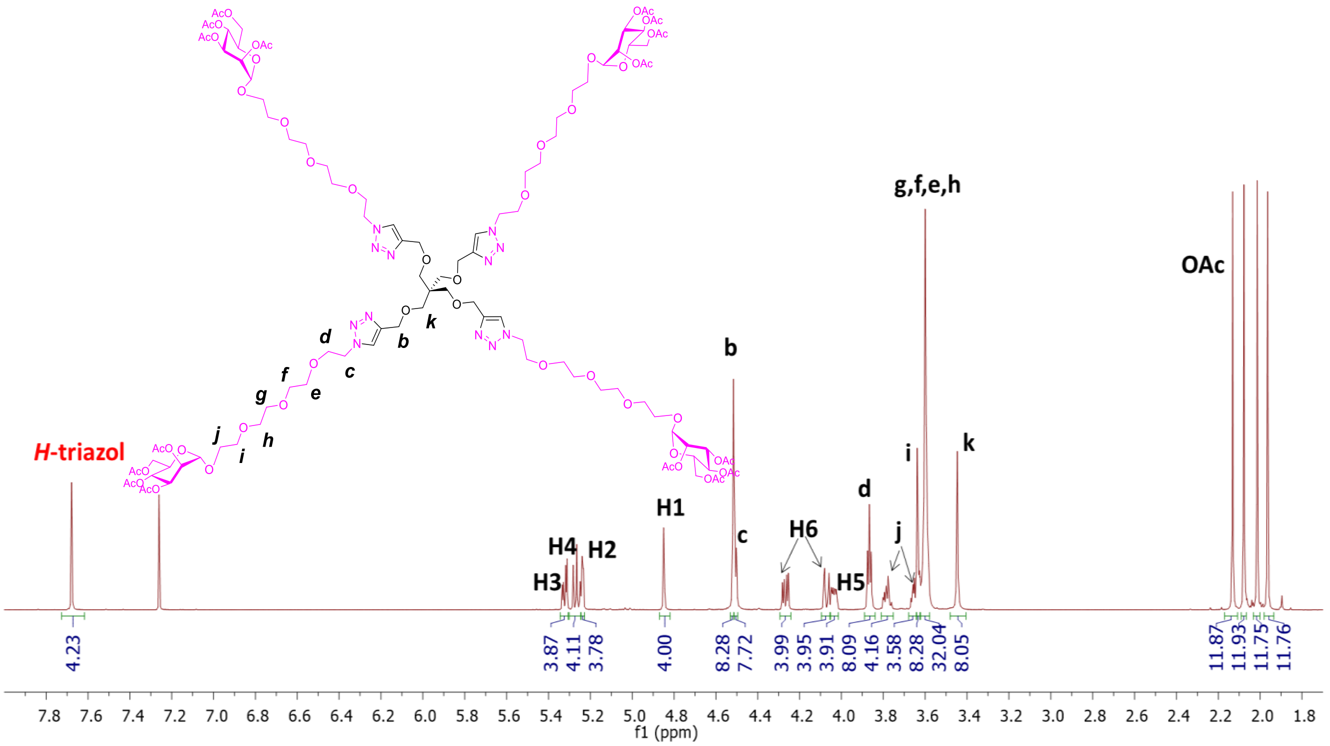

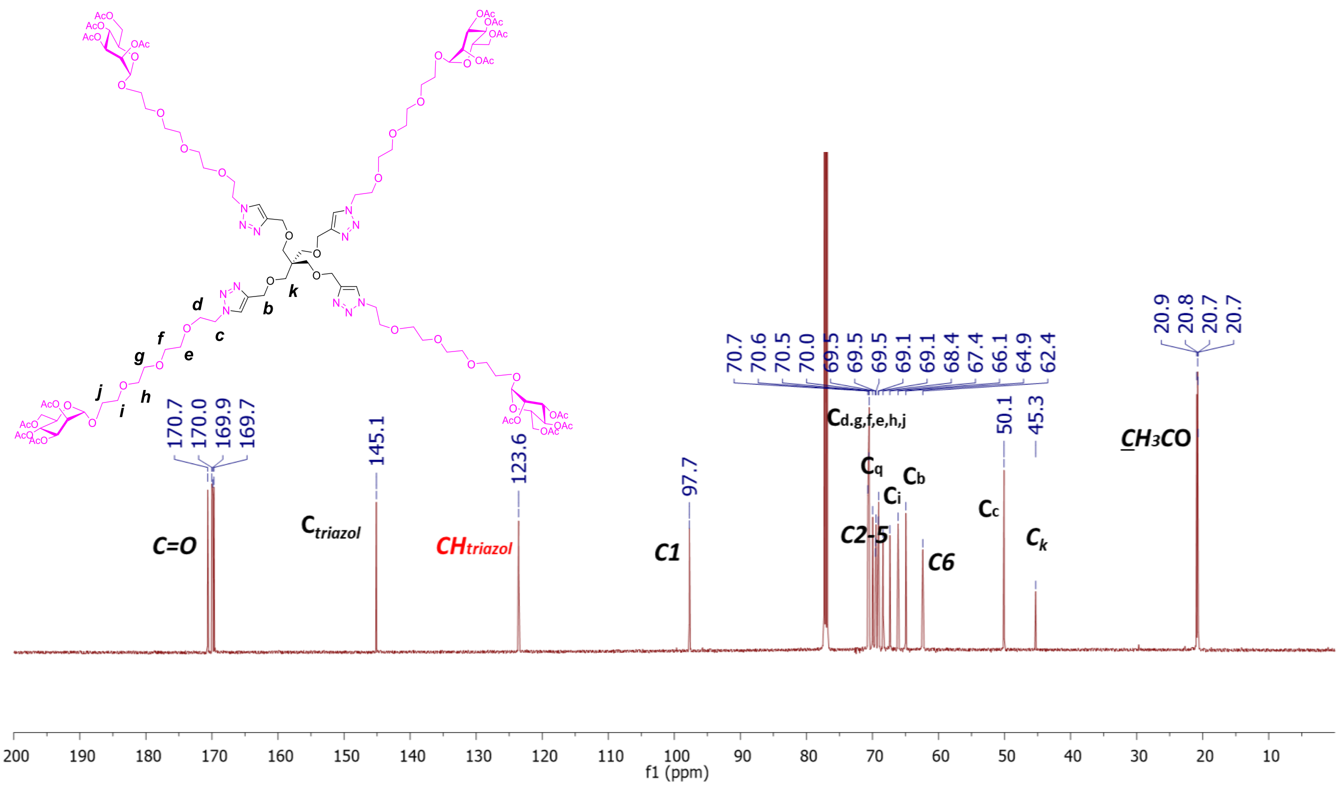

^{1}H- and ^{13}C- NMR spectrum of compound 30.

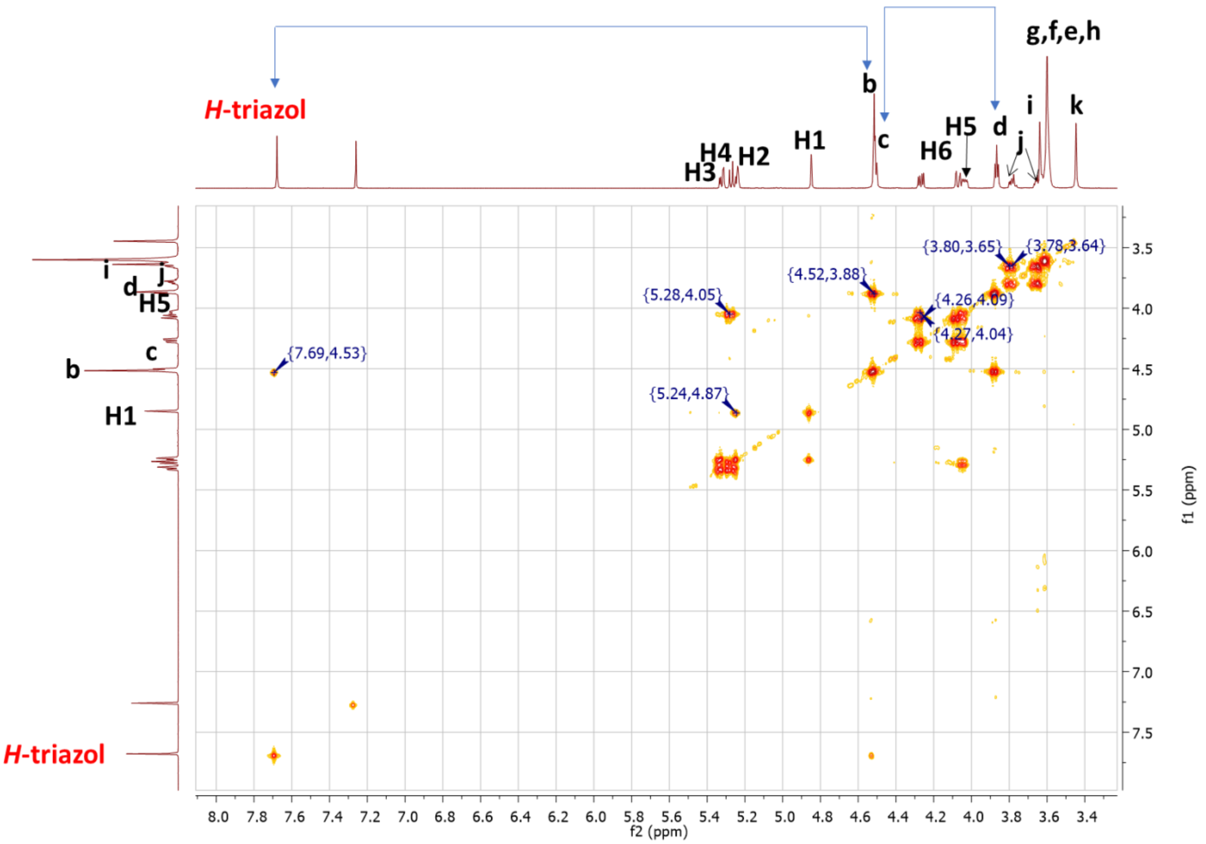

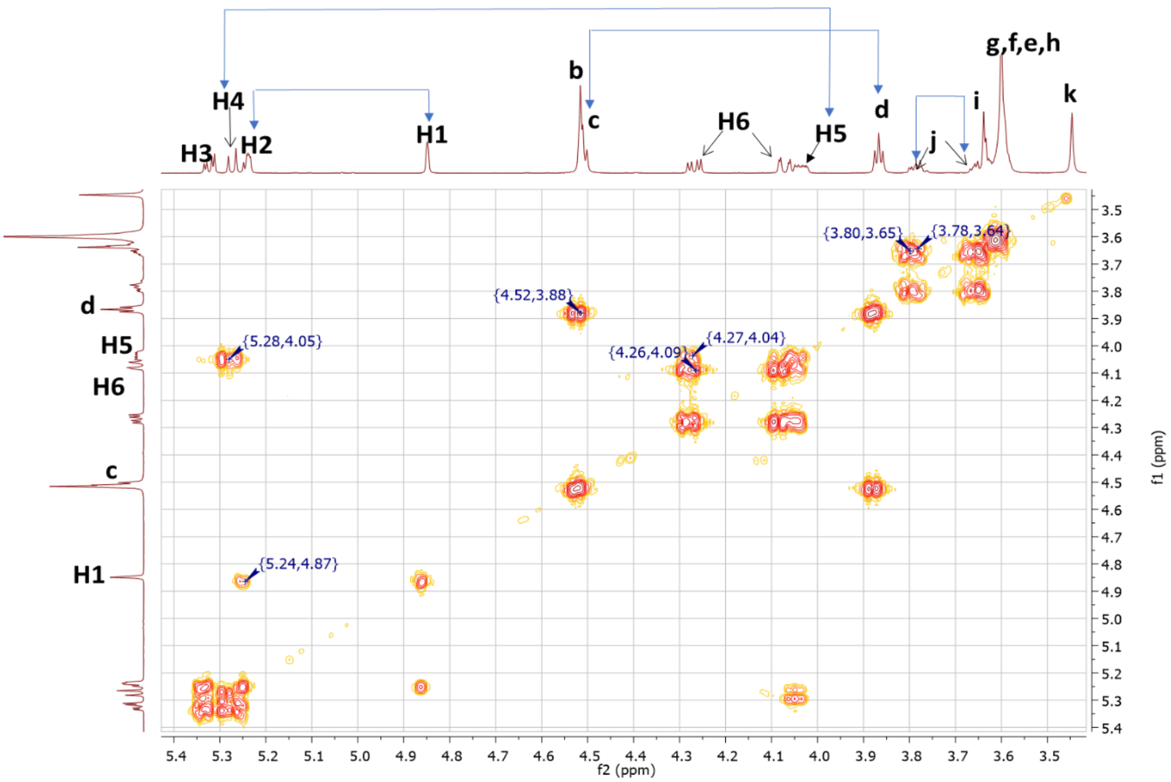

2D NMR – COSY: ^{1}H-^{1}H correlation spectrum of compound 30.

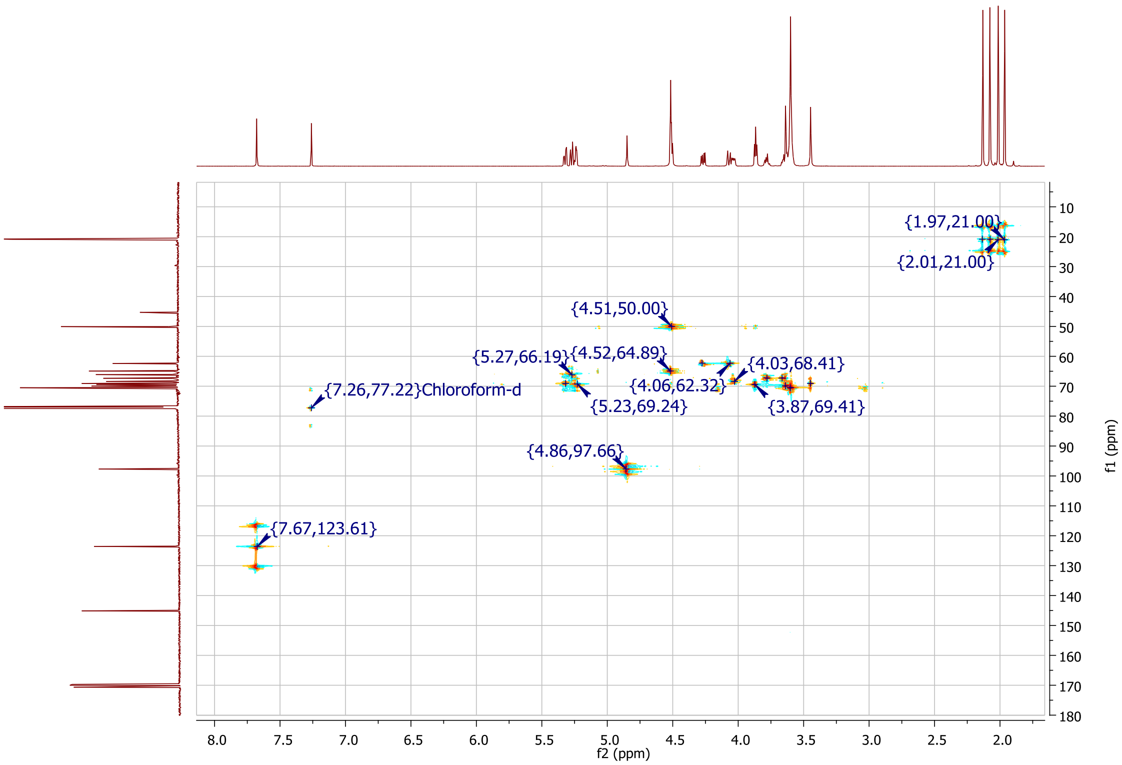

2D NMR – HSQC: ^{1}H-^{13}C correlation spectrum of compound 30.

Compound Table

Compound Label	RT	Mass	Abund	Formula	Tgt Mass	Diff (ppm)
Cpd 1: C105 H160 N12 O56	0.1	2485.008	9337	C105 H160 N12 O56	2485.0041	1.58

Compound Label	RT	Algorithm	Mass
Cpd 1: C105 H160 N12 O56	0.1	Find By Formula	2485.008

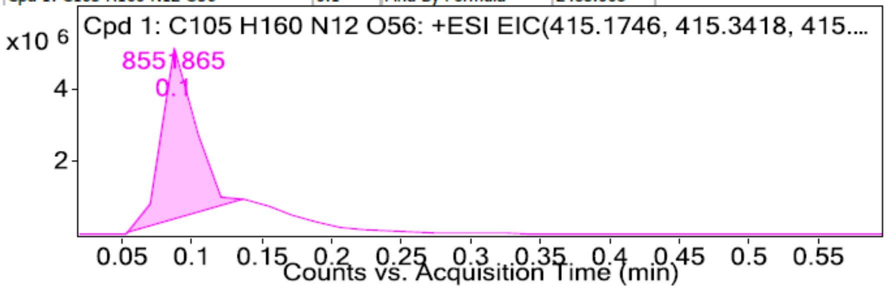

MS Spectrum

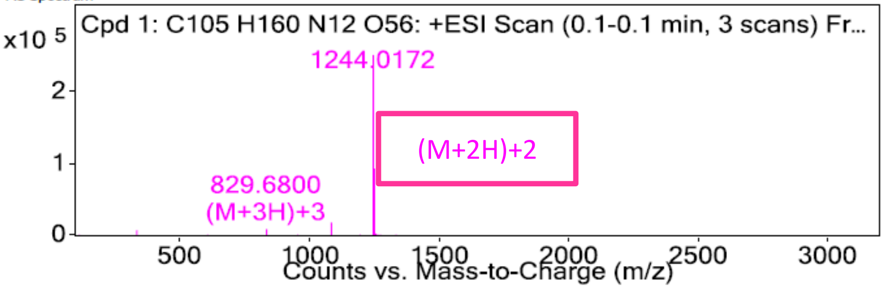

HRMS of compound 30.

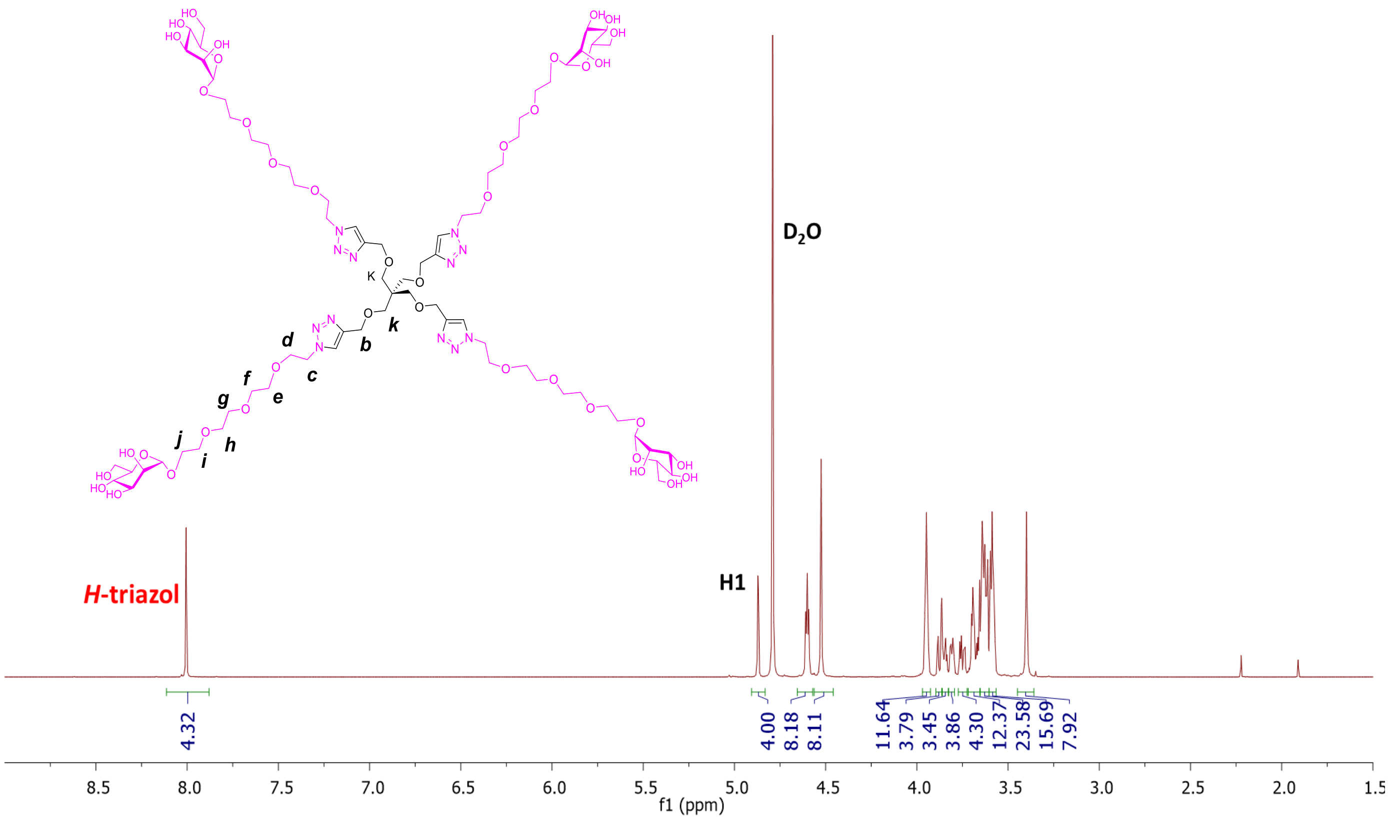

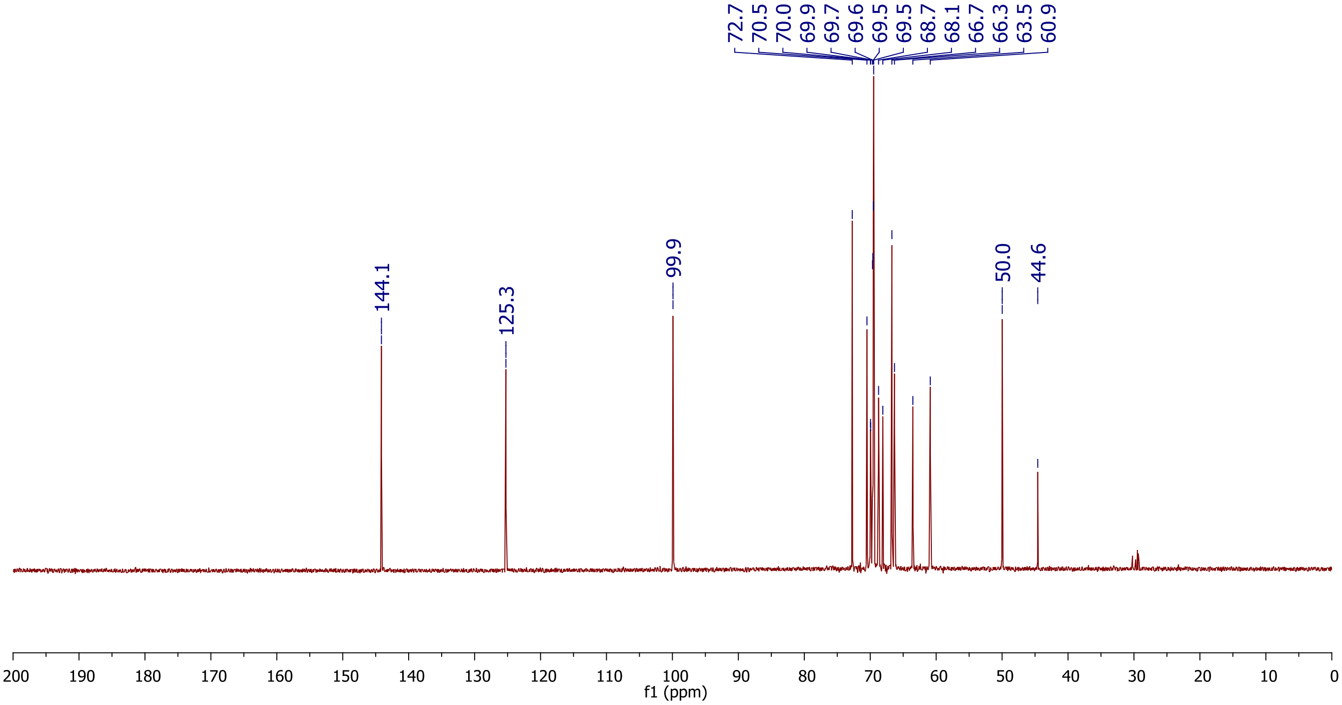

^{1}H- and ^{13}C- NMR spectrum of compound 31.

Compound Table

Compound Label	RT	Mass	Abund	Formula	Tgt Mass	Diff (ppm)
Cpd 1: C73 H128 N12 O40	0.1	1812.8362	28364	C73 H128 N12 O40	1812.8351	0.6

Compound Label	RT	Algorithm	Mass
Cpd 1: C73 H128 N12 O40	0.1	Find By Formula	1812.8362

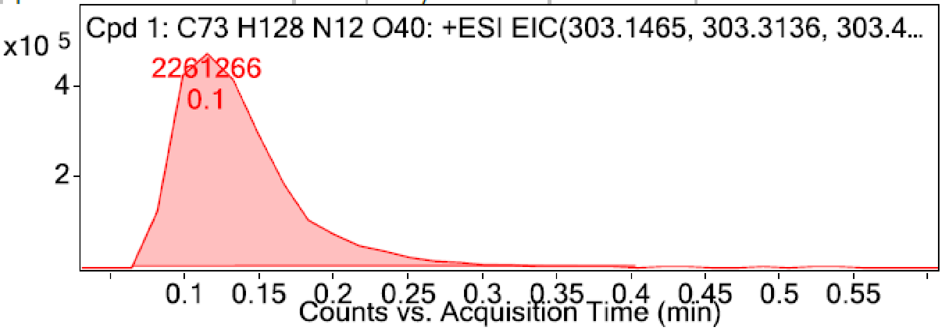

MS Spectrum

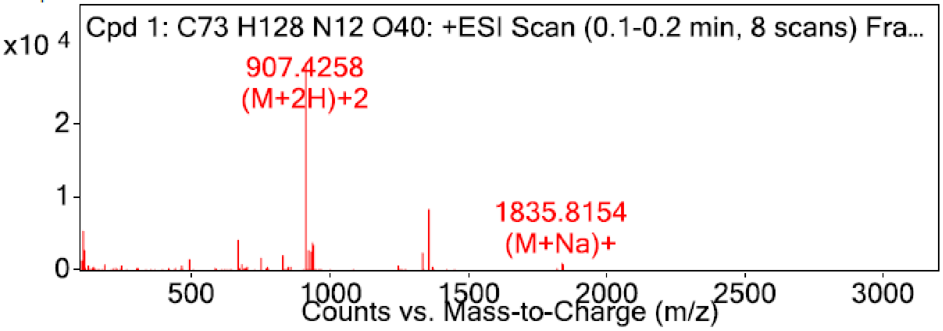

HRMS of compound 31.

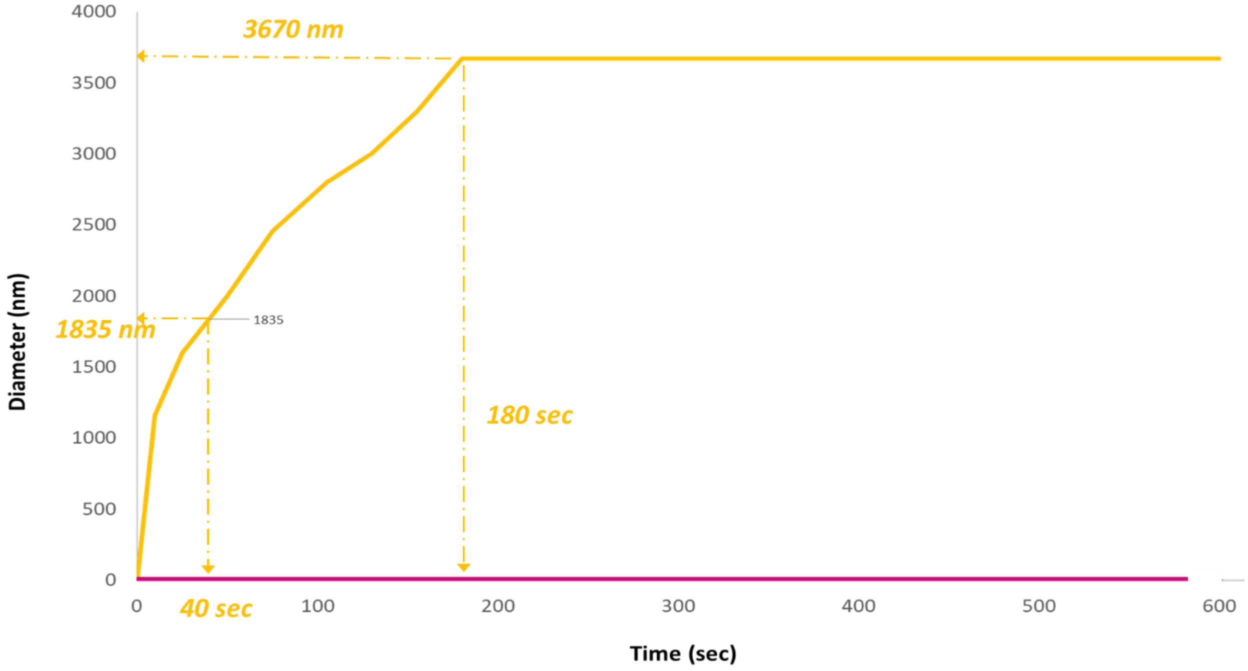

Fig 5. Synthesis DLS of Cpd **31** (Tetra) Crosslinked with Con A.

Results

			Size (d.nm):	% Number:	St Dev (d.n...
Z-Average (d.nm):	5235	Peak 1:	5051	100.0	747.9
PdI:	0.030	Peak 2:	0.000	0.0	0.000
Intercept:	0.894	Peak 3:	0.000	0.0	0.000

Result quality : Refer to quality report

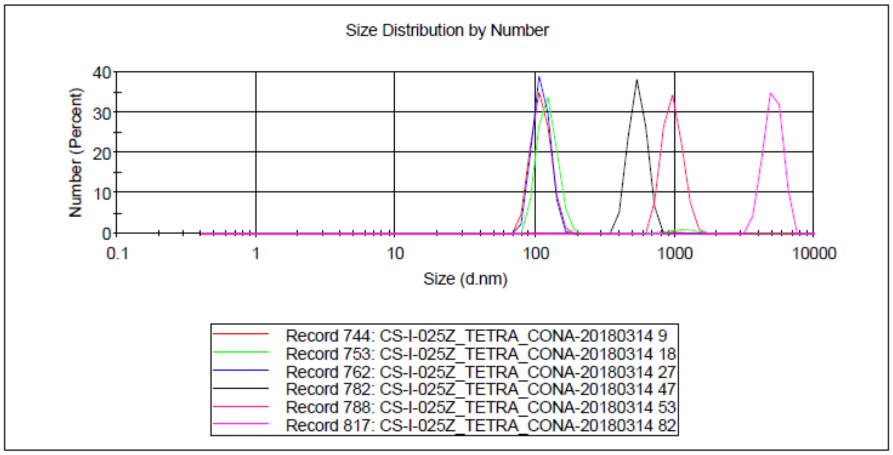

DLS-Size distribution of compound 31.

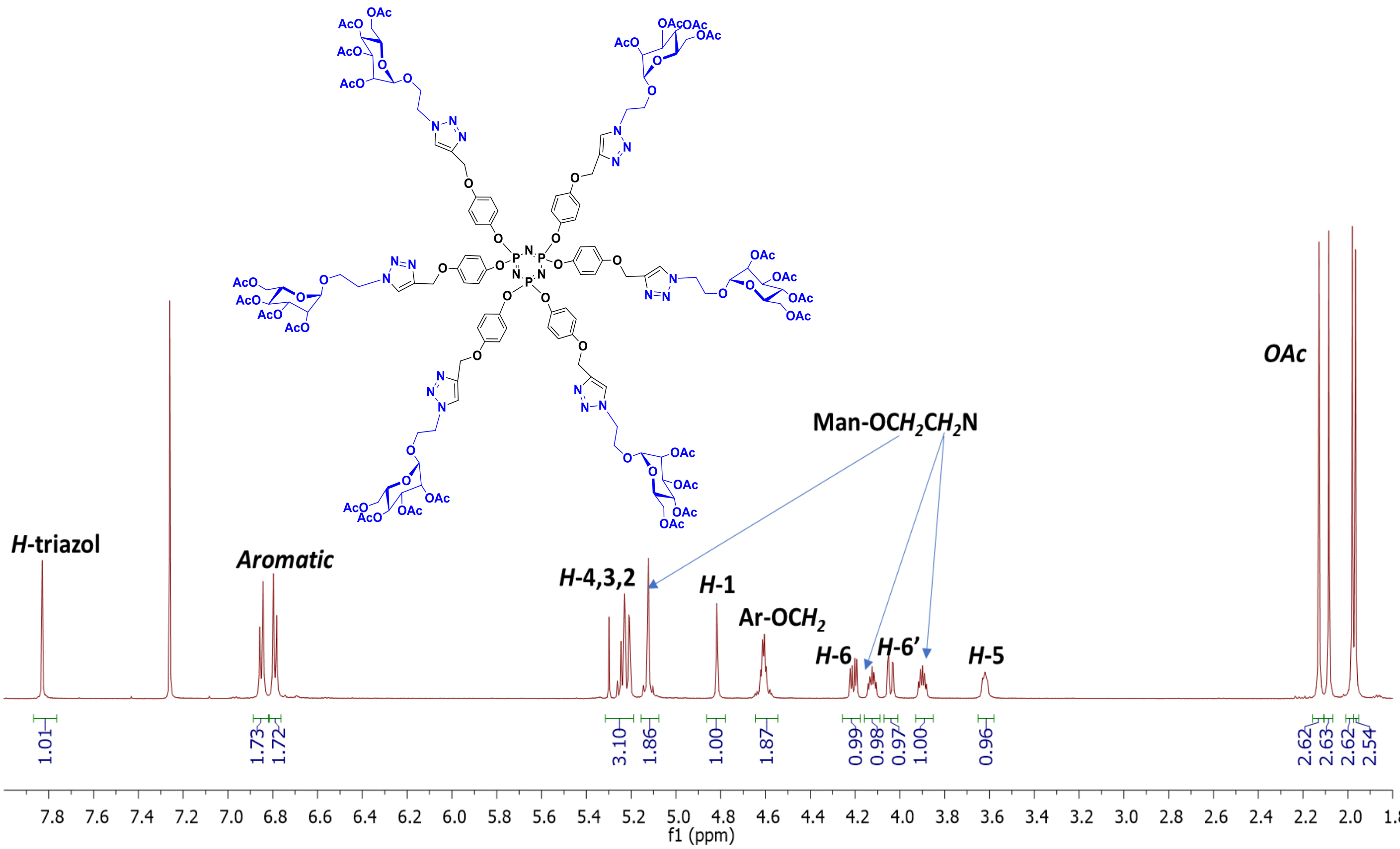

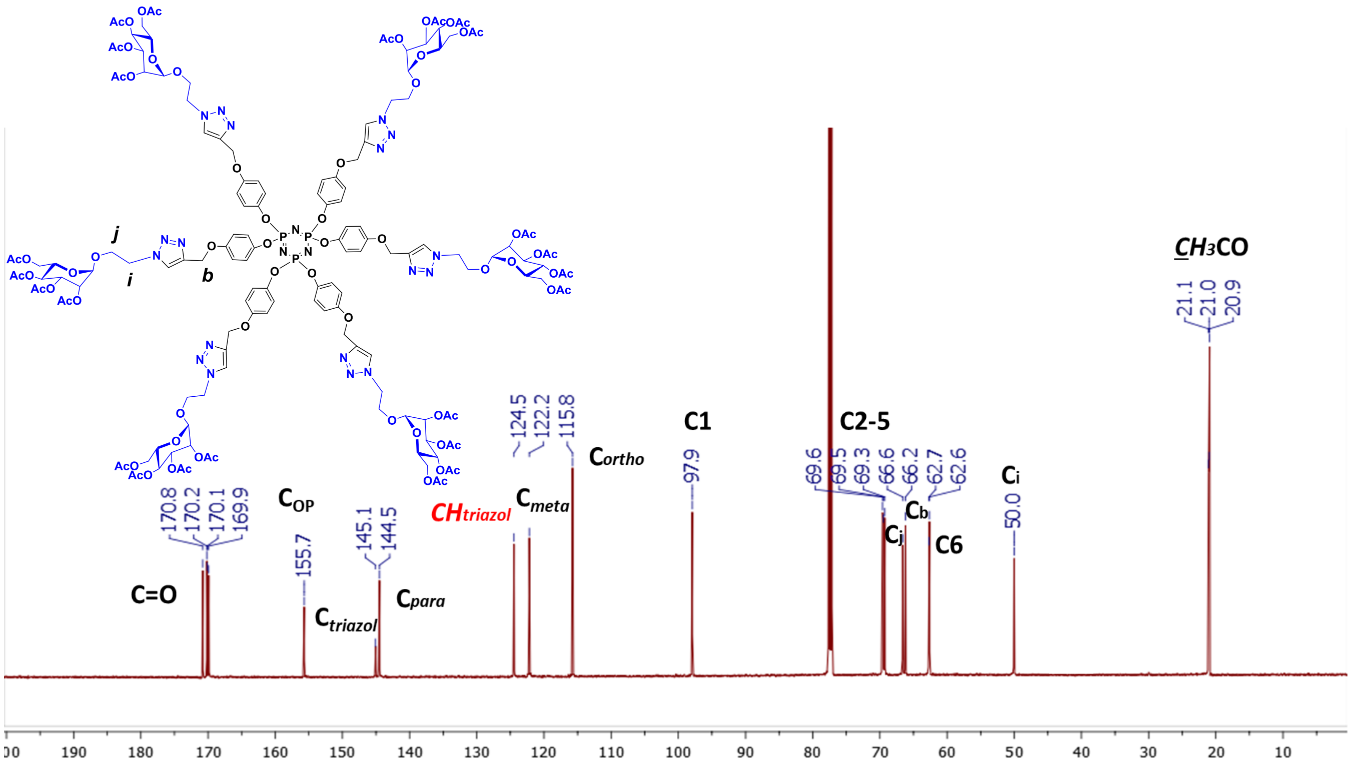

^{1}H- and ^{13}C- NMR spectrum of compound 33

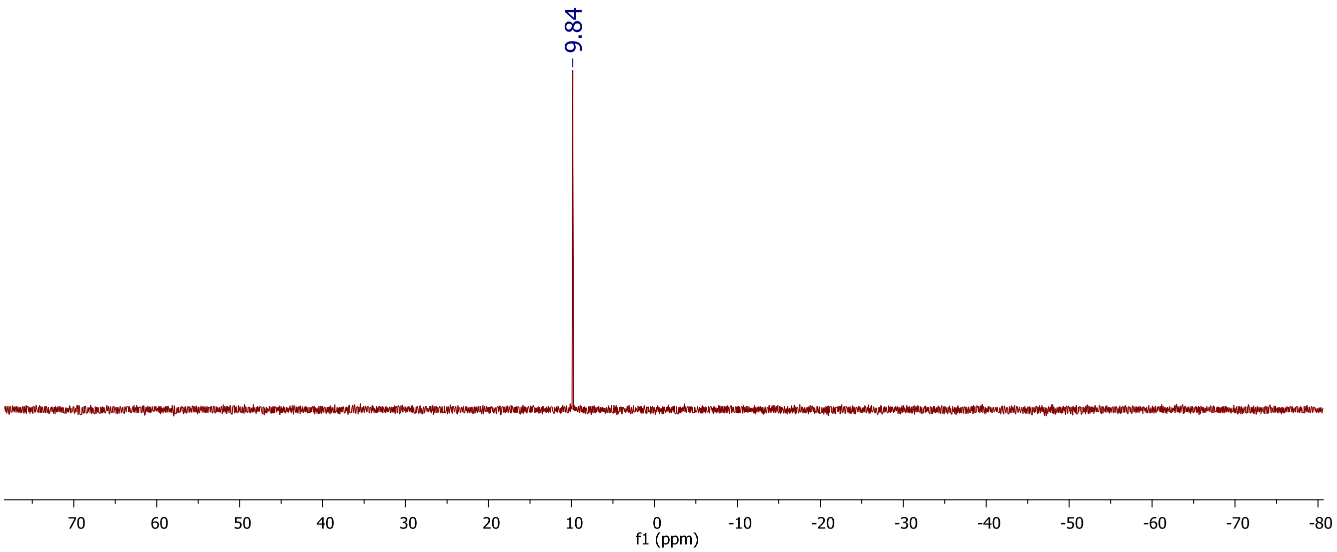

^{31}P- NMR spectrum of compound 33.

Compound Table

Compound Label	RT	Mass	Abund	Formula	Tgt Mass	Diff (ppm)
Cpd 1: C150 H180 N21 O72 P3	7.7	3520.0191	167714	C150 H180 N21 O72 P3	3520.0282	-2.59

Compound Label	RT	Algorithm	Mass
Cpd 1: C150 H180 N21 O72 P3	7.7	Find By Formula	3520.0191

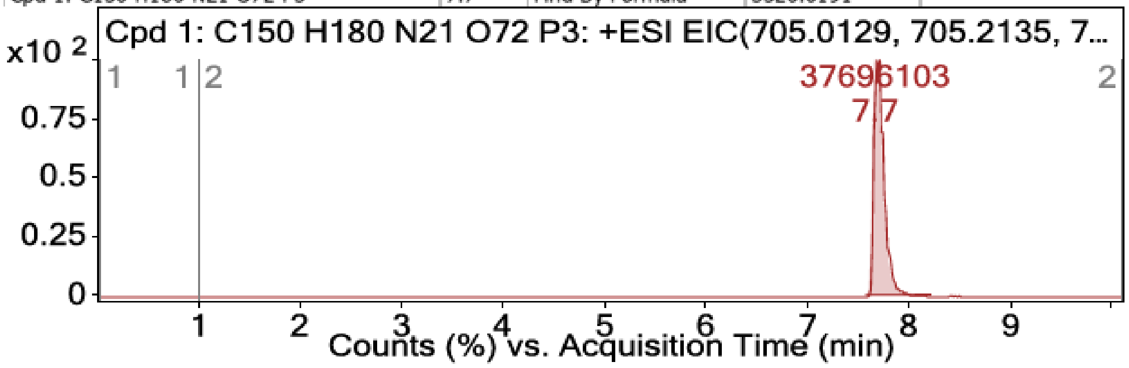

MS Spectrum

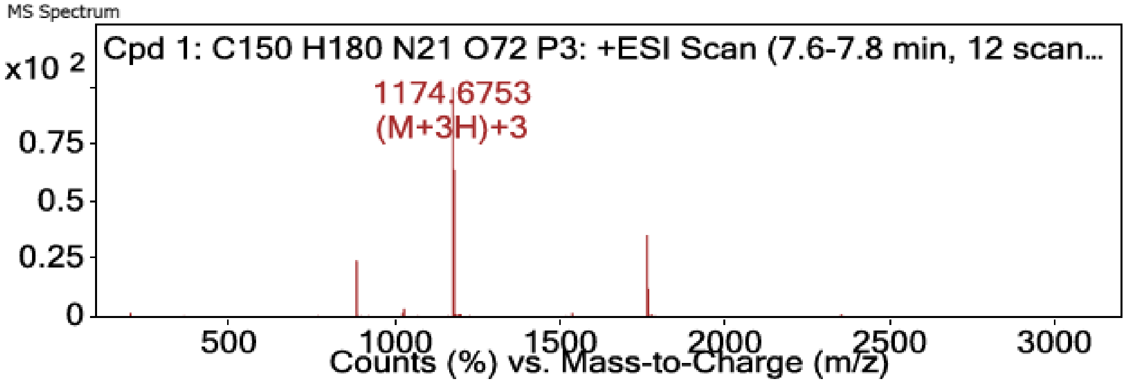

HRMS of compound 33.

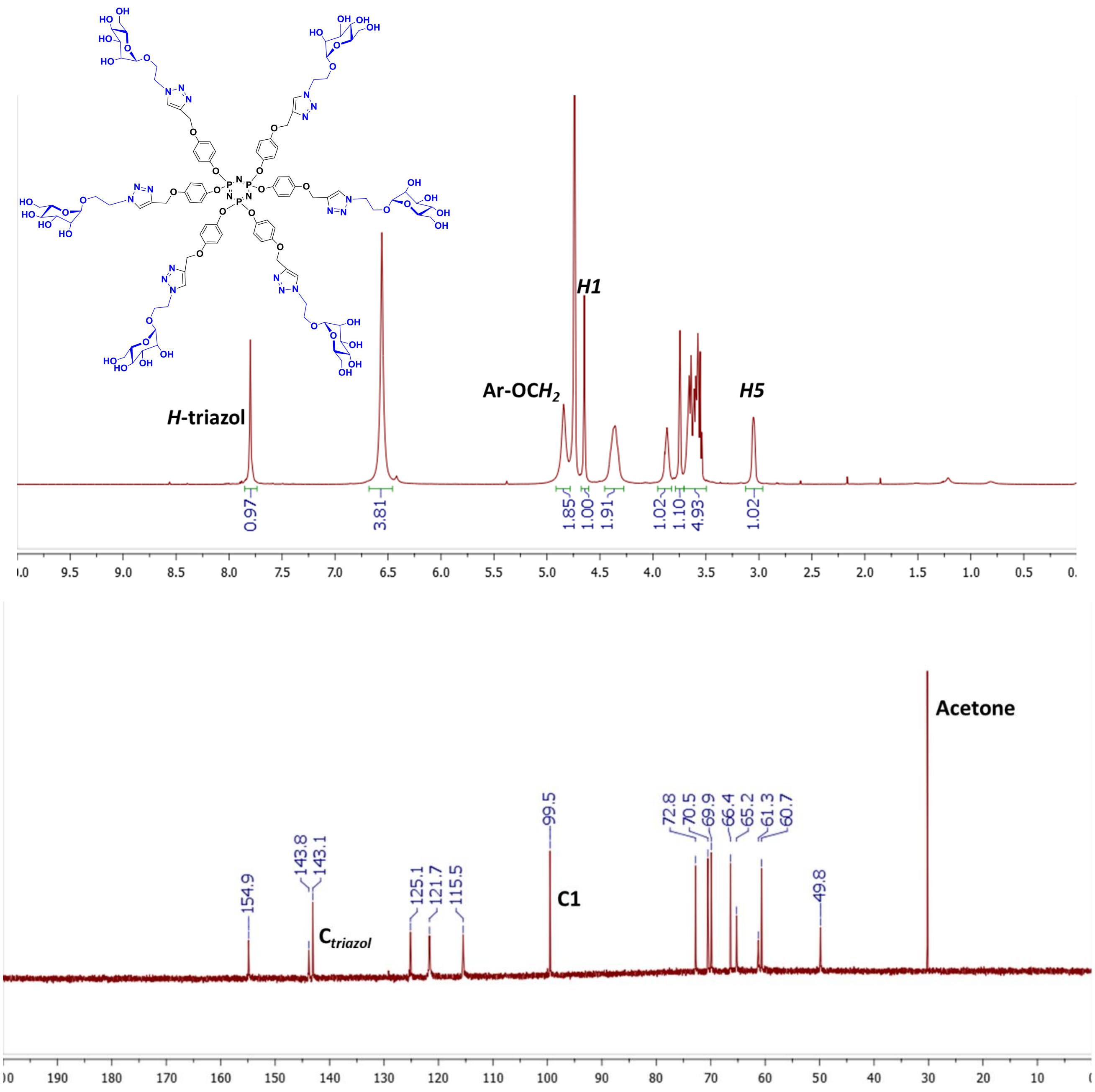

^{1}H- and ^{13}C- NMR spectrum of compound 34.

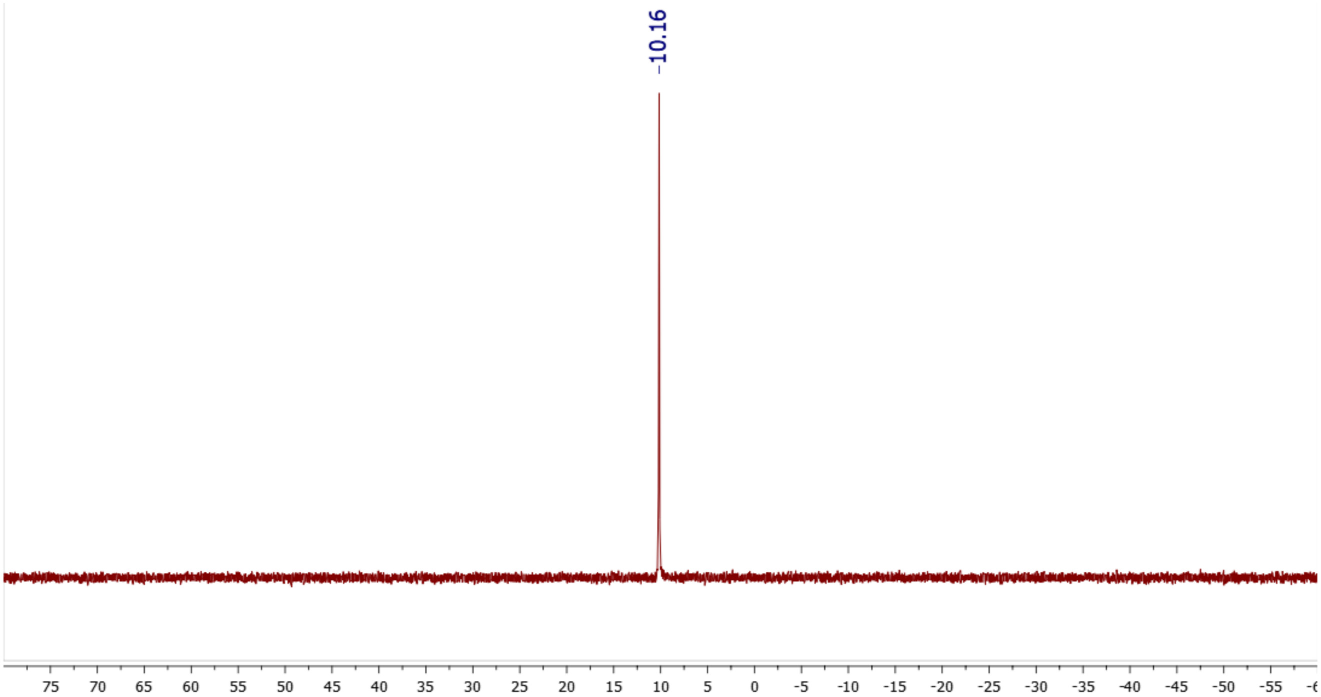

^{31}P- NMR spectrum of compound 3

Compound Table

Compound Label	RT	Mass	Abund	Formula	Tgt Mass	Diff (ppm)
Cpd 1: C102 H132 N21 O48 P3	5.6	2511.7628	48829	C102 H132 N21 O48 P3	2511.7746	-4.71

Compound Label	RT	Algorithm	Mass
Cpd 1: C102 H132 N21 O48 P3	5.6	Find By Formula	2511.7628

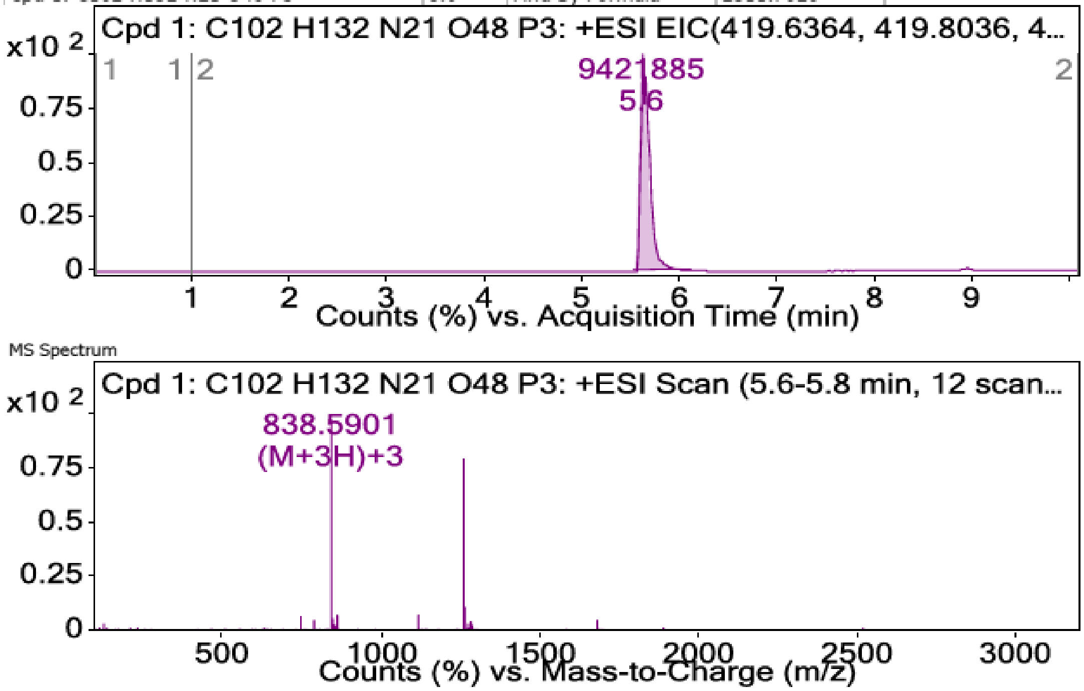

HRMS of compound 34.

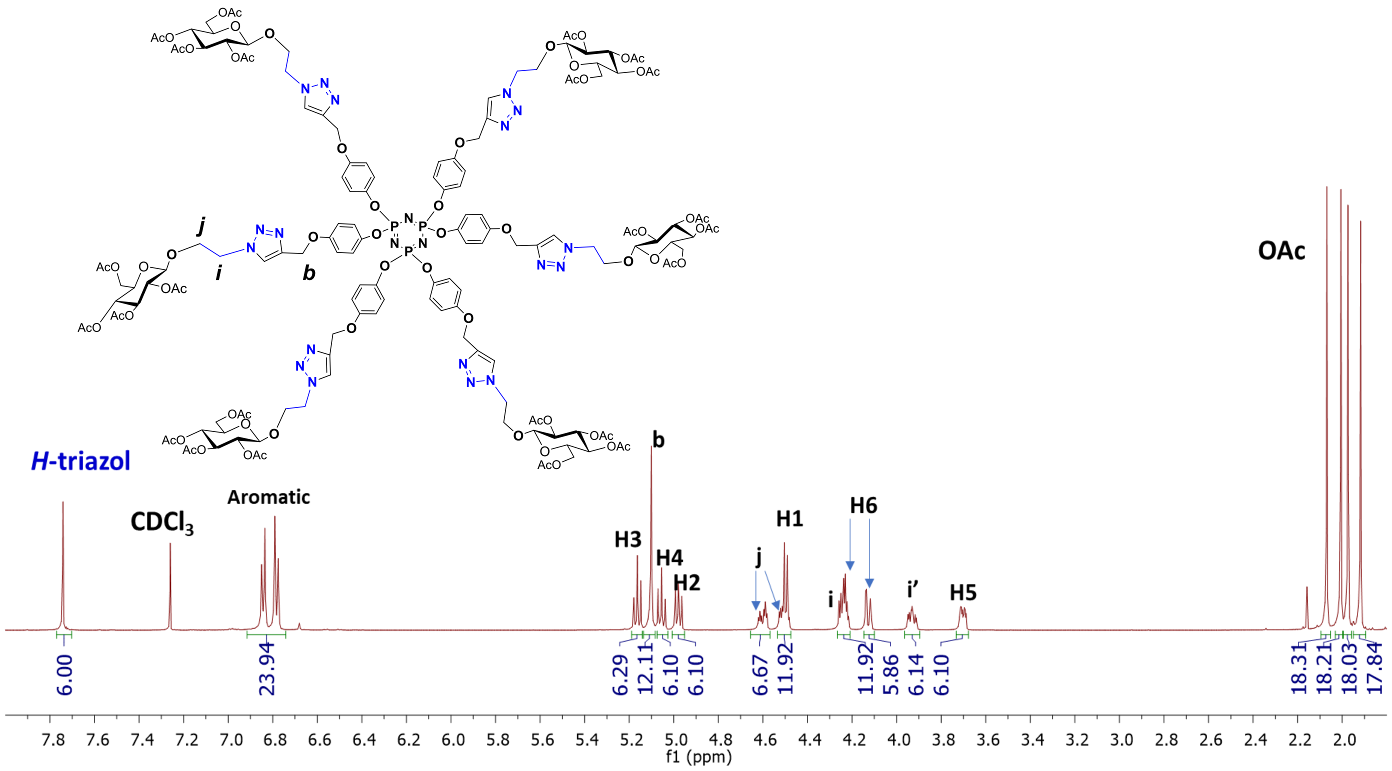

^{1}H- NMR spectrum of compound 35.

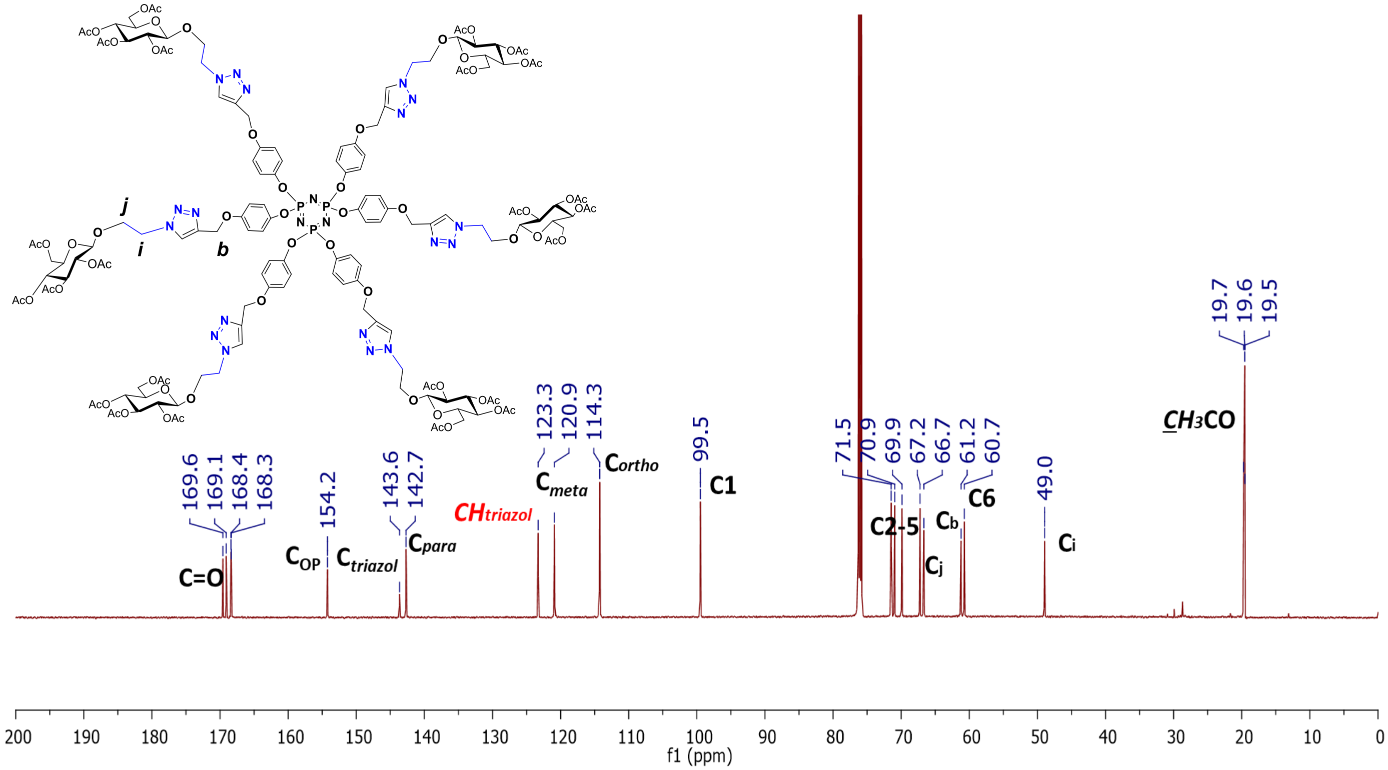

^{13}C- NMR spectrum of compound 35.

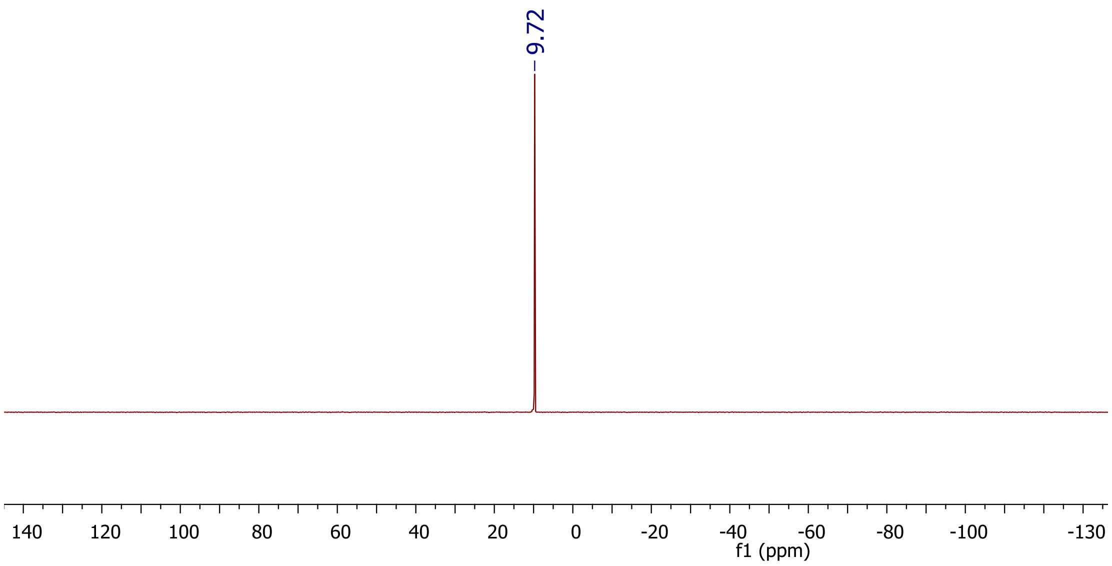

^{31}P- NMR spectrum of compound 35.

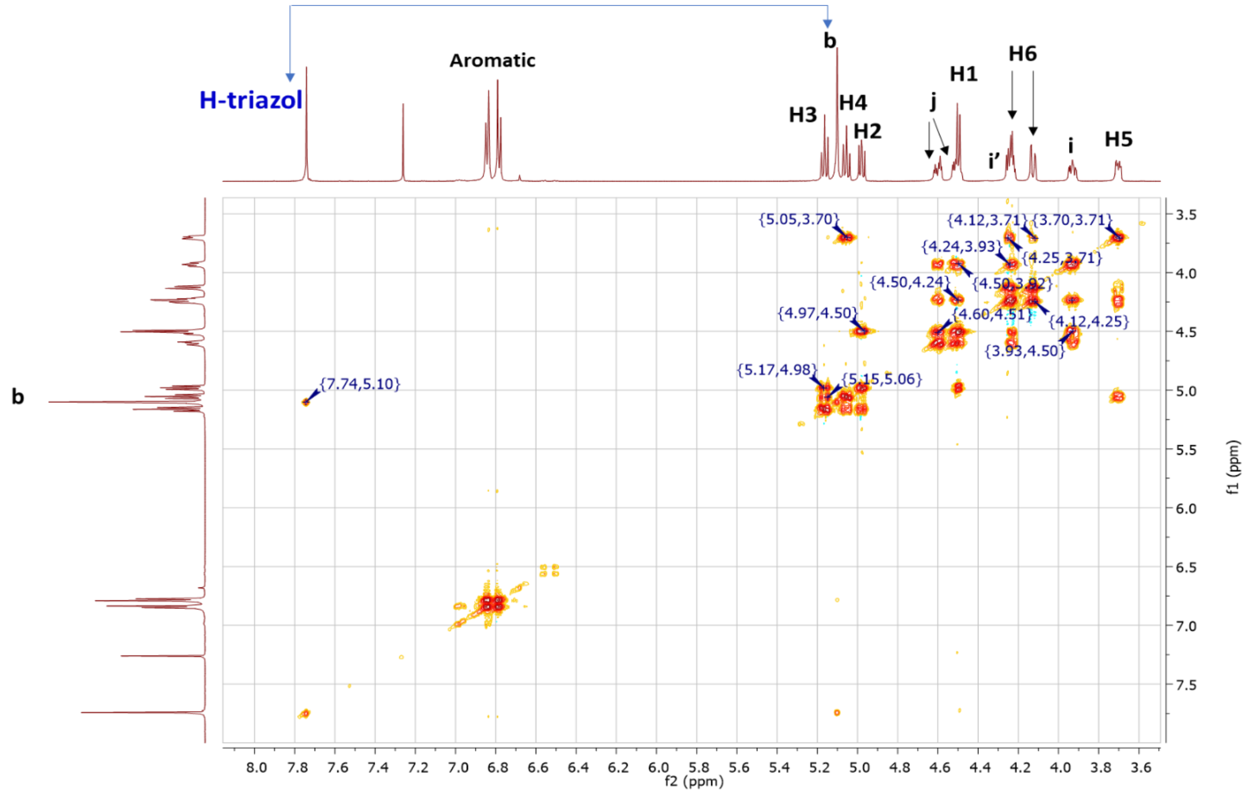

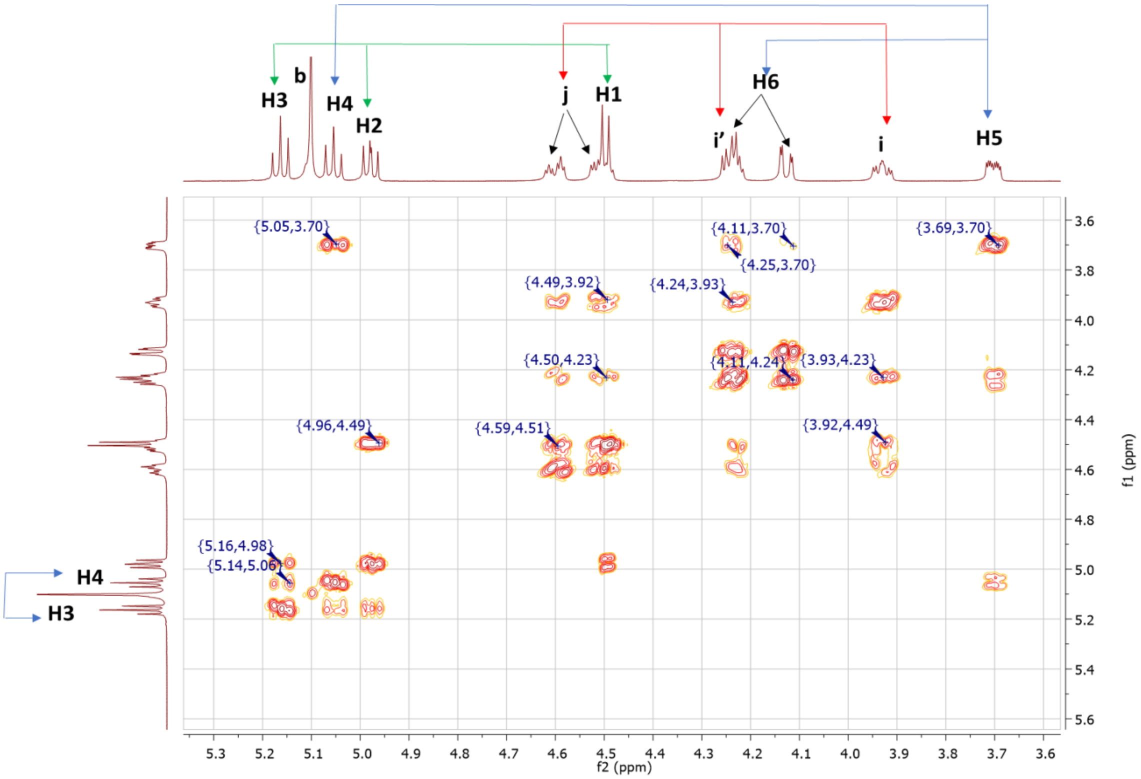

2D NMR – COSY: ^{1}H-^{1}H correlation spectrum of compound 35.

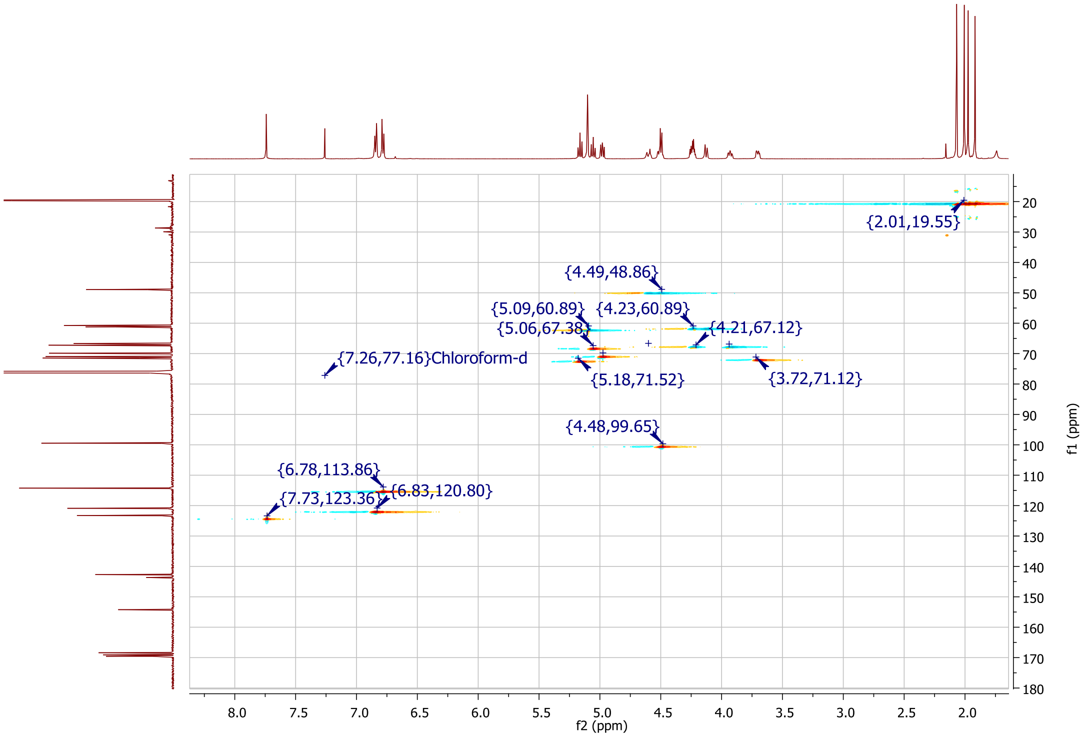

2D NMR – HSQC: ^{1}H-^{13}C correlation spectrum of compound 35.

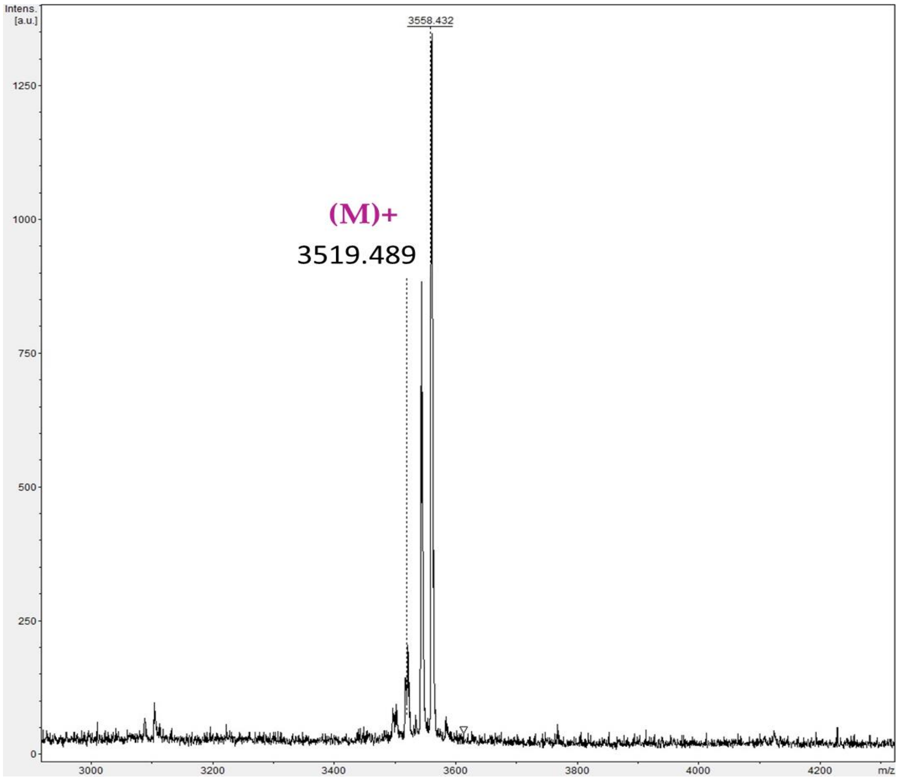

HRMS of compound 35.

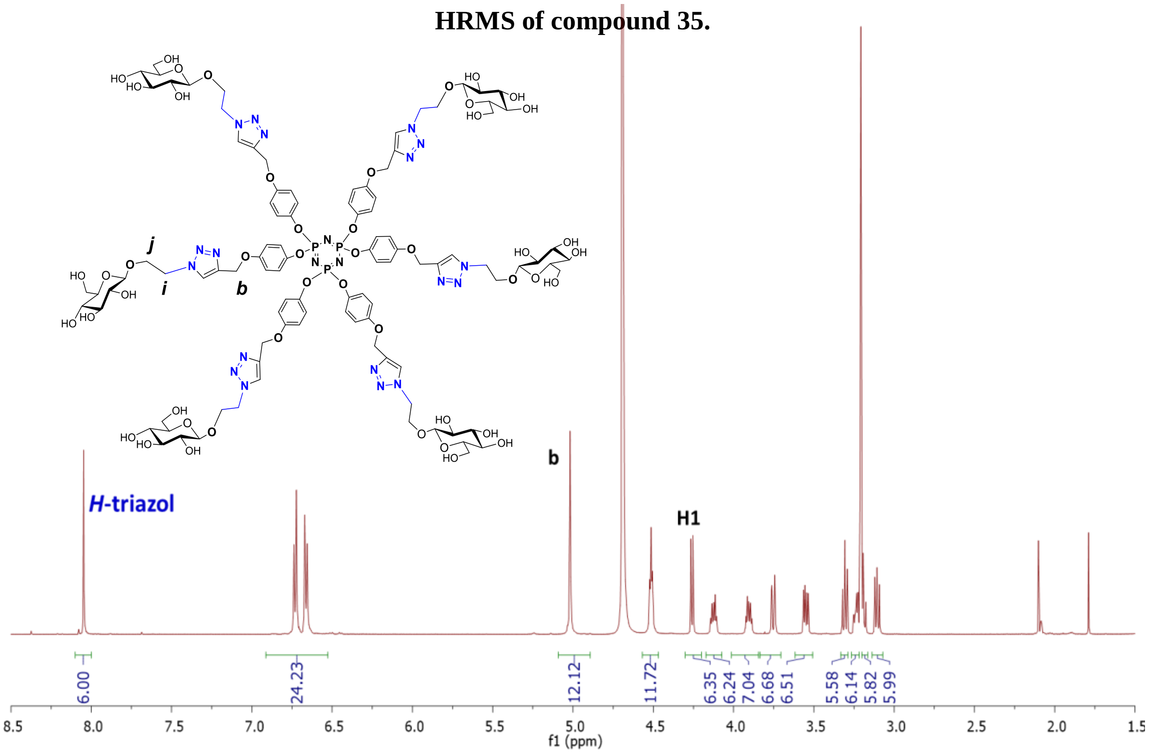

^{1}H- NMR spectrum of compound 36.

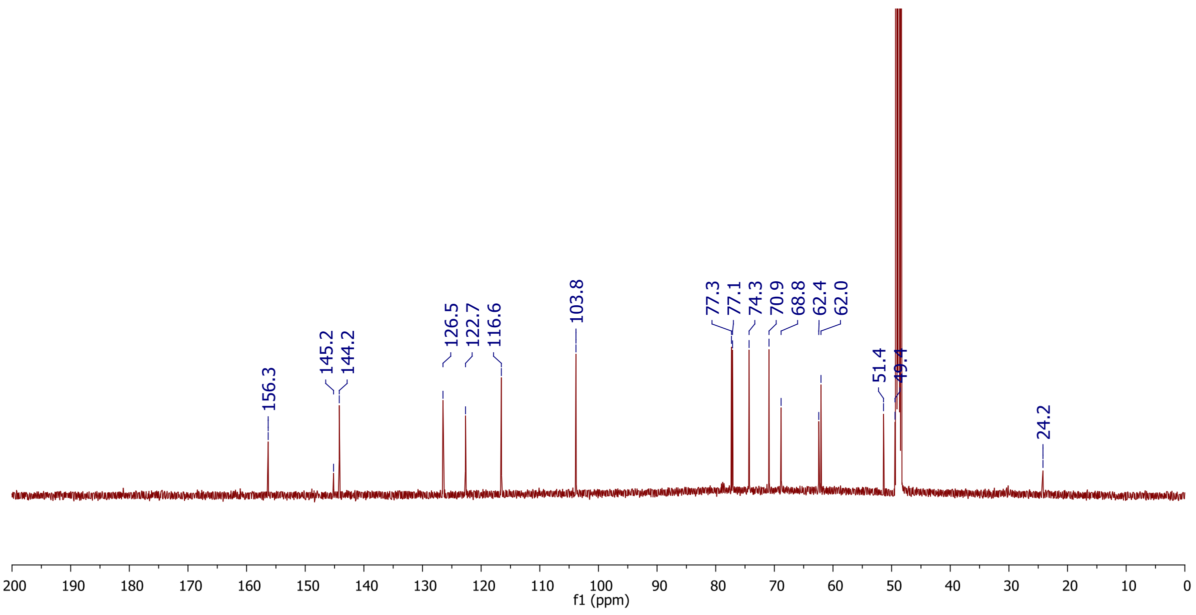

^{13}C- NMR spectrum of compound 36.

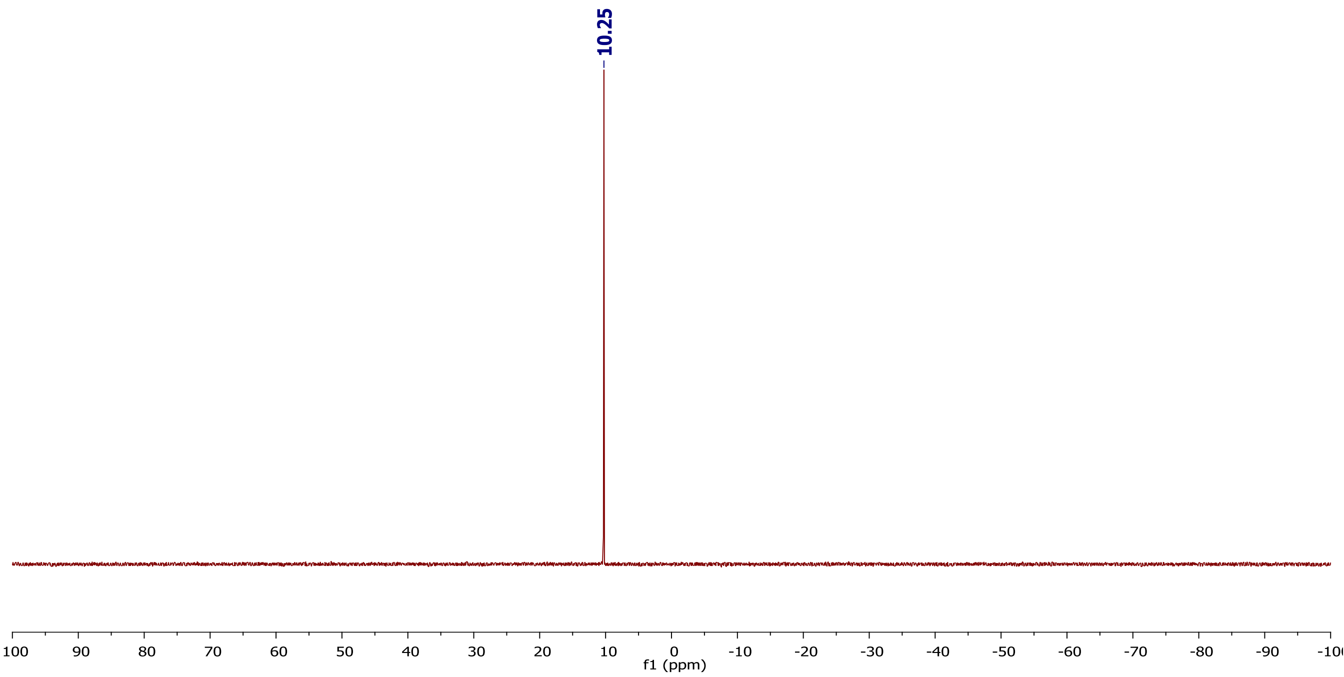

^{31}P- NMR spectrum of compound 36.

			Size (d.nm):	% Number:	St Dev (d.n...
Z-Average (d.nm):	85.49	Peak 1:	6.344	100.0	1.006
PdI:	0.579	Peak 2:	0.000	0.0	0.000
Intercept:	0.900	Peak 3:	0.000	0.0	0.000
Result quality :	Refer to quality report				

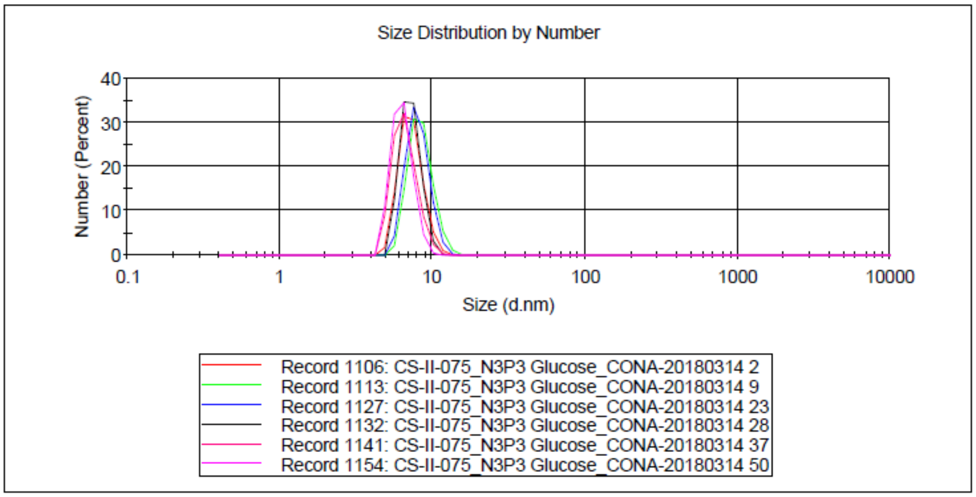

DLS- Qualitative Size distribution of compound 36 showing no cross-linking to Con A (no change in particle size).

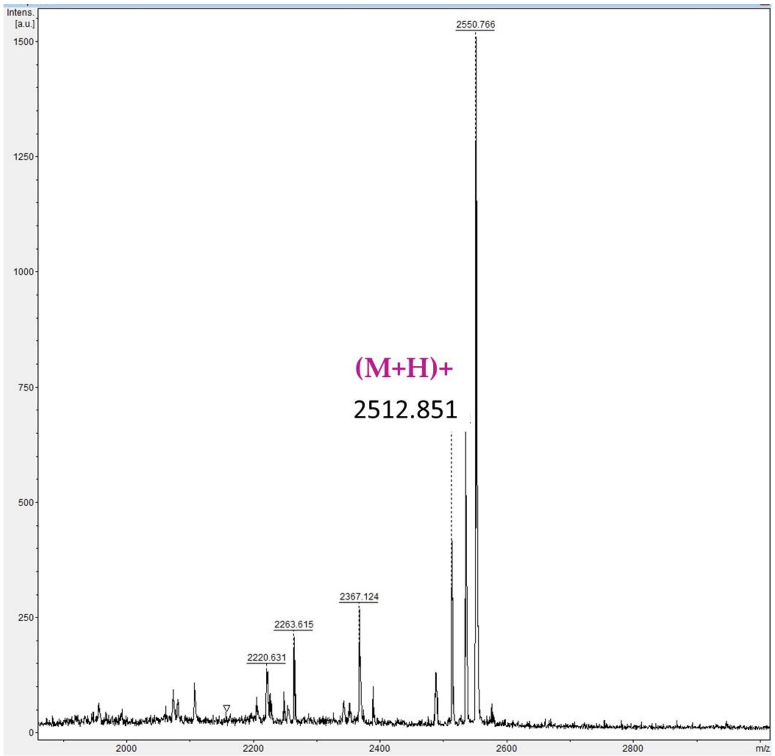

HRMS of compound 36.

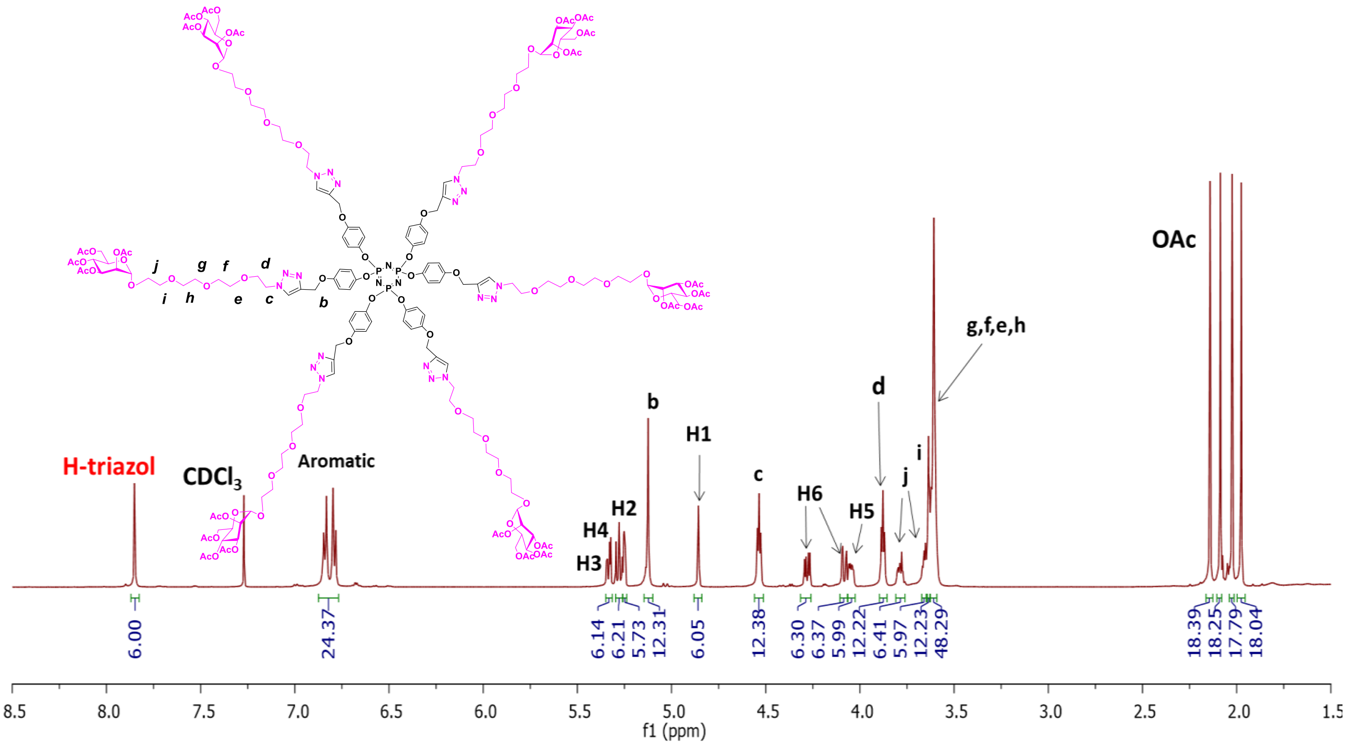

^{1}H- NMR spectrum of compound 37.

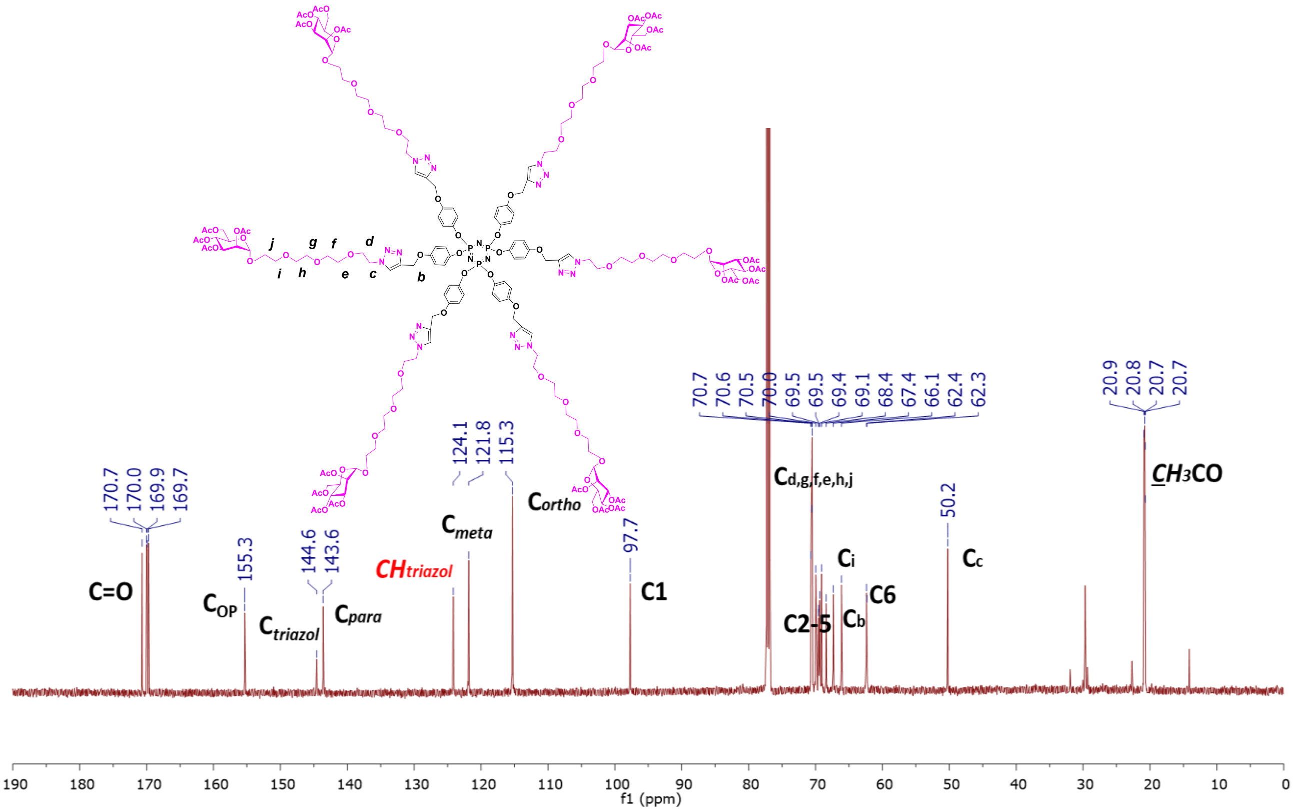

^{13}C- NMR spectrum of compound 37.

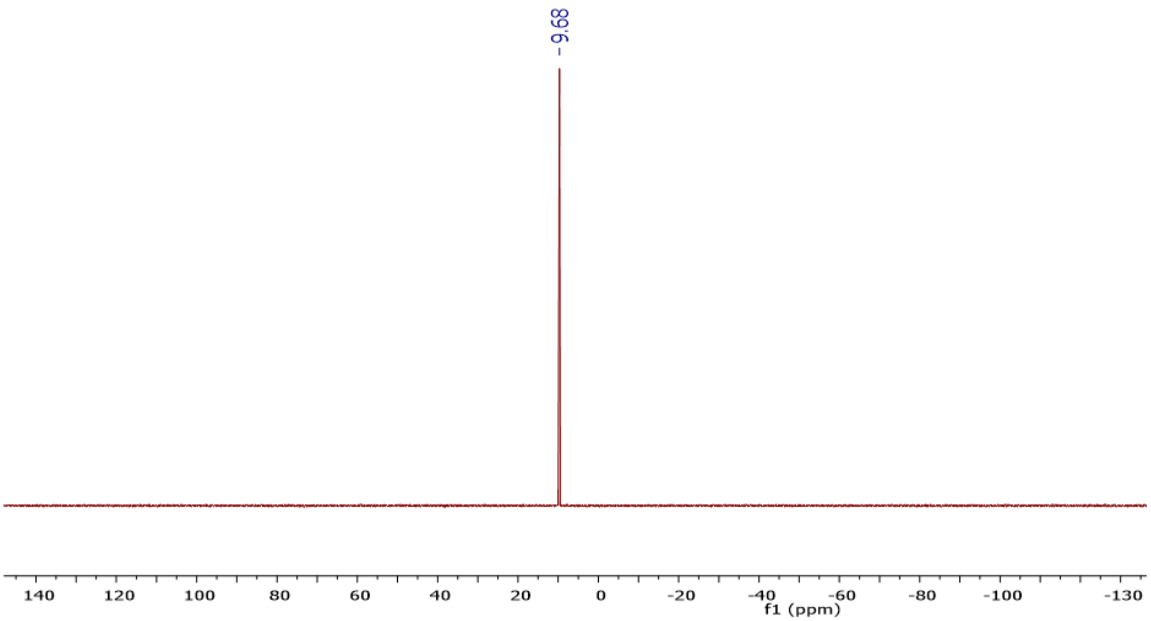

^{31}P- NMR spectrum of compound 37.

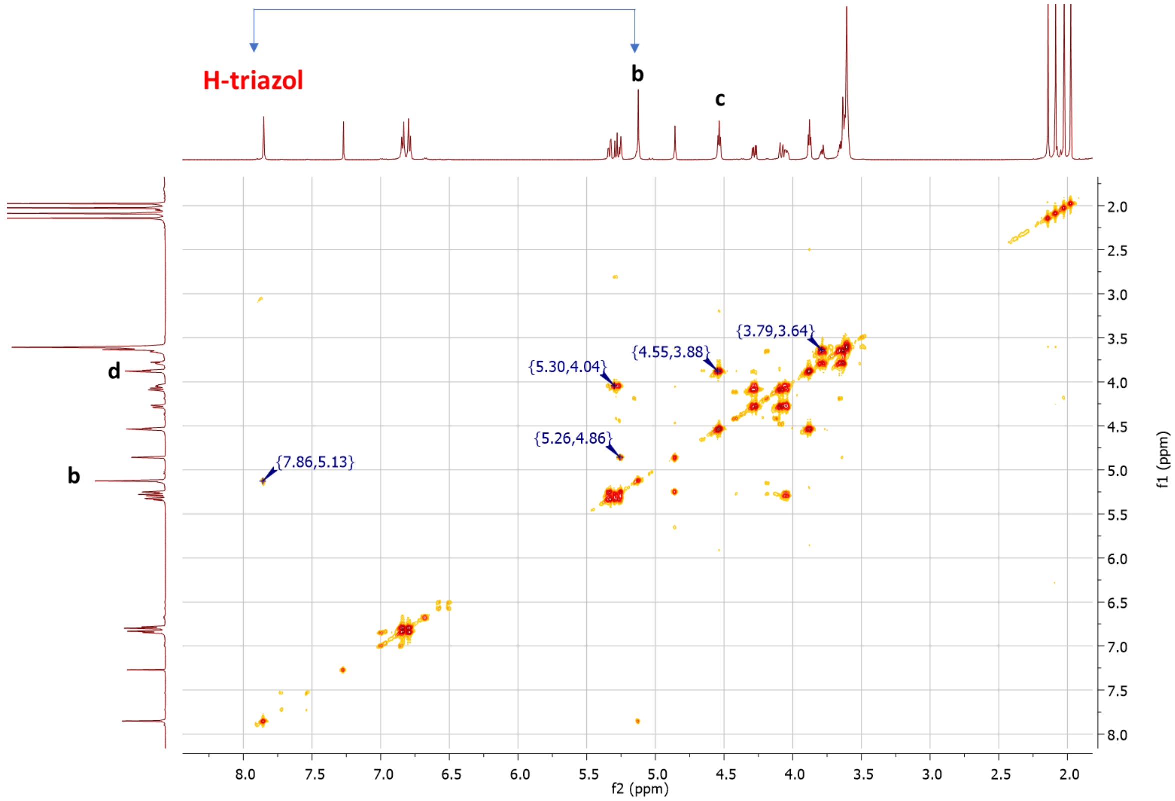

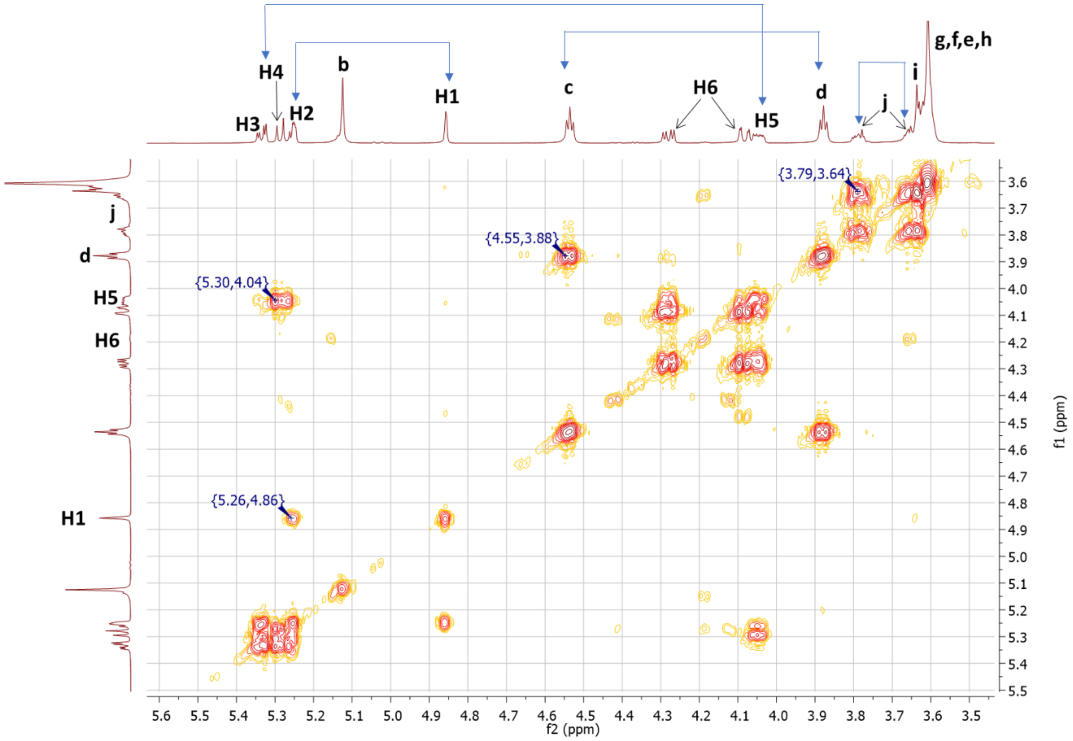

2D NMR – COSY: ^{1}H-^{1}H correlation spectrum of compound 37.

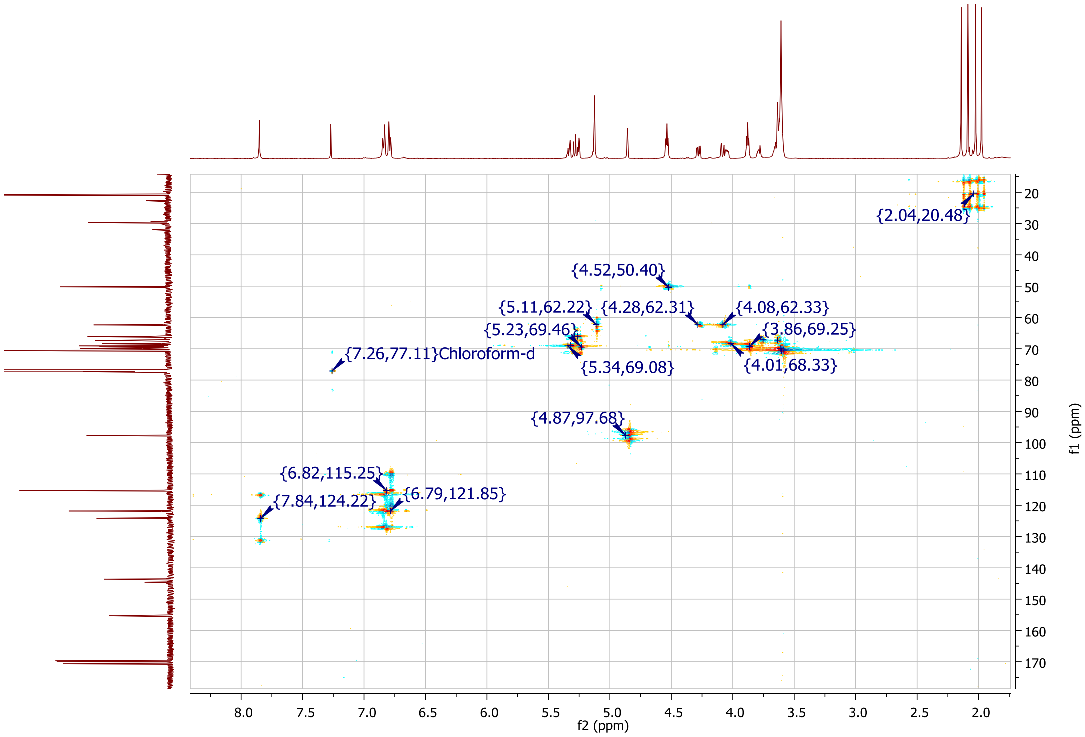

2D NMR – HSQC: ^{1}H-^{13}C correlation spectrum of compound 37.

Compound Table

Compound Label	RT	Mass	Abund	Formula	Tgt Mass	Diff (ppm)
Cpd 1: C186 H252 N21 O90 P3	0.1	4312.497	17070	C186 H252 N21 O90 P3	4312.5001	-0.71

Compound Label	RT	Algorithm	Mass
Cpd 1: C186 H252 N21 O90 P3	0.1	Find By Formula	4312.497

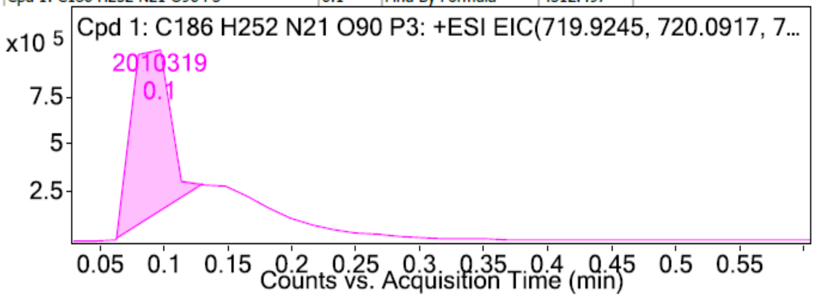

MS Spectrum

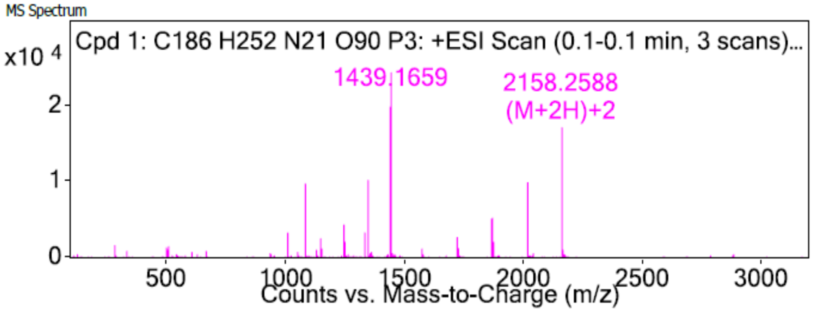

HRMS of compound 37.

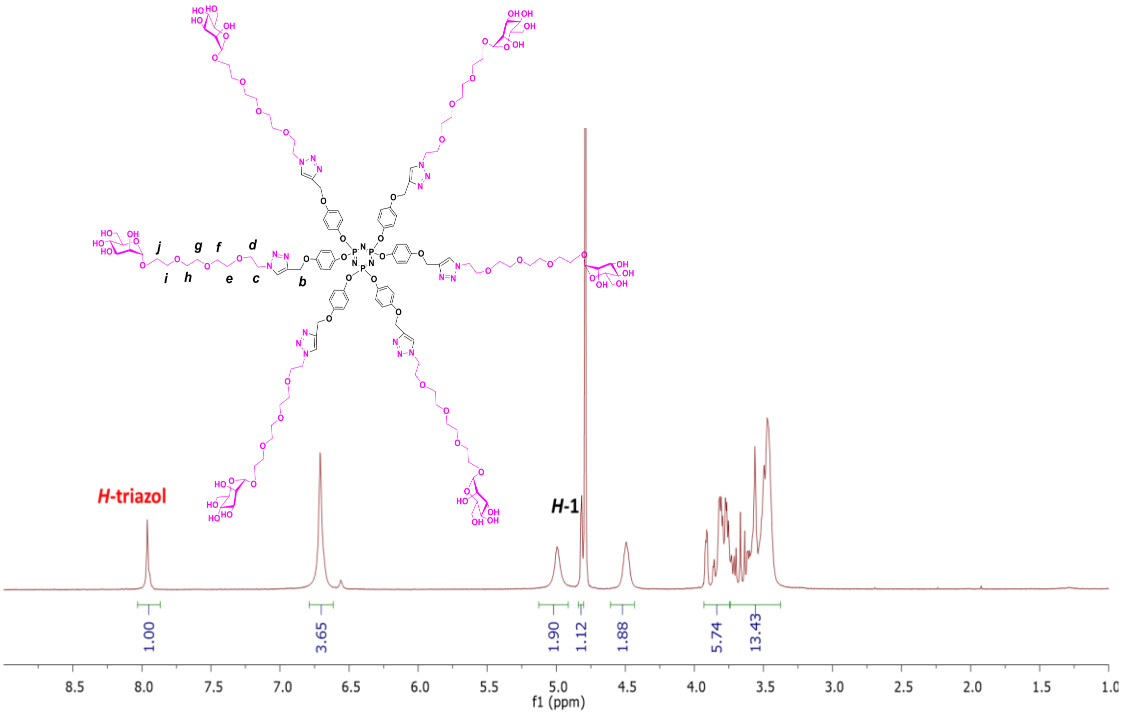

^{1}H- NMR spectrum of compound 38.

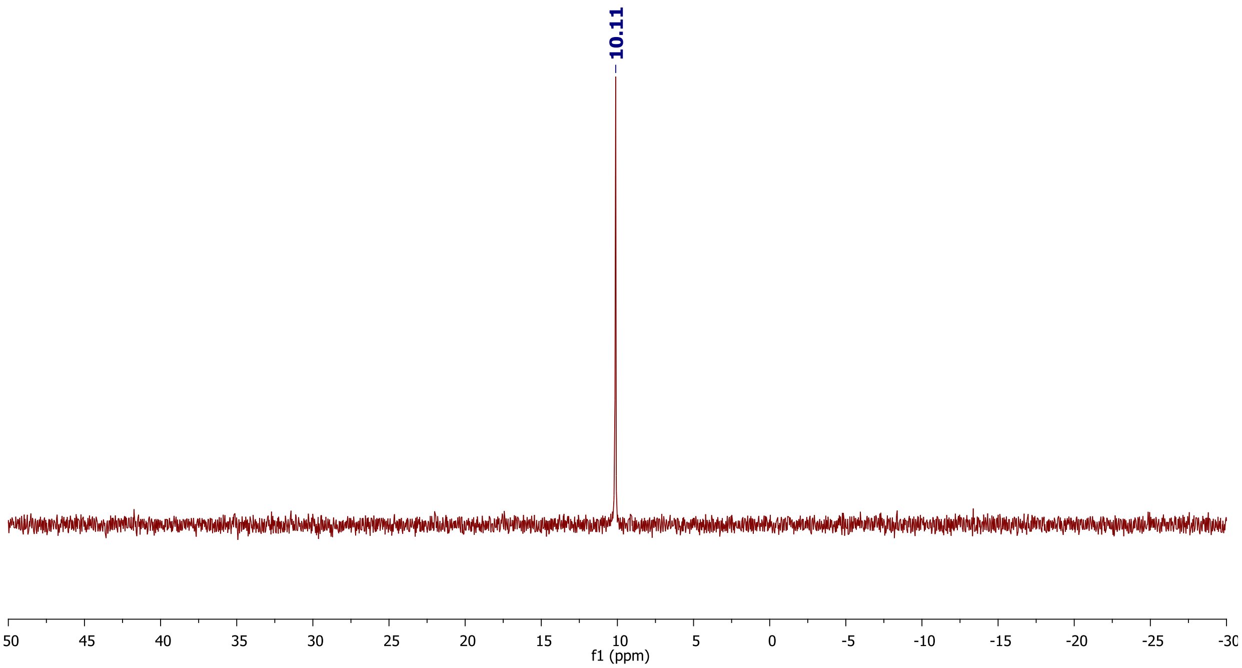

^{31}P- NMR spectrum of compound 38.

Compound Table

Compound Label	RT	Mass	Abund	Formula	Tgt Mass	Diff (ppm)
Cpd 1: C138 H204 N21 O66 P3	0.1	3304.2485	18873	C138 H204 N21 O66 P3	3304.2465	0.59

Compound Label	RT	Algorithm	Mass
Cpd 1: C138 H204 N21 O66 P3	0.1	Find By Formula	3304.2485

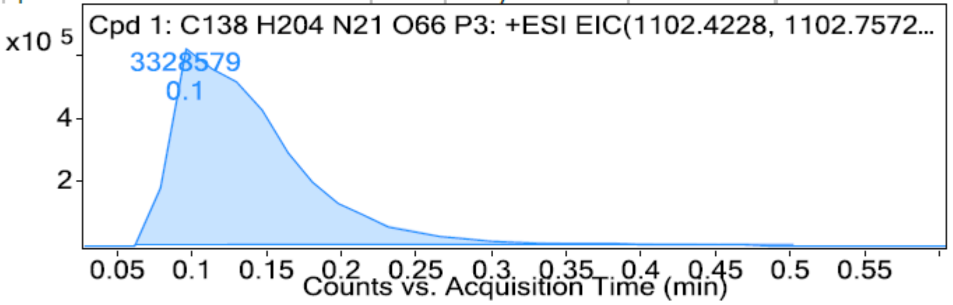

MS Spectrum

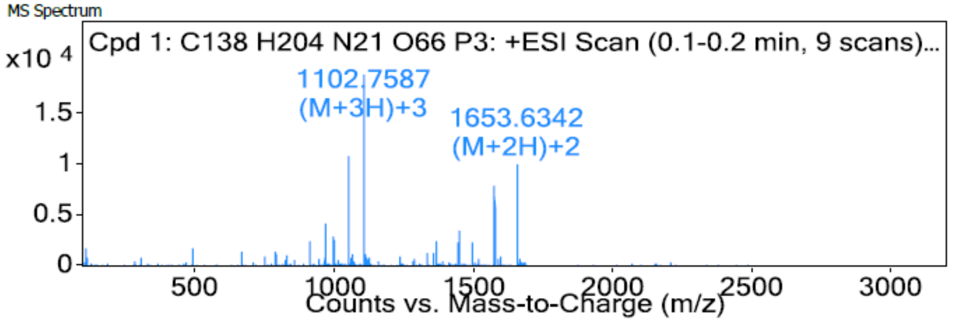

HRMS of compound 38.

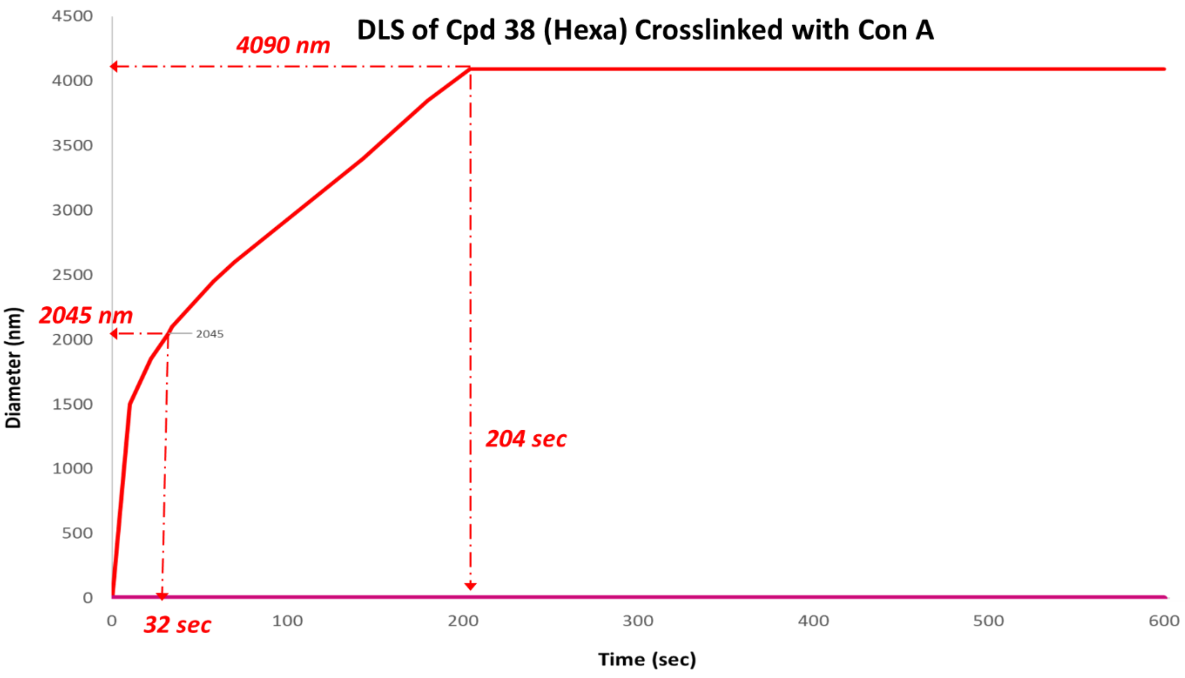

Fig 6. Synthesis DLS of Cpd **38** (Hexa) Crosslinked with Con A.

			Size (d.nm):	% Number:	St Dev (d.n...
Z-Average (d.nm):	3303	Peak 1:	4827	5.1	834.8
PdI:	0.574	Peak 2:	361.8	94.9	62.63
Intercept:	0.805	Peak 3:	0.000	0.0	0.000

Result quality : Refer to quality report

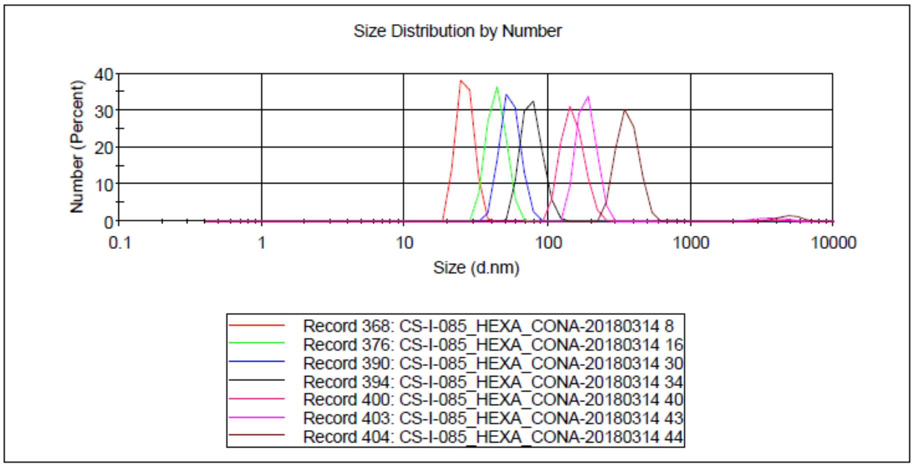

DLS-Size distribution of compound 38.

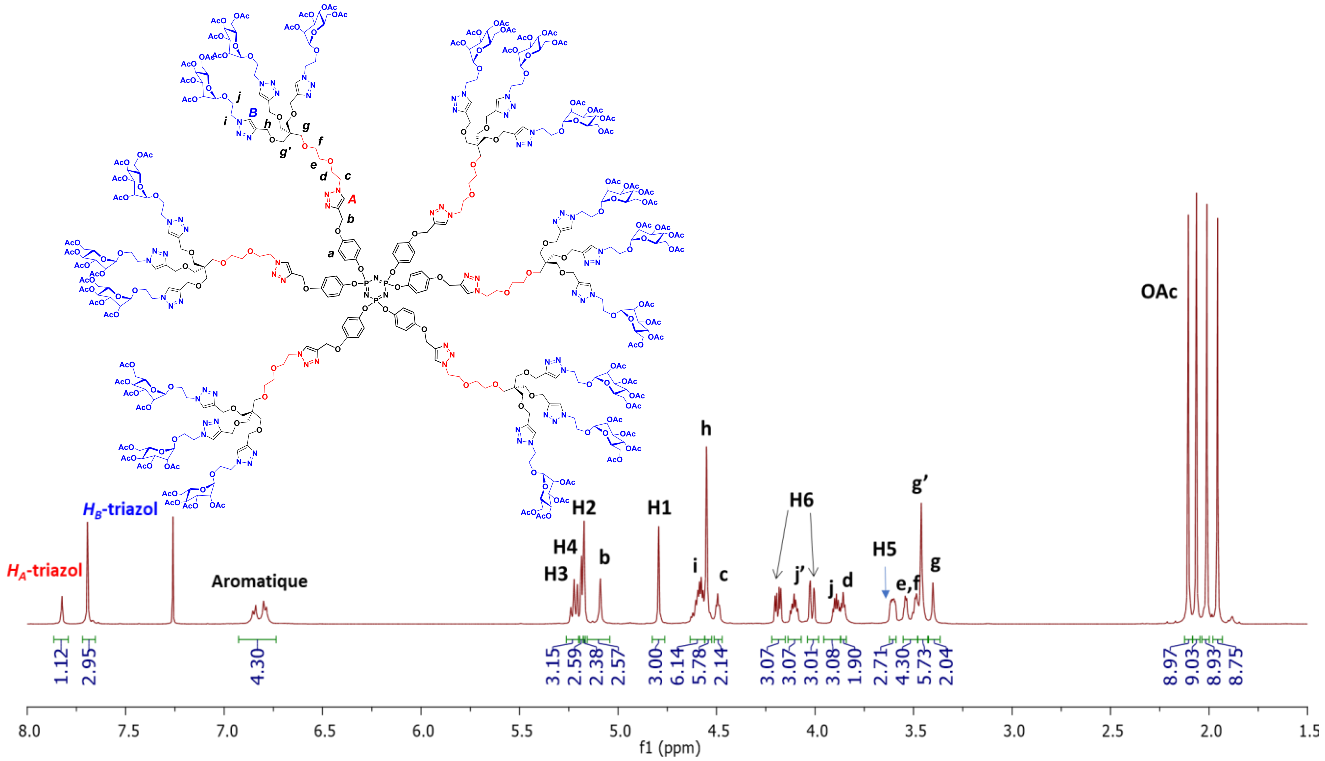

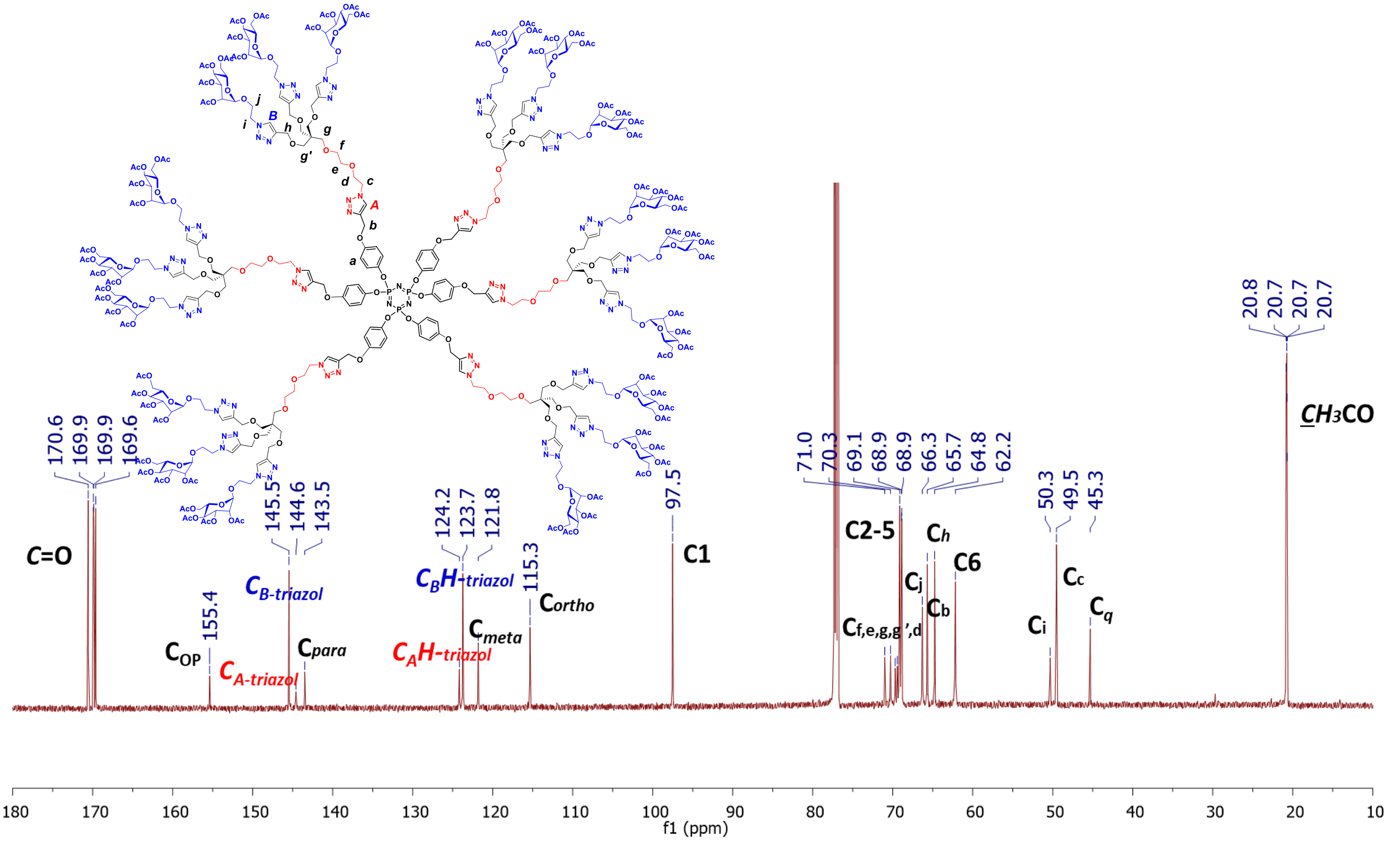

^{1}H- and ^{13}C- NMR spectrum of compound 39.

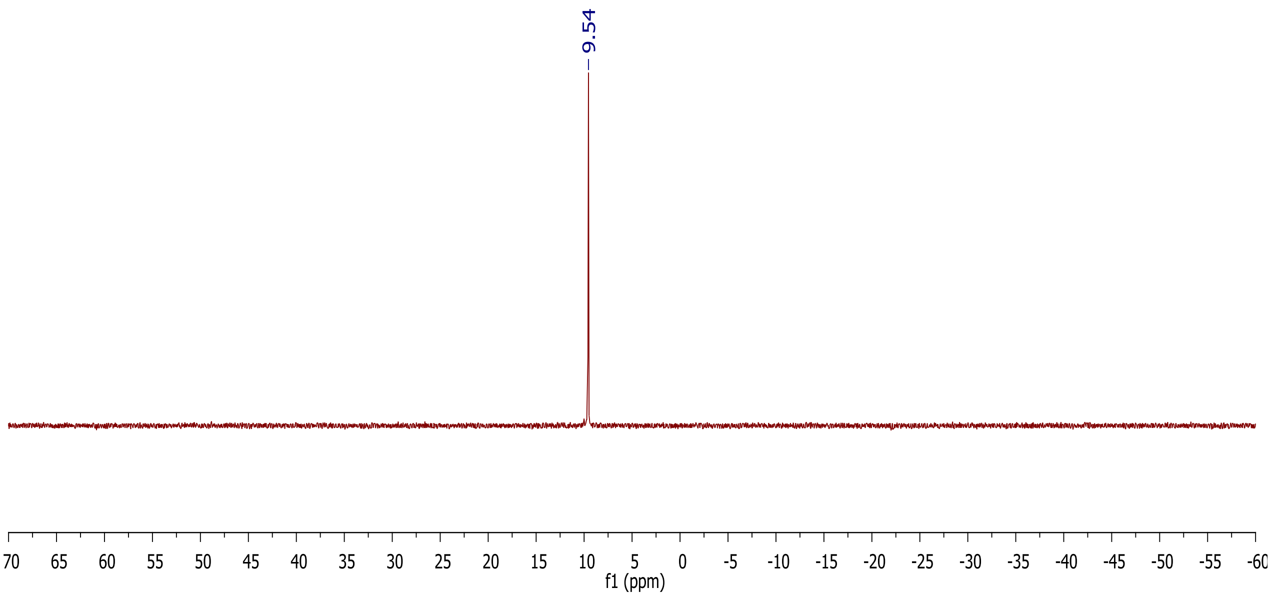

^{31}P- NMR spectrum of compound 39.

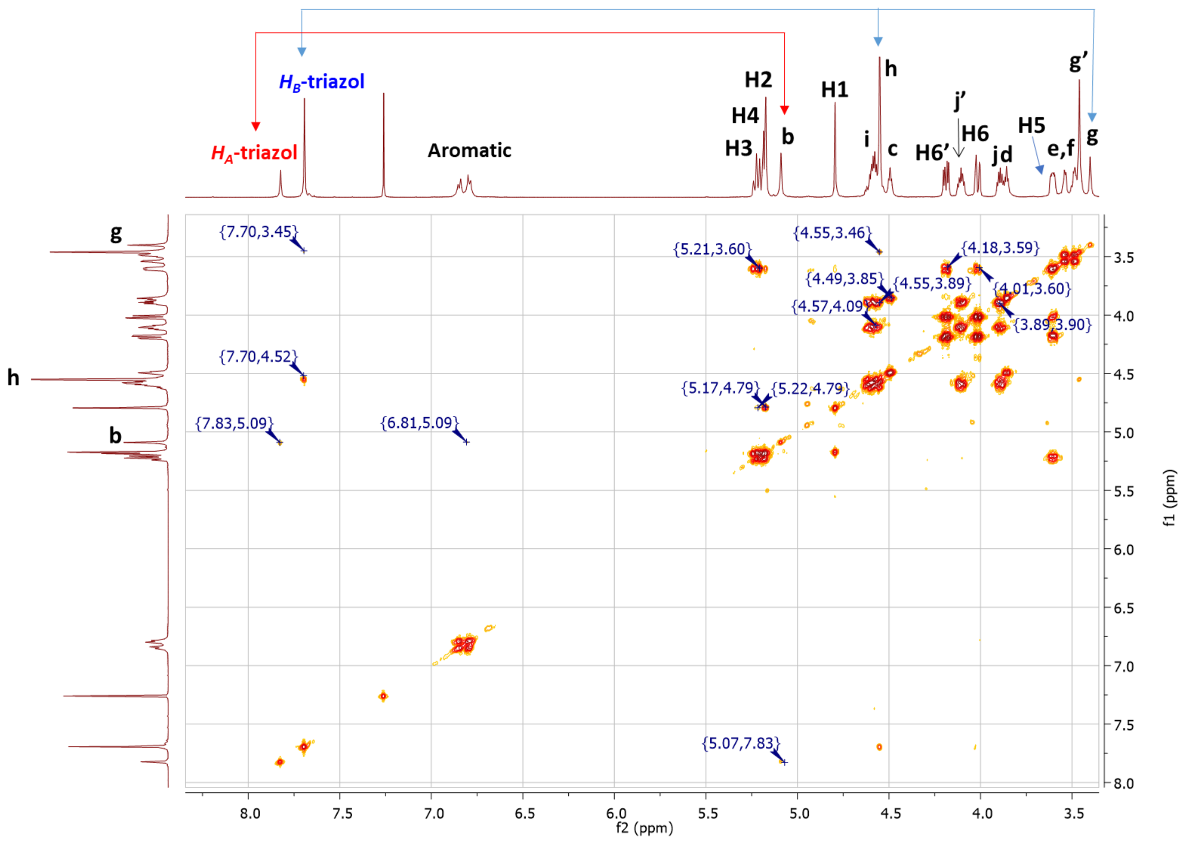

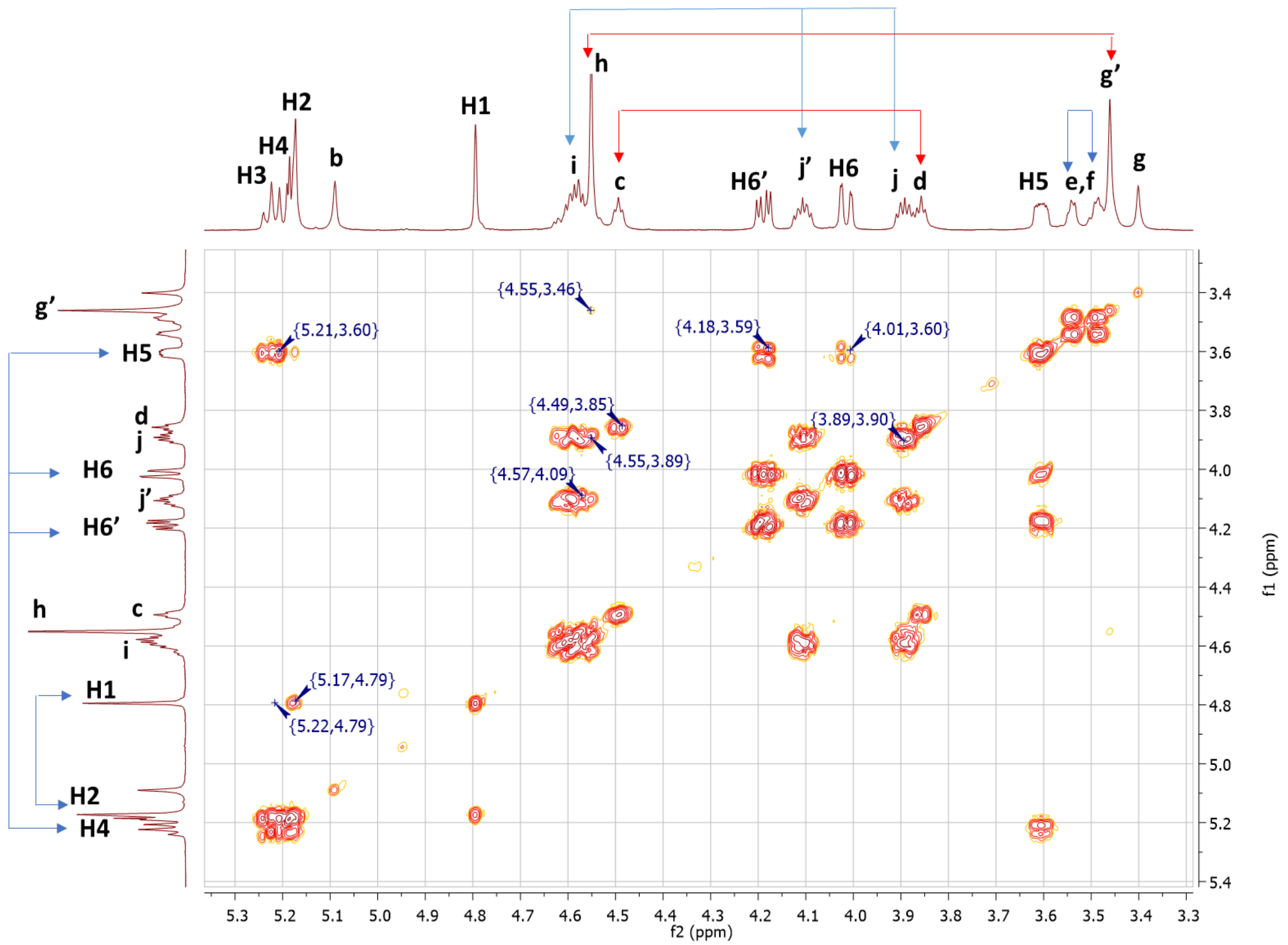

2D NMR – COSY: ^{1}H-^{1}H correlation spectrum of compound 39.

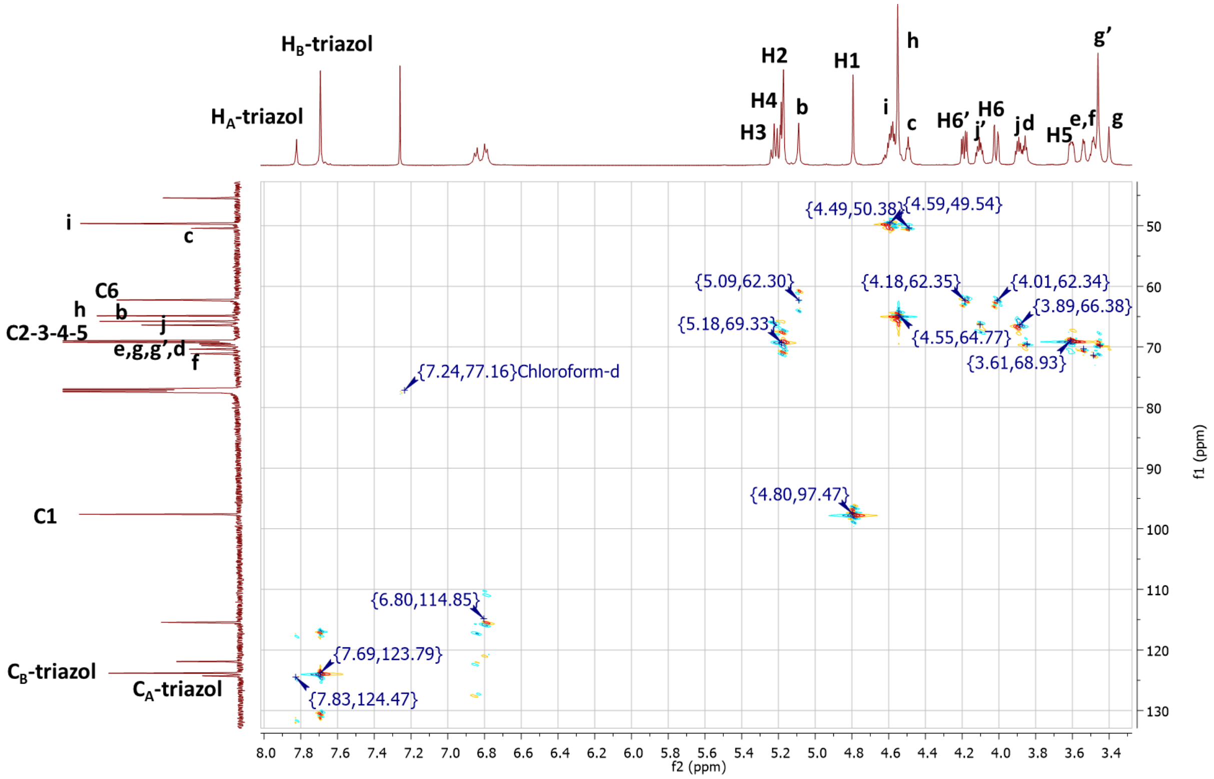

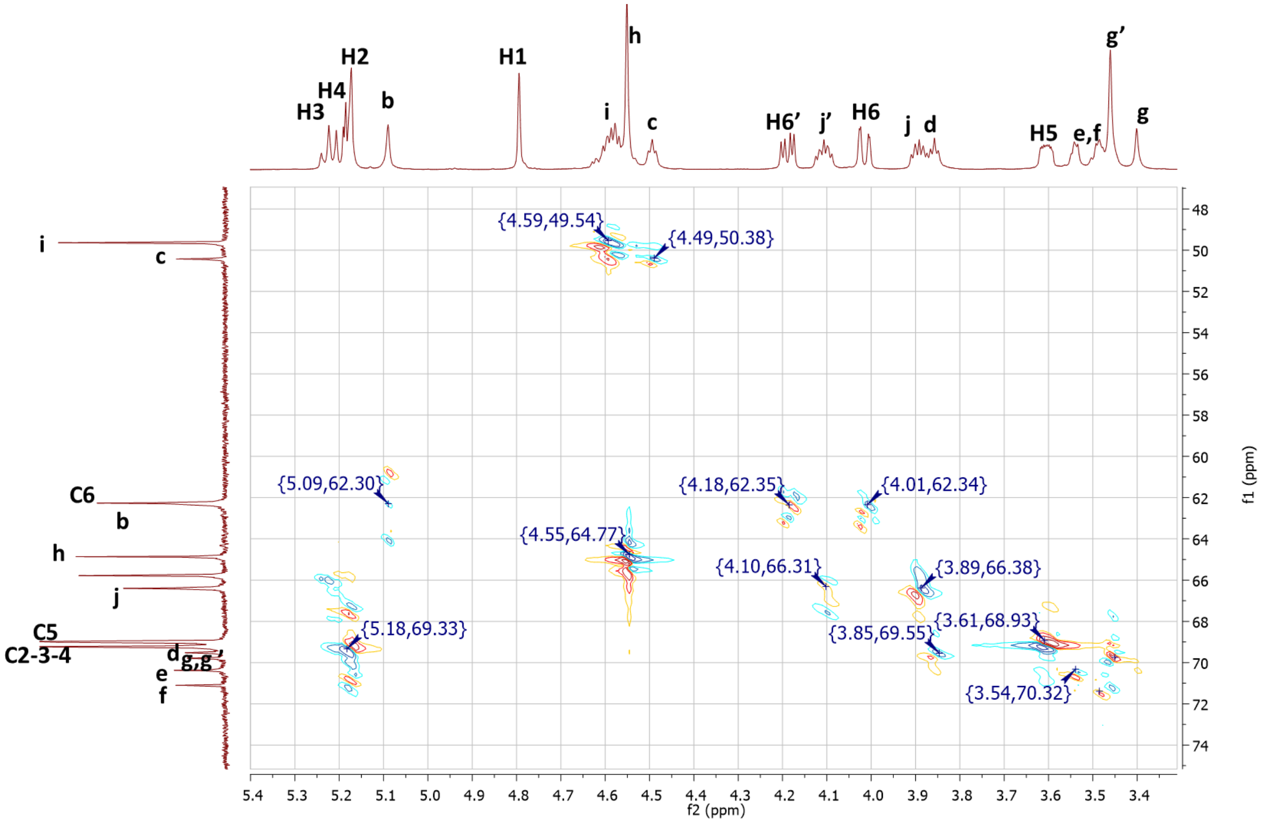

2D NMR – HSQC: ^{1}H-^{13}C correlation spectrum of compound 39.

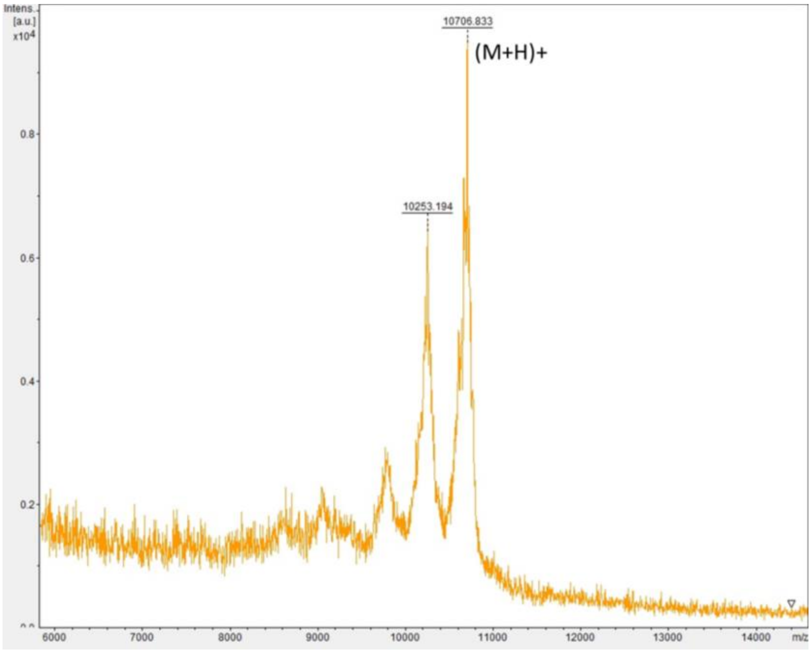

HRMS of compound 39.

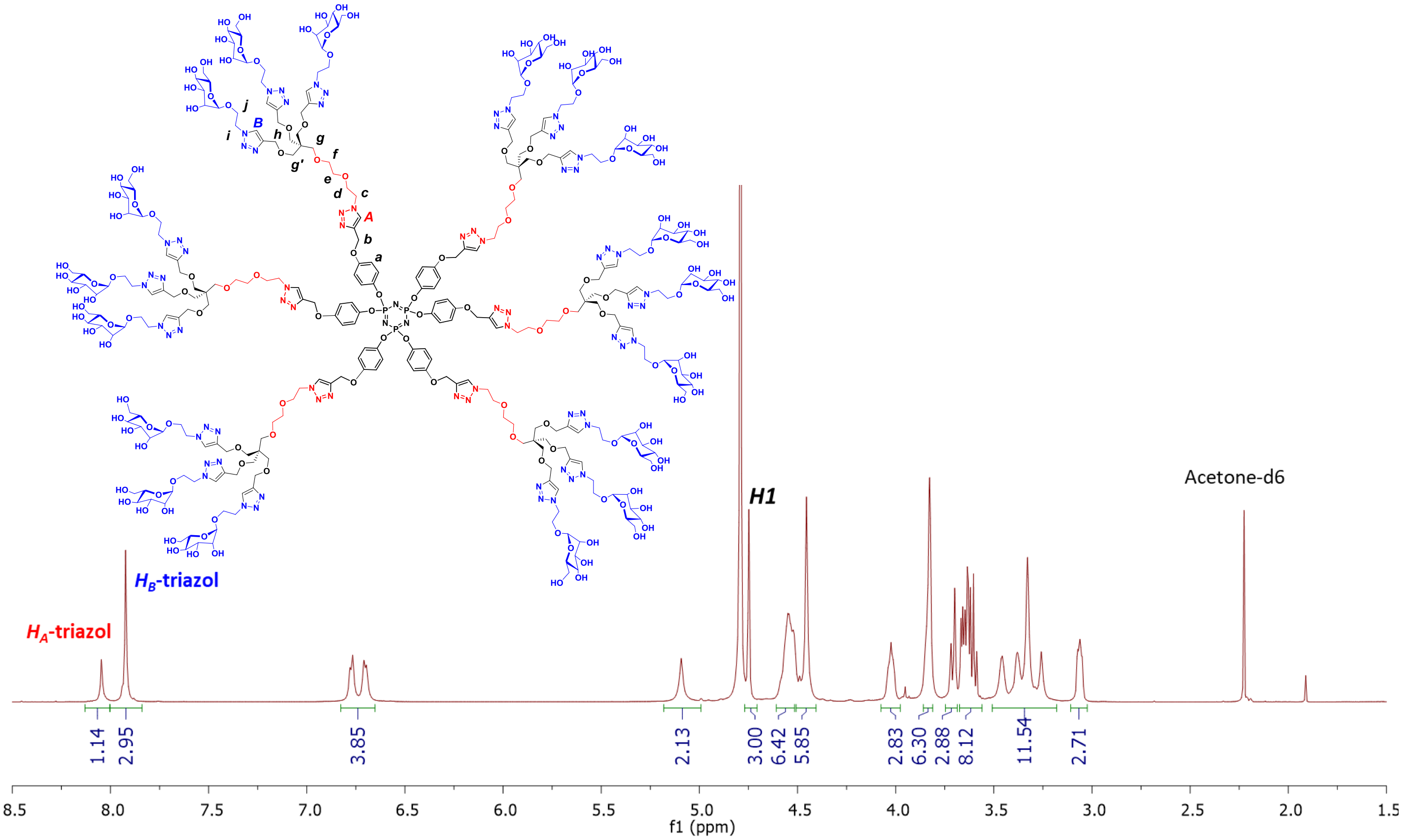

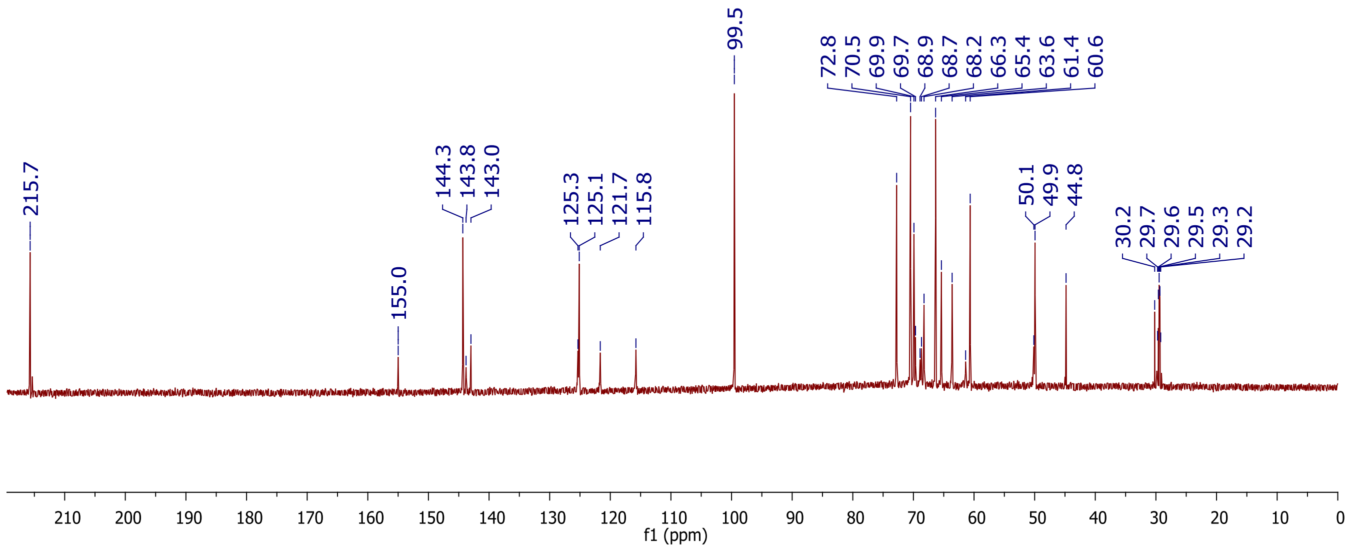

^{1}H- and ^{13}C- NMR spectrum of compound 40.

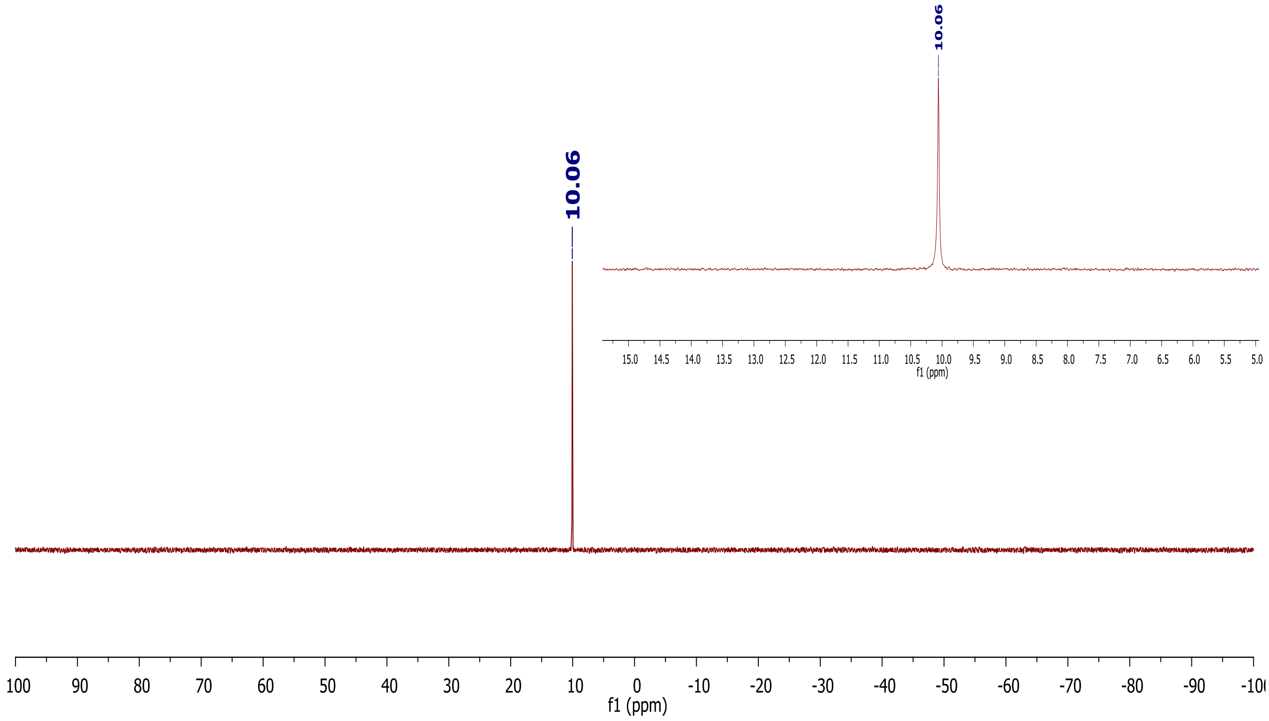

^{31}P- NMR spectrum of compound 40.

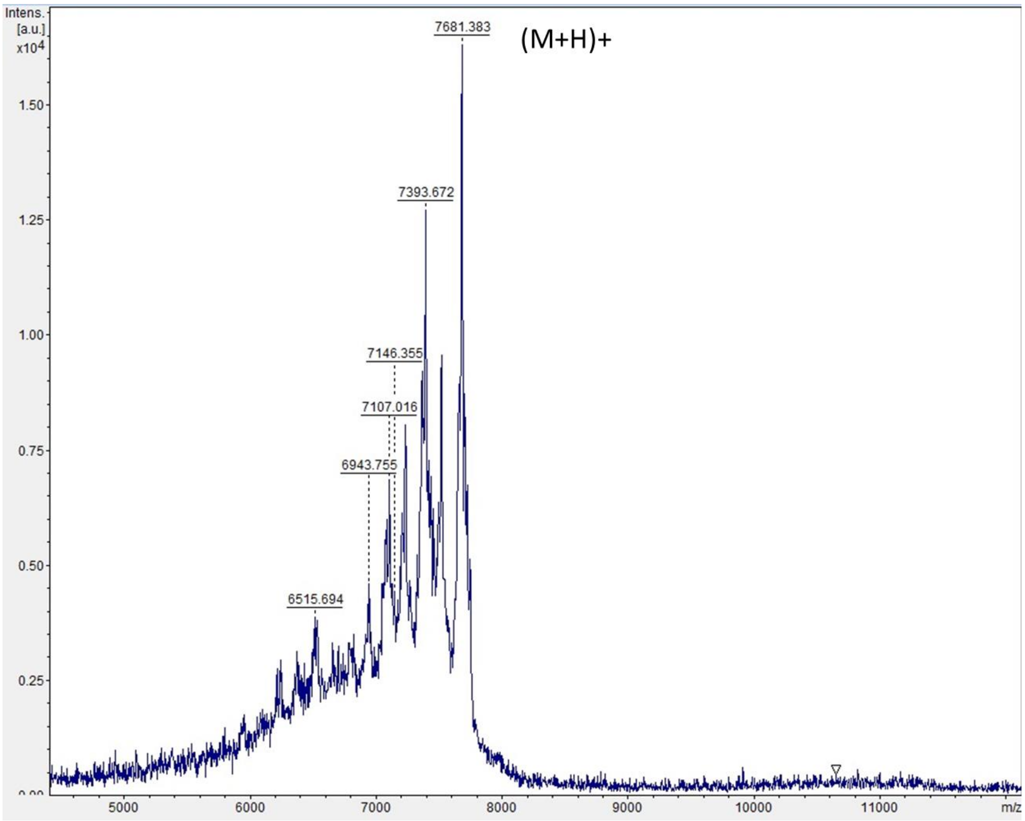

HRMS of compound 40.

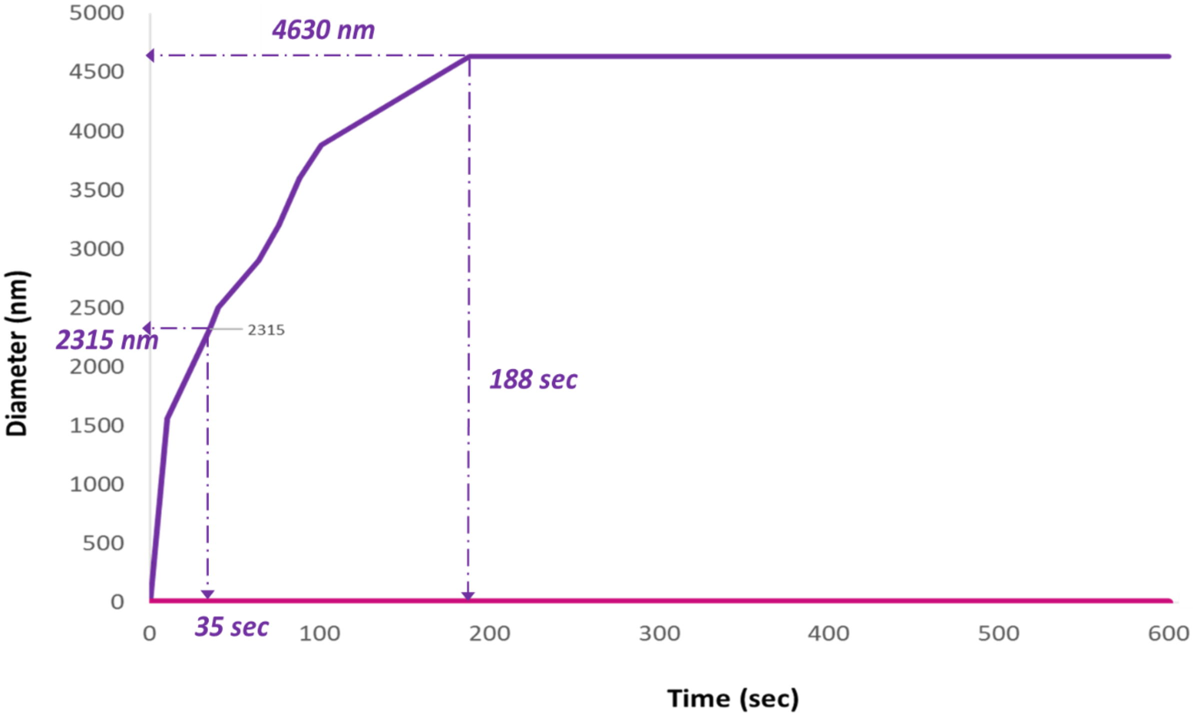

Fig 7. Synthesis DLS of Cpd **40** (G1) Crosslinked with Con A.

			Size (d.nm):	% Number:	St Dev (d.n...
Z-Average (d.nm):	5030	Peak 1:	1054	8.1	183.0
PdI:	0.525	Peak 2:	166.4	91.9	25.29
Intercept:	0.876	Peak 3:	0.000	0.0	0.000
Result quality :	Refer to quality report				

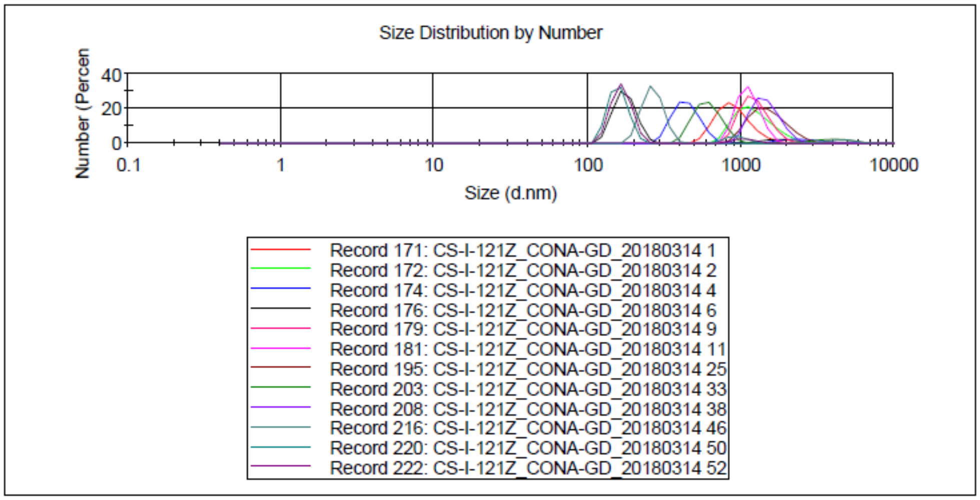

DLS of compound 40.

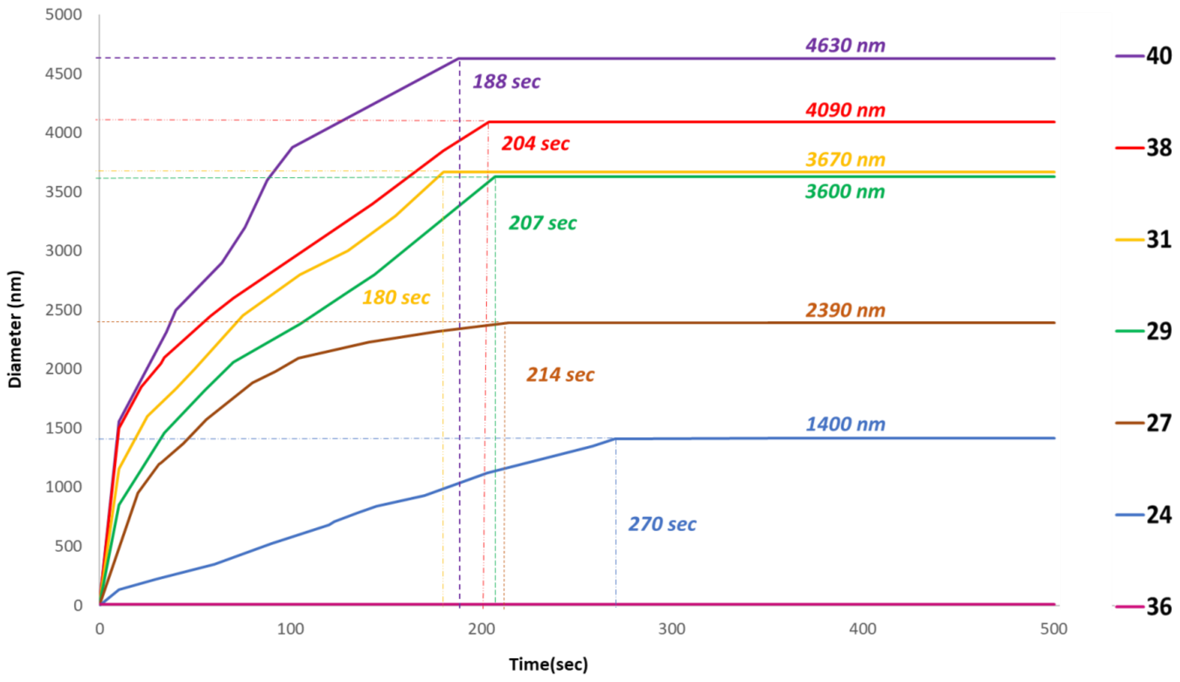

Fig 2. DLS of Cpds **24**; **27**; **29**; **31**; **38** and **40** Crosslinked with Con A.

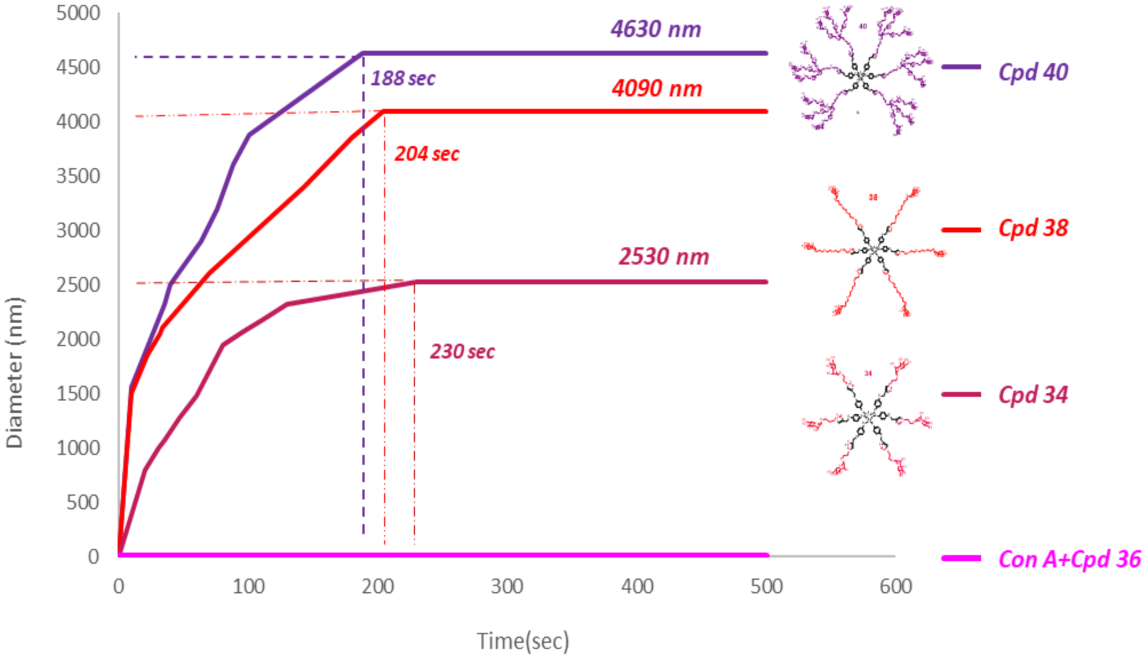

Fig 3. DLS of phosphorylated Cpds **34**; **36**; **38** and **40** Crosslinked with Con A.
